# Seaweed as a Natural Source against Phytopathogenic Bacteria

**DOI:** 10.3390/md21010023

**Published:** 2022-12-28

**Authors:** Tânia F. L. Vicente, Carina Félix, Rafael Félix, Patrícia Valentão, Marco F. L. Lemos

**Affiliations:** 1 MARE-Marine and Environmental Sciences Centre & ARNET—Aquatic Research Network Associated Laboratory, ESTM, Polytechnic of Leiria, 2520-641 Peniche, Portugal; 2 REQUIMTE/LAQV, Laboratório de Farmacognosia, Faculdade de Farmácia, Universidade do Porto, 4050-313 Porto, Portugal

**Keywords:** agriculture, antibacterial potential, biotechnology, epigenetics, phenolic compounds, priming elicitors, sulfated compounds

## Abstract

Plant bacterial pathogens can be devastating and compromise entire crops of fruit and vegetables worldwide. The consequences of bacterial plant infections represent not only relevant economical losses, but also the reduction of food availability. Synthetic bactericides have been the most used tool to control bacterial diseases, representing an expensive investment for the producers, since cyclic applications are usually necessary, and are a potential threat to the environment. The development of greener methodologies is of paramount importance, and some options are already available in the market, usually related to genetic manipulation or plant community modulation, as in the case of biocontrol. Seaweeds are one of the richest sources of bioactive compounds, already being used in different industries such as cosmetics, food, medicine, pharmaceutical investigation, and agriculture, among others. They also arise as an eco-friendly alternative to synthetic bactericides. Several studies have already demonstrated their inhibitory activity over relevant bacterial phytopathogens, some of these compounds are known for their eliciting ability to trigger priming defense mechanisms. The present work aims to gather the available information regarding seaweed extracts/compounds with antibacterial activity and eliciting potential to control bacterial phytopathogens, highlighting the extracts from brown algae with protective properties against microbial attack.

## 1. Introduction

Plant pests represent a growing problem concerning not only producers but also the scientific community due to the annual losses of crops and the consequent high economic impact on the food market [1]. From the total expenses in the agricultural industry, 31 billion USD (near to a quarter of the total) are used to mitigate plant pests [2]. The economic impact of bacterial phytopathogens can reach over 1 billion USD a year [3,4]. In addition to the increase of microbial infections in agricultural species, the increase in the world population (growth of 9 billion people estimated for the next 30 years [5,6]) is also a huge concern due to the possible unavailability of food supplies for future generations [3].

Among the most relevant phytopathogens, the damage caused by bacterial microorganisms must be highlighted [5]. These types of infections have been classified as one of the most damaging to crops, due to their harmful effects on plants, damaging fruits and leaves [6] or the whole plant system [7]. Since their first report in 1932, which accounted for 25–75% of the losses of peach, reports of infections caused by phytopathogenic bacteria have increased [4]. The lack of current totally efficient/safe techniques/products allows the proliferation of bacteria and their adaptation to overcome the plant’s intrinsic defense pathways [8]. In addition, there are external factors that contribute to the acquisition of advantageous characteristics by these pathogens. Current climatic changes occurring all over the world can raise perfect niches with suitable conditions for the genetic improvement and expansion of phytopathogenic bacteria [9,10,11]. Based on the assumptions of Harvell [12] about the constant increase of temperature, the maintenance of plant pathogens through different seasons is expected and may also contribute to the adaptation of different bacterial species to different environments [11]. Some bacterial phytopathogens present a high capacity for adaptation and physiological versatility, allowing their survival even in the absence of a host plant [13].

The use of synthetic agrochemicals possessing antibiotic properties is currently the most effective approach against phytopathogenic bacteria [6]. However, this kind of product presents several limitations. It demands a continuous application to be efficient, which not only can be expensive for the farmers but also environmentally harmful for the non-target species [14,15]. In addition, the use of these agrochemicals is not completely efficient due to the great capacity of bacteria to create resistance to the applied products, overcoming their initial toxicity [16,17]. This scenario leads to a constant search for innovative, more efficient, and sustainable options, in order to protect the crops and the remaining non-target biodiversity while being safe for human consumption [18].

As a sustainable option, biocontrol arises as a promising methodology, characterized by the introduction of an antagonist species in the affected environment that competes with the target phytopathogenic bacteria [19,20], limiting the bacterial phytopathogen population [21,22]. Several studies presented a wide range of microbial options, including growth-promoting bacteria beneficial for the plant [19,23,24,25] and bacteria naturally present in the plant microbiota, such as Rhizobacteria, with antibacterial properties [26]. *Bacillus*, *Pseudomonas* [27,28], *Enterococcus* [29], *Burkholderia* [30], *Lactococcus*, *Streptomyces*, *Klebsiella*, and *Escherichia* [25] are among the genera with biocontrol potential. Secondary metabolites like Non-Ribosomal Peptides (NRPs) have been associated with biocontrol through the activation of mechanisms associated with plant defense [28], while environmental factors, such as humidity, temperature, and pH of the soil, are important parameters that can affect the success of biocontrol, which makes this method suitable only in specific occasions [20,31]. Additionally, the competition with bacterial species naturally present in the microbiota of the host, as well as their age, are determinants to promote/repress the expression of genes [32] that play a crucial role in biocontrol action [33,34,35].

Genetic manipulation aiming at plant improvement has also been employed with good results demonstrated in the reduction of infection symptoms [36]. However, the appearance of transgenic species with a specific high resistance can be a problem, due to their ineffectiveness against the attack of multiple bacteria, in addition to the consumer’s reluctance to accept genetically manipulated species [8].

In this context, looking at the available solutions and respective limitations, the continuous development of new, effective, and safer methods to combat infection and the emergence of bacterial phytopathogens are crucial. Pursuing sustainable and eco-friendly alternatives to the present problem, marine habitats are an interesting source of bioactive and valuable compounds known to be applied with different industrial purposes [37,38,39] is an interesting starting point.

Seaweeds are spread all over the ocean [37,40] and are one of the most attractive and richest sources of bioactive compounds in the marine environment [41]. Several studies point to a set of macroalgae compounds possessing different properties, such as phenolic compounds, polysaccharides, and derivatives, lipids, sterols, pigments [42], terpenoids, lectins, alkaloids, including halogenated compounds, among others [43,44,45]. Their exploitation is already vast in some industries, encompassing the food, cosmeceutical, and agricultural industries [42], but the search for other bioactivities has also been growing. One of them is the antimicrobial activity from algae compounds against phytopathogens [41,46], which remains poorly described against bacterial phytopathogens when compared with the amount of data reported regarding antibacterial activity against human pathogens [47]. Several compounds’ families have exhibited antimicrobial activity against a wide range of phytopathogens, such as pigments (carotenoids), fatty acids, sterols, terpenes, polysaccharides, phenolic compounds, proteins, and peptides [44,45,48].

On the other hand, an improvement of plant resistance against these microbial phytopathogens has been suggested [49]. It is known that, since early times, seaweeds have been applied in agriculture due to the valuable advantages they confer to crops as growth promoters, rendering high-quality products with healthier and visually more attractive characteristics [50,51,52,53]. In addition, there are approved and commercial products based mostly on the brown alga *Ascophyllum nodosum* that are applied for a wide range of agricultural purposes [51]. This species is nutritionally rich, improving the soils where the plants are growing and can be helpful to regulate the plant in hostile conditions such as high salinity soils, drought stress, and low temperature tolerance, which can confer strong roots, increasing antioxidants, nutrient uptake, and consequently high-quality fruits [51]. Their capacity to improve plant growth and stimulate the defense pathways against biotic and abiotic threats are the most reported in the literature [54,55,56]. Accounting for the security of some bioactive algae extracts to plants, the currently commercialized, and also other species, are currently being explored as potential elicitors to stimulate the natural mechanisms of resistance against bacterial invasion [57]. This protective role of algae extracts, usually associated with the presence of polysaccharides, phenolic compounds [6], and sulfated compounds [58] in crops, has been mostly demonstrated through the elicitation of priming events. This complex process consists of the stimulation of natural plant defense mechanisms, improving the plant responses against microbial attacks, through the expression of specific pathogenesis-related genes, responsible for plant defense and the consequent control of the damages [58]. Some of these genes can be translated into enzymes with degradative capacity over microbial compositional components, avoiding their development. Additionally, some of the resulting products of this degradation can act as activators of disease resistance [6], resulting in a “cyclic” defense mechanism.

Considering the problematic bacterial infections in plants and the lack of efficiency and/or sustainability and safety of the current methods, macroalgae-derived compounds appear as promising antibacterial/eliciting tools. Then, this review aims to gather the maximum available information regarding seaweed extracts presenting not only potential against bacterial phytopathogens but also studies demonstrating the plant eliciting capacity to face these bacterial invasions and molecular mechanisms involved.

## 2. Material and Methods

This literature revision includes the available information regarding the antibacterial and/or plant-priming activity of seaweed until 26 October 2022, using the SCOPUS database (www.scopus.com). The search was performed using a combination of the following words: “Antibacteria* AND (Plant* OR crop* OR agricultur* OR veget* OR phytopatho*) AND (Macroalga* OR seaweed)”, to compile the works aiming at the algae extracts with antibacterial potential/activity against bacterial phytopathogens. In addition, for the review of works reporting the priming promotion of macroalgae extracts on plants, the following combination of words “Microb* OR bacter* AND (Fitness OR immun* OR defen* OR elicit*) AND (Plant* OR crop* OR agricultur* OR veget* OR phytopatho*) AND (Macroalga* OR seaweed)” were used.

## 3. Phytopathogenic Bacteria

Currently, more than 200 phytopathogenic bacteria species have been reported [59,60], and the majority of these pathogens are phytobacteria [61] included in the phylum Proteobacteria [8]. Some species of plant pathogenic bacteria have a great capacity to adapt to different environments, allowing the extension of host possibilities. Species belonging to *Pseudomonas* spp. (highlighting *Pseudomonas aeruginosa*) are a good example of this, being able to infect even different kingdoms and constituting a threat also for humans [62].

From the bacterial species known to be aggressive phytopathogens, it is important to highlight not only the *Pseudomonas* genus, due to its high pathogenicity, but also other relevant genera, such as *Ralstonia*, *Agrobacterium*, *Xanthomonas*, *Erwinia*, *Xylella*, *Pectobacterium*, *Dickeya* [63], *Pantoea*, *Burkholderia*, *Acidovorax, Clavibacter*, and *Streptomyces* [4]. Table 1 compiles some of the most concerning bacterial phytopathogenic genera/species, as well as their main hosts.

*Xanthomonas* spp. is one of the most important phytopathogenic groups responsible for large economic losses, which led to their intensive study [3] (European and Mediterranean Plant Protection Organization-EPPO). These groups of species are host-specific, which can be related to the virulence mechanisms based on different secretion systems expressed [64]. The importance of these systems goes further than their pathogenicity. The T6SS and T4SS (type 6 and type 4 secretion systems) are present in almost all *Xanthomonas* species and are related to bacterial persistence in the environment, and protection against soil predators and bacterial competition [64]. *Agrobacterium* spp., has been responsible for the losses of economically relevant crops like vineyards and fruit orchards, namely, apple, pear, peach, cherry, grape, apricot, plum, and nuts trees, as well as vegetables and ornamental plants [4]. For the *Erwinia* genus, their easy spread, for example through insects, and their role in the detriment of fruit are the two main factors contributing to their position at the top of phytopathogenic bacteria [3].

Besides their intervention in *Erwinia* transmission, insects are also responsible for the dispersion of the aggressive citrus disease, Huanglongbing disease (citrus greening), caused by the Gram-negative “*Candidatus* Liberibacter”; this genus is responsible for the phloem vessels infections [118,119,120]. The term “*Candidatus*” refers to the impossibility to cultivate and grow this bacteria group in laboratory conditions [118], which is also difficult to detect as it is only possible through molecular methods such as PCR-based techniques [120,121]. There are three major species of this group mainly dispersed in Asia, Africa [122,123], and America [124,125] that can be transmitted by different vectors. In Europe, the dispersion of these species is also a concern since there are already reports of the presence of vectors in the Atlantic Coast of Portugal and northwest of Spain [118,126,127].

Also, among the species included in Table 1, *Pseudomonas syringae* should be highlighted, due to its high capacity to colonize plant tissues of a wide range of hosts [3], as well as it being the causative agent of bacterial canker in citrus and tomato, *Clavibacter michiganensis*, that appears as a problematic phytopathogenic bacterium [3,76].

The main goal of this work is to gather information regarding alternative methods to control diseases caused by phytopathogenic bacteria, focusing on macroalgae-derived extracts/compounds with antibacterial and/or priming potential. Complementary data regarding the studies focused on antibacterial activity against generalist pathogens and/or human bacterial pathogens are presented in the supplementary material (Tables S1–S6).

## 4. Phytopathogenic Antibacterial Potential of Seaweeds

Despite the considerable list of phytopathogenic bacteria reported in the section above (Table 1), the studies relying on macroalgae potential against this microbial group focus mainly on species belonging to the *Xanthomonas* and *Erwinia* genera. The pathogenicity of both genera leads to enormous losses of food every year, compromising the fruits, the root (in the case of Solanaceae organisms [128]), or the whole plant [129]. Besides these genera, some studies also address the susceptibility of *Ralstonia solaneacearum*, *P. syringae,* and *Agrobacterium tumefaciens* when exposed to macroalgae extracts (Table 2).

Table 2 is focused on research performed in in vitro conditions since it is the way to demonstrate and confirm the existence of antibacterial activity against microbial phytopathogens as their inhibition or suppression of disease symptoms in vivo experiments could also be a result of the triggering of plant defense mechanisms, belonging to priming events.

The complexity of metabolites produced by seaweeds and their respective bioactivities [41] are influenced by a myriad of factors, including the season and localization of algae [132,136], the species and life-cycle stage [137], the storage conditions and drying process [138], and the method and the solvent used to extract the compounds [128,129,139]. The refinery process of extraction is also important in the search for bioactivities [140]. In the works performed by Kumar and Rengasamy [134] and Rao and Parekh [141] the highest antibacterial activity against Gram-positive and Gram-negative bacteria was obtained after analyzing different fractions of the extracts, while the crude dry biomass did not exhibit the same potential.

**Figure 1:**
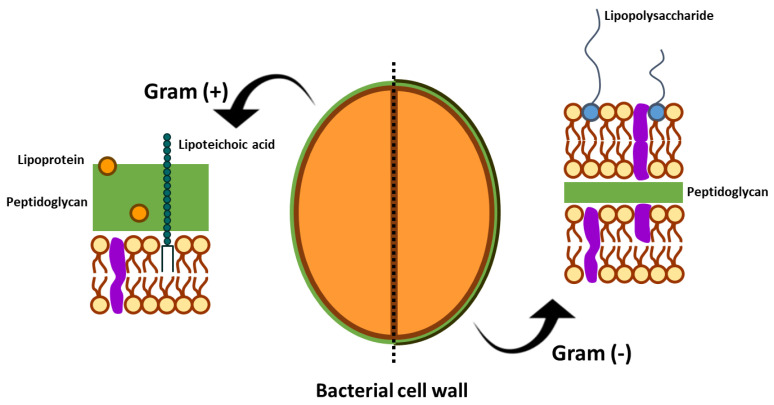
Differences in the structure of the cell wall of Gram-positive and Gram-negative bacteria. The schematic representation was adapted from the study of Akira et al. [142].

The nature of the solvent used can be a determinant factor in obtaining efficient antibacterial activity. The antibacterial potential of numerous aqueous extracts from red and brown macroalgae was observed against *S. aureus* [139], but to a lesser extent when compared with extracts obtained from different solvents, such as butanol and chloroform. However, the antibacterial activity of aqueous extracts from brown, red, and green macroalgae was completely absent when tested against *Erwinia chrysanthemi* [128]. This is possibly explained by the structural differences in the cell wall of Gram-negative and Gram-positive bacteria (Figure 1) [142]. *Erwinia chrysanthemi* (Gram-negative) [143] is a bacterium with an external bilayer, composed of lipopolysaccharides with a fine peptidoglycan layer in the middle, while *S. aureus* (Gram-positive) [144] only possesses one peptidoglycan layer, becoming more susceptible to the entrance of compounds and consequent cell disruption [142,145,146]. In addition, the main compound of the Gram-positive cell wall, peptidoglycan, is a more susceptible sugar to degradation than the complex composition of the Gram-negative cell wall, as already mentioned in other works [147]. Still, *E. chrysanthemi* was successfully inhibited by a dichloromethane extract from the same macroalgae used to produce the aqueous extracts [128] mentioned above. This situation unveils the clear interference of the solvents used to extract different bioactive compounds and consequently distinct activities and modes of action.

The use of chloroform to extract all kinds of fatty acids from red seaweeds has been reported as a suitable method to find antibacterial activity [139]. Lipophilic extracts and N-containing compounds, terpenes, and phenolic compounds present in aqueous/methanolic extracts can also be responsible for the active compounds possessing antibacterial activity present in the Rhodophyta group [130,148,149].

The potential of lipophilic compounds is not restricted to one algae group. Free fatty acids from brown algae are known to exhibit the relevant antibacterial activity against species associated with plant diseases [150]. The presence of phenolic acids (and their by-products) in N-butanol extracts of brown algae can contribute to the antibacterial activity presented [151] since this group includes a wide range of compounds with great antibacterial activity reported [46,146]. A compound of oil kind, a sulfonoglycolipid, was also isolated from *Sargassum wightii*, presenting antibacterial activity against the Gram-negative *X. oryzae* pv. *oryzae* [135]. Another algae compound that has also been highlighted is palmitic acid, which has been related with the antibacterial activity presented by algae extracts [135,152,153,154,155]. Using a mixture between non-polar solvents, methanol and toluene (3:1 *v*/*v*), Kumar and Rengasamy [129] also obtained lipophilic extracts from red and brown algae, possessing antibacterial activity against the phytopathogenic bacterium *X. oryzae* pv. *oryzae*. In addition, the work of Jiménez and colleagues [132] demonstrated the potential of polar compounds found in the ethanolic extracts from the brown alga *Lessonia trabeculate*. Villouta and Santelices, (1986) against two relevant tomato phytopathogenic bacteria, *Erwinia carotovora* (Jones, 1901) Bergey et al., (1923) and *P. syringae*.

Also, in Lakhdar’s study, a clear influence of the seaweed group was demonstrated, showing a greater predominance of antibacterial activity in brown and red algae when compared to the green algae group [128]. In the studies performed by Kumar and Rengasamy [134], macroalgae from the three different groups exhibited antibacterial activity against *Xanthomonas oryzae* pv. *oryzae*. The active substances may be related to polar compounds present in brown algae extracts and non-polar in red and green algae [129]. Specifically, this potential can be associated with the presence of phenolic compounds in the brown algae, which possess a high affinity for methanol [156], the solvent used to extract these bioactive compounds [129]. In addition, the antibacterial activity observed from the non-polar fractions obtained from red and green algae may be related to the presence of fatty acids [157] and unsaponifiable lipids [158]. The three classes of compounds referred have already demonstrated their antimicrobial activity against Gram-positive [139] and Gram-negative [141] bacteria.

Brown algae have a diverse range of compounds possessing a variety of promising bioactivities, among them is the antimicrobial activity against Gram-negative and Gram-positive phytopathogenic bacteria (Table 2). Extracts from brown algae showed a high inhibitory capacity against *R. solanacearum*, an important bacterium involved in the bacterial wilt disease of potato crops [7], and antibacterial activity against *Xanthomonas campestris* pv. *vesicatoria* [6] and *A. tumefaciens* [130], the causative agents of bacterial leaf spot and crown gall, respectively, which promote catastrophic losses in tomato cultures. Among the various algae and solvents tested, the methanol extracts obtained from *Cystoseira humilis* var. *myriophylloides* (then identified as *Cystoseira myriophylloides*) and *Laminaria digitata* contained the most effective compounds in the growth control of the crown gall causative agent [130]. This capacity was associated with the high abundance of phenolic compounds and pigments. As reported above, the phenolic compounds are one of the biggest and most complex groups abundant in macroalgae extracts that exhibited antibacterial activity against phytopathogenic bacteria. Some authors associated this potential with the presence of phenolic aromatic rings and hydroxyl groups promoting their binding with bacterial molecules, disturbing their cell viability [46,159]. In addition, various phenolic groups have been found in seaweed extracts, with antibacterial activity demonstrated through in vitro methodologies from a wide range of seaweed, including *Anthophycus longifolius* and *Gracilaria gracilis* (highlighting the abundancy of flavonoids), with activity against *Bacillus subtilis* [160,161]; *Caulerpa peltata*, *Caulerpa scalpelliformis*, *Sargassum aquifolium*, *Colpomenia peregrina*, *Ellisolandia elongata*, *Punctaria latifolia*, *Punctaria plantaginea*, *Scytosiphon lomentaria*, and *Zanardimia typus* with inhibition capacity against *Staphylococcus aureus* [139,162,163,164]; *Sarconema filiforme* against *Pseudomonas* sp. [165]; *Sargassum muticum* against *B. subtilis*, *Escherichia coli* and *S. aureus* [166]; *Sargassum tenerrimum* against *B. subtilis*, *E. coli, P. aeruginosa*, and *S. aureus* [167,168,169]; *Sargassum cristaefolium* against *E. coli* and *S. aureus* [164]; *Gracilaria corticata*, *S. wightii*, and *Ulva lactuca* against *P. aeruginosa* and *S. aureus* [170,171]. In cases of resistant bacteria, some macroalgae extracts have shown higher effectiveness when combined with artificial chemical products, such as antibiotics, demonstrating a positive synergistic activity between the antibiotics and the natural compounds present in the extracts. This situation was already observed in the study by Santos and co-workers, where the addiction of a *B. bifurcata* extract strongly enhanced the inhibitory potential of rifampicin and tetracycline against the antibiotic-resistant *E. coli* and *S. aureus* bacteria [150].

### Putative Mechanisms of Antibacterial Action

Some generalist mechanisms have been proposed to understand the action of seaweed compounds against bacteria. The main target of antibacterial compounds is the bacterial cell membrane, also mentioned above, but there are other bacterial components that are crucial to guarantee their survival, such as their inner molecules, focusing on proteins and nucleic acids. A relevant group of seaweed compounds strongly referred to in previous work are fatty acids. These compounds negatively influence the regular synthesis of lipids and other essential bacterial compounds responsible for the maintenance of microbial integrity [172]. An important component affected by abnormalities in fatty acid synthesis is the cell membrane, leading to the lysis of the cell [46]. Seaweed polysaccharides, including sulfated polysaccharides [173], also have been suggested as suitable compounds to eliminate bacteria, due to their capacity to bind to receptors in the cell surface, promoting the increase of permeability, protein damage, and interferences with bacterial DNA [174].

Although the complexity of the bacterial membrane varies, containing components with different affinities due to their polarity levels, there are important groups of compounds present in macroalgae that can easily bind with polar fractions, as well as non-polar portions of the membrane due to their amphipathic conformation, as with the case of terpenes and peptides [175,176,177]. In addition, a review from 2011, clearly exposes the great antibacterial potential of peptides as well as their mechanisms. Their interference with external proteins and lipids affects Gram-negative and Gram-positive bacteria, provoking disorders in the bi-layer membrane conformation [46,175].

Additionally, there is a group of polyphenols restricted to brown seaweed that confers to Phaeophyceae seaweed an advantage in antibacterial potential, as reported throughout previous work [146]. One of the most mentioned phenolic compounds, is phlorotannins, a chemical group strongly related to the antimicrobial capacity of seaweed extracts, due its affinity to linking with bacterial constituents (e.g., proteins) and the cell membrane, making it more susceptible to cell lysis. Another way of action is related to the suppression of expression of genes responsible for antibacterial resistance, such as that demonstrated in a study performed using a compound extracted from a brown seaweed *(Eisenia bicyclis)*, where the silencing of mecI, mecR1, and mecA gene expression turned the bacteria susceptible to methicillin [46,178].

## 5. Seaweed Potential as Plant-Priming Agent

The sessile characteristics of plants allow them to develop intrinsic mechanisms to avoid the negative effects caused by stresses of different natures. Focusing on the defense pathways developed by the plants to escape from microbial pathogen invasion, there is a set of processes combining genetic factors, biochemical processes, and the morphology of the plants [179,180] leading to the improvement of their robustness under external stresses. The resistance of plants against microbial invasion is called “cross protection” [181,182] and encompasses at least three different types of plant defenses: the systemic acquired resistance (SAR) and the induced systemic resistance (ISR) [182], both included in the systemically induced resistance of the plant [183], and the mycorrhiza-induced resistance (MIR) [180,184]. The SAR defense demands the general accumulation of the hormone salicylic acid (SA) in the plant, which can lead to the induction of the pathogenesis-related (PR) gene expression [180,182]. The ISR is a more specific mechanism to protect plants from microbial attack [182,185,186] and can be triggered by high concentrations of jasmonic acid (JA) and ethylene (ET) [179,183]. Mycorrhiza-induced resistance is a defense process that relies on the ancestral symbiotic relationships established between fungi and plant roots [187]. This symbiosis is beneficial to the plant since the fungus can provide nutrients and other compounds to promote plant growth, contributing also with their own defense mechanisms against a diversity of stresses [184,186].

Priming, a phenomenon of induced resistance in plants [180], is characterized by the triggering of defense pathways against biotic or abiotic stresses, allowing them to improve and augment the response in case of adverse conditions [180,188], and conferring more protection for future events and/or generations [180,188,189]. The result from previous contacts of the plant or prior generations with elicitors or “priming stimuli” [180] will promote the rapid activation of the defense mechanisms [190,191,192] and the ability to retain them through the next generations [190,191,192], helping to efficiently face similar threats in the future [188]. One of the primary defense responses elicited in plant cells is the massive production of reactive oxygen species (ROS), promoting small and localized events of oxidative bursts in plant tissues [193]. Currently, it is known that these toxic events have been important to establish the SAR mechanism and/or other priming mechanisms, in damaged plant cells and/or under stress conditions [180,194,195].

After the first exposure to determined stress, the plant stress memory acquires a modification called “stress imprint” [196]. This signal recognition is due to the storage information of the plant that mainly relies on epigenetic processes [188,196], defined as structural modifications promoted by changes in the gene expression, while the immutable nature of the nucleotide sequence is ensured [197,198,199].

**Figure 2:**
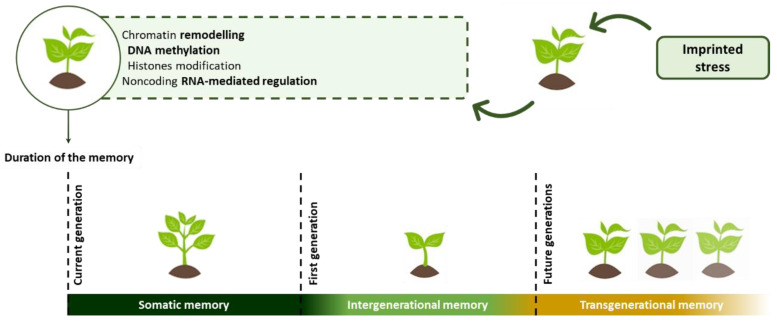
Different types of memories related to the duration of molecular modifications promoted by epigenetic events.

The occurred modifications in the plants can be categorized based on the duration of modifications promoted by the stress imprint, in somatic, intergenerational, and transgenerational memory (Figure 2) [200]. The somatic memory is associated with the term “mitotic stress memory”, due to its mitotic transmittance [201], and it is a short-term stress imprint limited to the current generation, preserving their capacity for reactivation along different stages of the life cycle [188]. Then, if the generation can transmit the stress imprint to the first generation, but this inheritable condition is lost to the second and next generations, it is termed intergenerational memory [200]. The longer-lasting modifications transmitted to future generations are defined as transgenerational memory and play a relevant role in the evolution of a species [188,202].

The epigenetic mechanisms involve a wide range of phenomena, such as chromatin remodeling, which possesses a central role in the stress responses [203], DNA cytosine methylation, nucleosome positioning, covalent modification of histones (posttranslational modification of histones) [204], and noncoding RNA-mediated regulation (RNA interference, RNAi). Such modifications are regulated by epigenetic regulators [188,197], which can be enzymes and other molecules, with the capacity to redefine the transcriptional mechanisms [188,203,204,205]. The action of the regulators is not “single-independent”, but it mainly occurs through their interaction, which is usually modulated through small non-coding RNAs [201], assuming a major relevance in the case of stress exposition [188,206]. The mechanism of response associated with stress memory is also dependent on the nature of the stress, its persistence and damage degree, and the plant species affected [188].

## 6. Seaweed Elicitors

Elicitors are an important part of the priming process since they mediate the plant response to stress, crucial in case of microbial attack [57]. These elicitors can be external compounds produced by other organisms, such as microbial biota, pathogens, predators [180], marine organisms [207], or stimulated by abiotic factors [190,191,192,198]. They are known to trigger mechanisms to avoid cellular and tissue damage in plants, reducing the disease symptoms.

Algae-derived compounds are known to be beneficial to plants [208,209], contributing to an improvement of plant nutritional profile [210] and defense against biotic and abiotic stresses [207,209]. Using the Scopus database, a compilation of the studies regarding the priming potential of macroalgae-derived extracts was performed and is detailed in Table 3.

There is a wide range of compounds capable of inducing systemic acquired resistance such as proteins, peptides, oligosaccharides, polysaccharides, fatty acids, glycoproteins, lipids [60,211,212,213], acid β-aminobutyric acid [57,214,215], among others. Some of these molecules are present in the composition of seaweed and have been proposed as “bio-elicitors” [216], highlighting the oligosaccharides, polysaccharides, peptides, proteins, and lipids [6].

The analysis of Table 3 shows a strong majority of extracts from brown algae associated with the capacity to initiate defense mechanisms in plants to fight bacterial invasions.

*Ascophyllum nodosum* is one of the most explored brown alga [217] and some of its compounds are already commercialized as biostimulants, due to their high potential to promote the healthy development and growth of plants [6,54,58]. Also, the extracts obtained from this alga present the capacity to exhibit phytoprotection in case of bacterial attack, leading to a reduction of disease symptoms. This capacity is not limited by the host plant and can be observed in different plant species [6]. Despite the fact that the mechanisms of “how” elicitors lead to the resistance against microbial invasions are not completely described [179], it is known that the resistant plants mainly exhibit high levels of phenolic compounds, such as tannins and flavonoids [6,218].

The studies described below focus their research on specific compounds or molecules in an attempt to understand eliciting behavior in plants since knowledge of this field is still scarce. Small oligogalacturonides are a molecular group present in brown algae and their eliciting activity has been reported through different plant groups [219,220,221,222,223,224]. One of the reasons for that is associated with the β-1,3 linkages of the molecules that can be recognized as defense signals by plants [225]. One of the oligogalacturonides most studied, and also present in *A. nodosum*, is laminarin [225,226,227]. This linear β-1,3 glucan can strongly trigger the activity of the PR proteins [225], phenylalanine ammonium lyase (PAL), and lipoxygenase [58,225], and promote the up-regulation of caffeic acid and *O*-methyltransferase (both involved in the regeneration process due to their inclusion in the lignin synthesis [228]). The influence of laminarin in SA accumulation on plants is controversial. Some studies observed an increase of SA in plants when stimulated with that compound [47,225], but other studies observed the inhibition of SA accumulation. SA is derived from the phenylpropanoid pathway and some studies established a correlation between SA accumulation and the increase in phenylalanine ammonia-lyase (PAL), a defense enzyme [229] that is a precursor of SA [230]. This correlation was also observed in the study by Klarzynski [225], where the accumulation of SA was reported, but no direct correlation was proved. This lack of strictness may be expected, once PAL is also a precursor for other molecules, such as the intermediates to the lignin formation [230]. More detailed studies, including the chemical characterization of the present compounds, are crucial to understanding the molecular pathways that promote or suppress SA accumulation.

**Table 3:** Compilation of studies available on Scopus database approaching the priming potential/activity of seaweed extracts against bacterial phytopathogens (*—Seaweed extract with bioactive compounds concentrated; ^A^—Purified seaweed compound used in the study).

Species	Seaweed	Extract/Solvent	References
*Agrobacterium tumefaciens*	*Fucus spiralis*	Aqueous extract	[130]
	*Cystoseira myriophylloides*	Aqueous extract	[130]
*Erwinia carotovora* subsp. *carotovora*	*Laminaria digitata*	Purified laminarin ^A^	[225]
*Pseudomonas aeruginosa*	*Ascophyllum nodosum*	-	[231]
	*Ascophyllum nodosum*	Stella Maris^®^	[227]
*Pseudomonas syringae*	*Ascophyllum nodosum*	Stella Maris^®^	[227]
*Pseudomonas syringae* pv. *tabaci*	*Cystoseira myriophylloides*	Aqueous extract	[71]
	*Fucus spiralis*	Aqueous extract	[71]
	*Laminaria digitata*	Aqueous extract	[71]
*Pseudomonas syringae* pv. *tomato*	*Ascophyllum nodosum*	Aqueous extract	[179]
	*Ascophyllum nodosum*	Chloroform extract	[179]
	*Ascophyllum nodosum*	Ethyl acetate	[179]
	*Kappaphycus alvarezii*	Aqueous extract	[232]
*Staphylococcus aureus*	*Ascophyllum nodosum*	Essential oils	[231]
*Xanthomonas campestris*	*Ascophyllum nodosum*	Stella Maris^®^	[227]
*Xanthomonas campestris* pv. *malvacearum*	*Sargassum wightii*	Aqueous extract (Dravya)	[233]
*Xanthomonas campestris* pv *vesicatoria*	*Ascophyllum nodosum*	Alkaline extract (commercial product)	[6,56]
	*Acanthophora spicifera*	Alkaline extract	[234]
	*Gelidium serrulatum*	Alkaline extract	[235]
	*Sargassum filipendula*	Alkaline extract	[235]
	*Sargassum vulgare*	Alkaline extract	[234]
	*Ulva lactuca*	Alkaline extract	[235]
*Xanthomonas oryzae* pv *oryzae*	*Kappaphycus alvarezii*	Aqueous extraction *	[232]

Alginate, one of the most commercialized phycocolloids, is the most abundant component present in brown algae, being part of the cell walls and intercellular matrix [236]. Its extended use by the food industry has demonstrated the safety of its consumption, turning this compound attractive to the agricultural field [216]. Alginate and alginate-derivative compounds extracted from brown algae demonstrated their effectiveness at activating the defense mechanisms of plants [237,238], and consequently conferring resistance against microbial phytopathogens [229,239]. The depolymerization of alginate originates from a digested agent, the oligo-alginate, that possesses eliciting activity and other agricultural benefits already reported in a wide range of studies. Zhang et al. demonstrated the capacity of alginate oligosaccharide to increase the expression of resistance genes and SA content in *A. thaliana*, to protect the plant against the *P. syringae* pv. tomato infection [240]. In another work, the degraded alginate proved to be beneficial for plant growth, in addition to the protection conferred to tobacco plants from microbial phytopathogens, proposing a hypothetical connection between these two plant mechanisms [241]. This hypothesis is based on the binding of molecules of bacterial presence recognition by the plant host, denominated by MAMPs (microbial-associated molecular patterns), to receptors that also can interact with BAK1 (BRI1-associated receptor kinase, coreceptor in plasma membrane), proving a dependence between these two phenomena that are apparently independent [242,243]. Then, the existence of receptors for oligo-alginates in the plasma membrane is proposed to somehow interact with the coreceptor BAK1, activating both the plant stimulation growth and defense response against bacterial invasion [241,244]. More specifically, an oligo-alginate of D-mannuronic has been associated with the induction of PAL activity, involved in the SA-dependent defense response [245,246].

A study from 2011 studied the mechanism of *A. nodosum* in a model plant, *A. thaliana* [179]. Using plants with different mutant genes related to the accumulation of SA or involved in the mediation of JA response, it was observed that no differences in the susceptibility of the plant to *Pseudomonas syringae* pv. *tomato* was found. However, the susceptibility to this phytopathogen increased in plants with the mutant gene *jar1* [179], attributed to the inability to create JA-Ile bonds [247] and consequently the failure to protect the plant against the pathogen. The mechanism proposed is based on the binding of algae sterols (usually, present in brown algae, including *A. nodosum*) to nonspecific lipid transfer proteins (nsLPTs), which are proteins that can transport lipids due to the presence of hydrophobic cavities present in a wide range of plants, as *A. thaliana* [248]. The importance of lipid molecules, such as jasmonic acid or oxylipins, to promote the expression of nsLTP genes in plants [249] was already defined as crucial to activate their defense pathways against microbial pathogens [250].

Also, another macroalgae group of relevance regarding the induction of defense pathways in plants is Rhodophyta. The eliciting activity of red algae in plants against bacterial phytopathogens was demonstrated in studies performed by Ramkissoon [235], and more recently by Ali et al. [234]. The alkaline extracts of *A. spicifera* and *G. serrulatum* were able to reduce the damage and presence of *Xanthomonas campestris* pv. *vesicatoria* in sweet pepper [234] and tomato plants [235]. In this study, high values of defense enzymes, phenolic compounds, and the upregulation of gene expression related to plant growth hormones were found. This eliciting potential has been assigned to the wide range of carrageenans usually present in red algae [211,216,251,252]. This group of sulfate polysaccharides includes a high degree of variability, and the position and number of sulfate ester groups determines the subgroup of these chemical compounds, λ-carrageenan being the one that contains a higher sulfate content (41%), followed by ι-carrageenan (33%) and κ-carrageenan (20%) [253,254]. In another work, Mercier and colleagues demonstrated the high efficiency of this family of sulfate linear galactan to promote the signaling cascade of plant defense [57,215]. In addition to the relevance of the presence of sulfated groups to promote their solubility in water [253], the number and position of ester sulfate groups of the carrageenans can also be a determinant factor to define, which of the defense mechanisms is activated [234]. Usually, the most sulfated carrageenans have been related as promoters to induce the ISR response [215], while the less sulfated ones have been pointed out as the responsible agents for SA signaling activation [234,235]. This was demonstrated by Sangha et al. [255], who reported a higher expression of genes associated with the JA signaling (*AOS*, *PDF1.2*, and *PR3*), in plants elicited by λ-carrageenan. However, the same study pointed out the relevance of the application of the right carrageenans type in the defense mechanisms of the plant: the use of less sulfated carrageenan, ι-carrageenan, enhanced the susceptibility of *A*. *thaliana* to the necrotrophic fungal pathogen [255]. An unusual behavior of carrageenans was exhibited in a later study by Sangh and co-workers [256], in which a higher activity of ι-carrageenan to induce the expression of genes associated with JA (PDF1.2) and SA (PR1) defense pathways was demonstrated, while the κ-carrageenan only promoted the expression of PDF1.2 in a reduced extent, and the λ-carrageenan did not affect the expression of the defense pathways. The controversial results from the above assays indicated that the association of sulfation level with the eliciting pathways of plants is not linear, which can denote that the sulfation level of these polysaccharides is only one important characteristic among other parameters involved in the expression of plant defense genes when interacting with carrageenans [255].

Green algae are a less-reported group with agricultural applications. However, the few studies existing also reported some eliciting activity from the polysaccharides usually present in the Chlorophyta group [257]. Based on that, El Modafar and co-workers searched for the potential of glucuronan and ulvan, a non-sulfate homopolymer and a sulfated polysaccharide, respectively, which are the main components of the cell wall of *Ulva lactuca* [258,259]. The sulfate homopolymer, ulvan, exhibited high eliciting activity in tomato seedlings, while glucuronan (glucuronic acids β-(1,4)) did not significantly affect the PAL activity, a precursor for the SA pathway [251]. Surprisingly, the sulfate compound, ulvan, which also is one of the mainly water-soluble polysaccharides [260], demonstrated stronger eliciting activity when compared to other polysaccharides (carrageenan, laminarin, and alginate). It is important to highlight that this eliciting activity can be related to the sulfate portion of the compound. The desulfation of ulvan led to the inability of this compound to promote PAL activity [251]. However, some studies are controversial regarding the defense responses triggered in plants. A study by Ramkissoon et al. [235] demonstrated that a *U. lactuca* extract promoted JA/ET signaling in tomato plants, defending that the presence of the sulfate polysaccharide ulvan is responsible for that.

Considering the complexity of the plant defense mechanisms, as well as all the factors that can affect the chemical composition of an extract, it is possible that slight modifications on the compounds may trigger such different responses in plants. The behavior of a diverse group of sulfate oligosaccharides based on just a few studies is not enough to define a generalist pathway, as was suggested in the past when it was proposed that the sulfated oligosaccharides were able to activate JA/ET signaling pathways in plants [235]. In the same way that there are some studies supporting this hypothesis [235,261], there are also studies demonstrating a positive relationship between sulfated oligosaccharides and the induction of genes related to SA plant response [251,262].

## 7. Conclusions

The constant increase in bacterial phytopathogens and their paramount impacts on agricultural production have boosted the search for effective methodologies while ensuring the security of the environment. From all the studies analyzed, the search for antibacterial or priming activity in extracts obtained from seaweeds seems to be one promising and suitable method to address the current demands of society for effective, green, and sustainable tools.

The analyses of studies reporting activity against bacterial phytopathogens demonstrate that brown seaweed is the group with the highest success in this area. This may be associated with the high diversity of their compounds; phenolic compounds being mostly associated with the antibacterial activity and the sulfated groups associated with the priming activity. The mechanisms underlying these processes are still not fully understood. The integration of data from different studies regarding the interaction between the compounds and the plant is crucial to fully deciphering the mechanism and also the means to enable the integration of different compounds into the same treatment for enhanced productivity and a wider array of protection.

Thus, despite all the work performed, this compilation demonstrates an urgent demand for more detailed studies, to obtain more accurate responses underlying the antibacterial activity and/or priming potential of seaweed extracts to aid the development of marine-based solutions from the sea to the farm.

## Figures and Tables

**Table 1:** Summary of the most economically damaging phytopathogenic bacteria species (*—Species or genera included in the top ranking defined by Kannan and colleagues [4] as the most relevant bacterial phytopathogens).

Genera	Species	Hosts	References
Agrobacterium *	*Agrobacterium tumefaciens* * (syn. *Rhizobium radiobacter*)	Wide range of agriculturally and economically relevant species, including vines, shade and fruit trees, woody ornamental plants, herbaceous perennials, and other monocots and (mainly) dicotyledonous (host list undefined).	[3,61,64,65,66,67,68,69,70,71,72,73,74,75]
*Clavibacter*	*Clavibacter michiganensis* subsp. *michiganensis*	Tomato	[3,76,77]
Corynebacterium *	*Corynebacterium fascians*	Wide range of ornamental and consumable vegetal species	[72,78]
	*Corynebacterium michiganense*	Solanaceous host plants	[72,79,80,81]
	*Corynebacterium sepedonicum*	Potato	[72,82,83]
*Curtobacterium*	*Curtobacterium flaccumfaciens* pv. *flaccumfaciens*	Bean	[72,84]
	*Curtobacterium flaccumfaciens* pv. *poinsettiae*	Poinsettia	[72,84]
*Dickeya*	*Dickeya dadantii* *	Wide range of economically relevant plant species, highlighting the tropical and subtropical species	[4,63,85]
	*Dickeya solani* *	Potato	[4,63]
*Erwinia* *	*Erwinia amylovora* *	Fruits of diverse hosts (pear, apple), Rosaceae family	[3,4,64,65,66,67,68,69,70,71,72]
*Pectobacterium*	*Pectobacterium atrosepticum* *	Potato	[4,63]
	*Pectobacterium carotovorum* *	Diverse crop species	[4,72]
*Pseudomonas* *	*Pseudomonas aeruginosa*	Tobacco, soybean, bean, cucumber, tomato, and other crops	[3,62,64,65,66,67,68,69,70,71]
	*Pseudomonas syringae* pv. *lachrymans*	Cucumber	[72,86,87,88,89,90]
	*Pseudomonas marginalis*	Wide range of vegetables (such as tomato, parsnip) and ornamental plants (e.g., *Zantedeschia* spp.)	[72,91,92,93,94,95]
	*Pseudomonas syringae* pv. *morsprunorum*	Stone fruit of *Prunus* species (cherries, plum, apricots, peaches)	[72,92,96,97,98]
	*Pseudomonas savastanoi* pv. *sacastanoi* *	Oleaceae family plants and oleander (*Nerium oleander*)	[72,92,99,100,101]
	*Pseudomonas syringae* *	Prunus species	[3,4,72]
	*Pseudomonas syringae* pv. *tomato* *	Tomato	[72,102,103]
	Ralstonia solanacearum* (syn. *Pseudomonas solanacearum*)	Wide range of species including solanaceous plants, weeds, crops, shrubs, and trees	[3,4,61,72,76,104]
*Staphylococcus*	*Staphylococcus aureus*	*Arabidopsis thaliana*	[105,106]
*Xanthomonas* *	*Xanthomonas axonopodis* *	Orange, cassava, tomato, pepper, crucifers, cotton, rice, beans, grapes, and others	[3,4,64,65,66,67,68,69,70,71,76]
	*Xanthomonas campestris* *	Cruciferous plants (including species economically important)	[4,72,107]
	*Xanthomonas citri* subsp. *citri*	Citrus species (including the economical varieties)	[61,108,109]
	*Xanthomonas euvesicatoria*	Solanaceous species	[61,110,111,112]
	*Xanthomonas oryzae* pv. Oryzae *	Rice species	[4,113,114]
	*Xanthomonas phaseoli*	Common bean	[72,115]
*Xylella*	*Xylella fastidiosa* *	Olive, citrus species	[3,4,116,117]
‘*Candidatus* Liberibacter’ *	*-*	Citrus species	[118]

**Table 2:** Species of seaweed demonstrating antibacterial activity against relevant phytopathogenic bacteria (compilation of the available information in Scopus until 26 October 2022). Detailed information regarding the extraction methodology of the compounds and the techniques used to evaluate the antibacterial activity can be found in supplementary material (Tables S1–S6).

Species	Macroalgae Source	Antibacterial Activity Test	References
*Agrobacterium tumefaciens*	*Cystoseira humilis* var. *myriophylloides*	Agar diffusion technique	[130]
	*Laminaria digitata*		
*Bacillus subtilis*	*Cladophora glomerata*	Disc diffusion technique	[131]
	*Chara vulgaris*		
	*Spirogyra crassal*		
*Erwinia carotovora*	*Lessonia trabeculata*	Liquid-dilution method	[132]
	*Ulva lactuca*	Agar diffusion technique	[133]
*Erwinia chrysanthemi*	*Bifurcaria bifurcata*	Agar diffusion technique	[128]
	*Codium decorticatum*		
	*Cystoseira humilis* var. *myriophylloides*		
	*Ellisolandia elongata*		
	*Ericaria selaginoides*		
	*Fucus spiralis*		
	*Gelidium corneum*		
	*Gelidium sp*		
	*Gracilaria cervicornis*		
	*Gymnogongrus crenulatus*		
	*Halopitys incurva*		
	*Laminaria digitata*		
	*Osmundea pinnatifida*		
	*Plocamium cartilagineum*		
	*Sargassum vulgare*		
	*Ulva intestinalis*		
	*Ulva* sp.		
*Pseudomonas syringae*	*Lessonia trabeculata*	Liquid-dilution method	[132]
	*Macrocystis pyrifera*		
	*Sargassum wightii*	Disc diffusion technique	[133]
*Ralstonia solaneacearum*	Brown seaweed	Field studies	[7]
	*Cladophora glomerata*	Disc diffusion technique	[131]
	*Chara vulgaris*		
	*Spirogyra crassal*		
*Staphylococcus aureus*	*Cladophora glomerata*	Disc diffusion technique	[131]
	*Chara vulgaris*		
	*Spirogyra crassal*		
*Xanthomonas campestris*	*Cladophora glomerata*	Disc diffusion technique	[131]
	*Chara vulgaris*		
	*Spirogyra crassal*		
	*Ulva lactuca*	Agar diffusion assay	[133]
*Xanthomonas oryzae* pv. *oryzae*	*Chnoospora minima*	Agar diffusion assay	[129,134,135]
	*Gracilaria blodgettii*		
	*Gracilaria edulis*		
	*Hypnea musciformis*		
	*Hypnea valentiae*		
	*Padina boergesenii*		
	*Spyridia hypnoides*		
	*Turbinaria conoides*		
	*Ulva flexuosa*		
	*Ulva lactuca*		
	*Sargassum wightii*		

## Data Availability

Not applicable.

## Supplementary Materials

The following supporting information can be downloaded at: https://www.mdpi.com/article/10.3390/md21010023/s1, Table S1: Detailed information of antibacterial activity reported from disc/well diffusion technique; Table S2: Detailed information of antibacterial activity reported from disc diffusion method modified (bacterial-agar medium); Table S3: Detailed information of antibacterial activity reported from liquid-dilution method; Table S4: Detailed information of antibacterial activity from microdilution method; Table S5: Detailed information of antibacterial activity reported by a spectrophotometric method; Table S6: Detailed information about antibacterial activity from field studies [263,264,265,266,267,268,269,270,271,272,273,274,275,276,277,278,279,280,281,282,283,284,285,286,287,288,289,290,291,292,293,294,295,296].
