# Supplementary material for: Seaweed as a Natural Source against Phytopathogenic Bacteria
Source: Mar Drugs. 2022 Dec 28;21(1):23. doi: 10.3390/md21010023 (PMC9867177; doi:10.3390/md21010023)
Supplement: Supplementary file 1 [file marinedrugs-21-00023-s001.zip › marinedrugs-2098496-supplementary.pdf]

Supplementary material

Antibacterial activity/potential of macroalgae extracts against phytopathogenic bacteria and/or related to plant pathogenicity

In the following pages, it will be presented all the information about macroalgae extracts/dry powder with the potential to inhibit phytopathogenic bacteria or related species. The data is organized in sections, and it was organized by methodologies.

Note: All the data information in the supplementary material I were retrieved from the SCOPUS database using the following search: "**Antibacteria* AND (Plant* OR crop* OR agricultur* OR veget* OR phytopatho*) AND (Macroalga* OR seaweed)".**

Table S1. Detailed information of antibacterial activity reported form disc/well diffusion technique.

Disc/well diffusion technique									
**Phytopathogenic bacteria**	**Macroalgae source**	**Extraction solvent**	**Collection conditions**	**Composition of the extract**	**Possible composition (comparative information)**	**Extract concentration/ volume (extract)**	**Inhibition halo (diameter_mm) /(%)**	**Notes**	**Reference**
Agrobacterium tumefaciens	*Cystoseira humilis* var. *myriophylloides*	Methanol	-	-	-	Soaked in ASE at 1.5%	>30 mm	-	[1]
	Laminaria digitata	Methanol	-	-	-	Soaked in ASE at 1.5%	>30 mm	-	[1]
Bacillus subtilis	*Aglaothamnion sepositum*	Dry powder	Spring	-	-	Disc thallus	1,5 mm	-	[2]
		Dry powder	Summer	-	-	Disc thallus	1 mm	-	[2]
		Dry powder	Autumn	-	-	Disc thallus	1,5 mm	-	[2]
		Dry powder	Winter	-	-	Disc thallus	1 mm	-	[2]
	Alaria esculenta	Dry powder	Summer	-	-	Disc thallus	2 mm	-	[2]
		Dry powder	Autumn	-	-	Disc thallus	3 mm	-	[2]
	Anthophycus longifolius	Benzene	-	Proteins and amino acids, phenolic compounds, alkaloids, carbohydrates	-	"Standard concentration"	16.23±0.145 mm	Zones greater than 10 mm are considered positive results.	[3]
		Acetic acid	-	Proteins and amino acids, phenolic compounds, carbohydrates, alkaloids	-	"Standard concentration"	17.5±0.289 mm	Zones greater than 10 mm are considered positive results.	[3]
		Hexane	-	Proteins and amino acids, phenolic compounds, carbohydrates, alkaloids	-	"Standard concentration"	15.43±0.296 mm	Zones greater than 10 mm are considered positive results.	[3]
		DMSO	-	Proteins and amino acids, phenolic compounds,	-	"Standard concentration"	20.5±0.500 mm	Zones greater than 10 mm are considered positive results.	[3]

				alkaloids, sugar/glucosides					
		Diethyl ether	-	Proteins and amino acid, phenolic compounds, alkaloids, sugar/glucosides	-	"Standard concentration"	16.5±0.289 mm	Zones greater than 10 mm are considered positive results.	[3]
Bacillus subtilis	*Anthophycus longifolius*	Chloroform	-	Proteins and amino acids, glycosides, phenolic compounds, alkaloids, sugar/glucosides	-	"Standard concentration"	18.16±0.166 mm	Zones greater than 10 mm are considered positive results.	[3]
	Antithamnion cruciatum	Dry powder	Spring	-	-	Disc thallus	2,5 mm	-	[2]
		Dry powder	Summer	-	-	Disc thallus	1 mm	-	[2]
	Asparagopsis armata	Dry powder	Summer	-	-	Disc thallus	11 mm	-	[2]
	Avrainvillea nigricans	Ethyl acetate	-	-	-	100 µL	0.36	Data of inhibition not specified	[4]
	Bifurcaria bifurcata	Dry powder	Spring	-	-	Disc thallus	1,5 mm	-	[2]
		Dry powder	Summer	-	-	Disc thallus	2 mm	-	[2]
		Dry powder	Autumn	-	-	Disc thallus	1,5 mm	-	[2]
	Bonnemaisonia asparagoides	Dry powder	Summer	-	-	Disc thallus	13 mm	-	[2]
	Bonnemaisonia hamifera	Dry powder	Summer	-	-	Disc thallus	15 mm	-	[2]
	Bostrychia scorpioides	Dry powder	Spring	-	-	Disc thallus	2 mm	-	[2]
		Dry powder	Summer	-	-	Disc thallus	0,5 mm	-	[2]
		Dry powder	Autumn	-	-	Disc thallus	1 mm	-	[2]
		Dry powder	Winter	-	-	Disc thallus	1,5 mm	-	[2]
	Bryopsis plumosa	Dry powder	Spring	-	-	Disc thallus	1,5 mm	-	[2]
		Dry powder	Summer	-	-	Disc thallus	0,5 mm	-	[2]
	Alsidium triquetrum	Ethanol	-	-	-	20 µL	7.7±1.1 mm	-	[5]
		Chloroform	-	-	-	20 µL	9.3±0.6 mm	-	[5]
		Dry powder	Spring	-	-	Disc thallus	3 mm	-	[2]

	Callithamnion tetragonum	Dry powder	Summer	-	-	Disc thallus	3 mm	-	[2]
		Dry powder	Autumn	-	-	Disc thallus	3 mm	-	[2]
Bacillus subtilis	*Callithamnion tetragonum*	Dry powder	Winter	-	-	Disc thallus	2 mm	-	[2]
	Callithamnion tetricum	Dry powder	Spring	-	-	Disc thallus	3,5 mm	-	[2]
		Dry powder	Summer	-	-	Disc thallus	4 mm	-	[2]
		Dry powder	Autumn	-	-	Disc thallus	4 mm	-	[2]
		Dry powder	Winter	-	-	Disc thallus	2,5 mm	-	[2]
	Callocolax neglectus	Dry powder	Summer	-	-	Disc thallus	1 mm	-	[2]
		Dry powder	Autumn	-	-	Disc thallus	0,5 mm	-	[2]
	Carradoriella elongata	Dry powder	Spring	-	-	Disc thallus	12 mm	-	[2]
		Dry powder	Summer	-	-	Disc thallus	12,5 mm	-	[2]
		Dry powder	Autumn	-	-	Disc thallus	12 mm	-	[2]
		Dry powder	Winter	-	-	Disc thallus	10 mm	-	[2]
	Caulerpa ashmeadii	Ethanol	-	-	-	20 µL	10.3±0.6 mm	-	[5]
		Chloroform	-	-	-	20 µL	11.3±0.6 mm	-	[5]
	Caulerpa cupressoides	Ethanol	-	-	-	20 µL	11.0±1.0 mm	-	[5]
	Caulerpa mexicana	Ethanol	-	-	-	20 µL	9.3±0.6 mm	-	[5]
		Chloroform	-	-	-	20 µL	7.0±0.0 mm	-	[5]
	Caulerpa paspaloides	Ethanol	-	-	-	20 µL	8.6±0.6 mm	-	[5]
		Chloroform	-	-	-	20 µL	8.3±0.6 mm	-	[5]
	Caulerpa prolifera	Ethanol	-	-	-	20 µL	9.6±0.6 mm	-	[5]
		Chloroform	-	-	-	20 µL	10.0±0.0 mm	-	[5]
	Caulerpa racemosa	Ethanol	-	-	-	20 µL	7.3±0.6 mm	-	[5]
		Chloroform	-	-	-	20 µL	7.0±0.0 mm	-	[5]
	Caulerpa taxifolia	Acetone	Septembre Station 1 Distal portion	-	-	100 µL (200 mg of dry weight)	0-5 mm	Graphical data	[6]
		Acetone	Septembre Station 1 Proximal portion	-	-	100 µL (200 mg of dry weight)	0-5 mm	Graphical data	[6]
		Acetone	Septembre Station 2 Distal portion	-	-	100 µL (200 mg of dry weight)	10-15 mm	Graphical data	[6]
		Acetone	Septembre Station 2	-	-	100 µL (200 mg of dry weight)	10 mm	Graphical data	[6]

			Proximal portion						
Bacillus subtilis	*Caulerpa taxifolia*	Acetone	April<br>Station 1<br>Distal portion	-	-	100 µL (200 mg of dry weight)	0-5 mm	Graphical data	[6]
		Acetone	April<br>Station 2<br>Distal portion	-	-	100 µL (200 mg of dry weight)	10-15 mm	Graphical data	[6]
		Acetone	April<br>Station 2<br>Proximal portion	-	-	100 µL (200 mg of dry weight)	10-15 mm	Graphical data	[6]
		Ethanol	Septembre<br>Station 1<br>Distal portion	-	-	100 µL (200 mg of dry weight)	0-5 mm	Graphical data	[6]
		Ethanol	Septembre<br>Station 1<br>Proximal portion	-	-	100 µL (200 mg of dry weight)	0-5 mm	Graphical data	[6]
		Ethanol	Septembre<br>Station 2<br>Distal portion	-	-	100 µL (200 mg of dry weight)	5-10 mm	Graphical data	[6]
		Ethanol	Septembre<br>Station 2<br>Proximal portion	-	-	100 µL (200 mg of dry weight)	5-10 mm	Graphical data	[6]
		Ethanol	April<br>Station 1<br>Distal portion	-	-	100 µL (200 mg of dry weight)	0-5 mm	Graphical data	[6]
		Ethanol	April<br>Station 1<br>Proximal portion	-	-	100 µL (200 mg of dry weight)	5 mm	Graphical data	[6]
		Ethanol	April<br>Station 2<br>Distal portion	-	-	100 µL (200 mg of dry weight)	10-15 mm	Graphical data	[6]
		Ethanol	April<br>Station 2<br>Proximal portion	-	-	100 µL (200 mg of dry weight)	5-10 mm	Graphical data	[6]
	Ceramium nitens	Ethanol	-	-	-	20 µL	10.0±1.7 mm	-	[5]
		Chloroform	-	-	-	20 µL	22.3±2.1 mm	-	[5]
	Chaetomorpha antennina	Petroleum ether	-	-	-	100 µg/mL (dissolved in DMSO)	6 ± 0.6 mm	-	[7]

Bacillus subtilis	*Chaetomorpha antennina*	Petroleum ether	-	-	-	200 µg/mL (dissolved in DMSO)	5 ± 0.8 mm	-	[7]
		Petroleum ether	-	-	-	300 µg/mL (dissolved in DMSO)	7.33 ± 0.5 mm	-	[7]
		Petroleum ether	-	-	-	400 µg/mL (dissolved in DMSO)	11.66 ± 0.5 mm	-	[7]
		Petroleum ether	-	-	-	500 µg/mL (dissolved in DMSO)	12.5 ± 0.8 mm	-	[7]
		Acetone	Septembre<br>Station 1<br>Distal portion	-	-	100 µL (200 mg of dry weight)	15-20 mm	Graphical data	[6]
		Acetone	Septembre<br>Station 1<br>Proximal portion	-	-	100 µL (200 mg of dry weight)	15-20 mm	Graphical data	[6]
		Acetone	Septembre<br>Station 2<br>Distal portion	-	-	100 µL (200 mg of dry weight)	10-15 mm	Graphical data	[6]
		Acetone	Septembre<br>Station 2<br>Proximal portion	-	-	100 µL (200 mg of dry weight)	15 mm	Graphical data	[6]
		Acetone	December<br>Station 2<br>Distal portion		-	100 µL (200 mg of dry weight)	10-15 mm	Graphical data	[6]
		Acetone	December<br>Station 1<br>Proximal portion	-	-	100 µL (200 mg of dry weight)	5-10 mm	Graphical data	[6]
		Acetone	April<br>Station 1<br>Distal portion	-	-	100 µL (200 mg of dry weight)	5-10 mm	Graphical data	[6]
		Acetone	April<br>Station 1<br>Proximal portion	-	-	100 µL (200 mg of dry weight)	10 mm	Graphical data	[6]
		Acetone	April<br>Station 2<br>Distal portion	-	-	100 µL (200 mg of dry weight)	15-20 mm	Graphical data	[6]
		Acetone	April<br>Station 2	-	-	100 µL (200 mg of dry weight)	10 mm	Graphical data	[6]

			Proximal portion						
Bacillus subtilis	*Chaetomorpha antennina*	Ethanol	Septembre Station 1 Distal portion	-	-	100 µL (200 mg of dry weight)	15-20 mm	Graphical data	[6]
		Ethanol	Septembre Station 1 Proximal portion	-	-	100 µL (200 mg of dry weight)	20-25 mm	Graphical data	[6]
		Ethanol	Septembre Station 2 Distal portion	-	-	100 µL (200 mg of dry weight)	5 mm	Graphical data	[6]
		Ethanol	Septembre Station 2 Proximal portion	-	-	100 µL (200 mg of dry weight)	5-10 mm	Graphical data	[6]
		Ethanol	December Station 1 Distal portion	-	-	100 µL (200 mg of dry weight)	20-25 mm	Graphical data	[6]
		Ethanol	December Station 1 Proximal portion	-	-	100 µL (200 mg of dry weight)	10-15 mm	Graphical data	[6]
		Ethanol	April Station 1 Distal portion	-	-	100 µL (200 mg of dry weight)	5-10 mm	Graphical data	[6]
		Ethanol	April Station 1 Proximal portion	-	-	100 µL (200 mg of dry weight)	5-10 mm	Graphical data	[6]
		Ethanol	April Station 2 Distal portion	-	-	100 µL (200 mg of dry weight)	10 mm	Graphical data	[6]
		Ethanol	April Station 2 Proximal portion	-	-	100 µL (200 mg of dry weight)	10 mm	Graphical data	[6]
	Chaetomorpha linum	Ethyl acetate	-	-	-	1.25 mg/disc	9.33±0.58 mm	-	[8]
		Ethyl acetate	-	-	-	2.5 mg/disc	10.00±0.00 mm	-	[8]
		Ethyl acetate	-	-	-	5 mg/disc	11.00±0.00 mm	-	[8]
		Acetone	-	-	-	1.25 mg/disc	10.67±0.58 mm	-	[8]
		Acetone	-	-	-	2.5 mg/disc	12.67±0.58 mm	-	[8]
		Acetone	-	-	-	5 mg/disc	15.67±0.58 mm	-	[8]
	Chondria dasyphylla	Spring	-	-	-	Disc thallus	3 mm	-	[2]
		Summer	-	-	-	Disc thallus	0,5 mm	-	[2]

Bacillus subtilis	*Chondria dasyphylla*	Autumn	-	-	-	Disc thallus	1,5 mm	-	[2]
		Winter	-	-	-	Disc thallus	1,5 mm	-	[2]
	Chondrus crispus	Spring	-	-	-	Disc thallus	11,5 mm	-	[2]
		Summer	-	-	-	Disc thallus	12 mm	-	[2]
		Autumn	-	-	-	Disc thallus	12 mm	-	[2]
		Winter	-	-	-	Disc thallus	17 mm	-	[2]
	Chordaria flagelliformis	Summer	-	-	-	Disc thallus	2,5 mm	-	[2]
		Autumn	-	-	-	Disc thallus	2 mm	-	[2]
	Cladophora vagabunda	Acetone	Septembre Station 1 Distal portion	-	-	100 µL (200 mg of dry weight)	10-15 mm	Graphical data	[6]
		Acetone	April Station 1 Distal portion	-	-	100 µL (200 mg of dry weight)	0-5 mm	Graphical data	[6]
		Acetone	April Station 1 Proximal portion	-	-	100 µL (200 mg of dry weight)	0-5 mm	Graphical data	[6]
		Ethanol	Septembre Station 1 Distal portion	-	-	100 µL (200 mg of dry weight)	15 mm	Graphical data	[6]
		Ethanol	Septembre Station 1 Proximal portion	-	-	100 µL (200 mg of dry weight)	10 mm	Graphical data	[6]
		Ethanol	April Station 1 Distal portion	-	-	100 µL (200 mg of dry weight)	0-5 mm	Graphical data	[6]
	Codium decorticatum	Ethyl acetate	-	-	-	100 µL	0.38	Data of inhibition not specified	[4]
	Codium fragile	Spring	-	-	-	Disc thallus	4,5 mm	-	[2]
		Summer	-	-	-	Disc thallus	4 mm	-	[2]
		Autumn	-	-	-	Disc thallus	2 mm	-	[2]
		Winter	-	-	-	Disc thallus	2 mm	-	[2]
	Codium intertextum	*n*-hexane	Winter	-	-	5 µL	10 mm	-	[9]
		Ethyl acetate	Autumn	-	-	5 µL	8 mm	-	[9]
		Methanol	Autumn	-	-	5 µL	16 mm	-	[9]
		Methanol	Winter	-	-	5 µL	28 mm	-	[9]

Bacillus subtilis	*Codium isthmocladum*	Chloroform	-	-	-	20 µL	7.0±0.0 mm	-	[5]
	Codium tomentosum	Dry powder	Spring	-	-	Disc thallus	4 mm	-	[2]
		Dry powder	Summer	-	-	Disc thallus	3 mm	-	[2]
		Dry powder	Autumn	-	-	Disc thallus	2,5 mm	-	[2]
		Dry powder	Winter	-	-	Disc thallus	2 mm	-	[2]
	Colpomenia sinuosa	Methanol	-	-	-	2 µg/mL	17 mm	Species analysed as human/animal pathogens	[10]
	Crassiphycus changii	Diethyl ether	-	2-hydroxymyristic acid	-	0.2 mg/disc	10.0 ±2 mm	-	[11]
		Diethyl ether	-	Cholesteryl myristate	-	0.2 mg/disc	8.6 ±1.15 mm	-	[11]
	Delesseria sanguinea	Dry powder	Spring	-	-	Disc thallus	2 mm	-	[2]
	Desmarestia aculeata Dictyopteris polypodioides	Dry powder	Spring	-	-	Disc thallus	8,5 mm	-	[2]
		Dry powder	Summer	-	-	Disc thallus	7 mm	-	[2]
		Dry powder	Autumn	-	-	Disc thallus	6 mm	-	[2]
		Dry powder	Winter	-	-	Disc thallus	2,5 mm	-	[2]
	Desmarestia ligulata	Dry powder	Summer	-	-	Disc thallus	20,5 mm	-	[2]
		Dry powder	Autumn	-	-	Disc thallus	20 mm	-	[2]
	Dictyopteris polypodioides	Dry powder	Spring	-	-	Disc thallus	4 mm	-	[2]
		Dry powder	Summer	-	-	Disc thallus	4 mm	-	[2]
		Dry powder	Autumn	-	-	Disc thallus	4 mm	-	[2]
		Dry powder	Winter	-	-	Disc thallus	1 mm	-	[2]
	Dictyota cervicornis	Ethanol	-	-	-	2 mg/disc	8 mm	-	[12]
		Ethanol	-	-	-	4 mg/disc	11 mm	-	[12]
		Ethanol	-	-	-	6 mg/disc	14 mm	-	[12]
	Dictyota dichotoma	Dry powder	Spring	-	-	Disc thallus	0,5 mm	-	[2]
		Dry powder	Summer	-	-	Disc thallus	4,5 mm	-	[2]
		Dry powder	Autumn	-	-	Disc thallus	2,5 mm	-	[2]
	Dictyota dichotoma var *intricata*	Ethanol	-	-	-	2 mg/disc	9 mm	-	[12]
		Ethanol	-	-	-	4 mg/disc	10 mm	-	[12]
		Ethanol	-	-	-	6 mg/disc	12 mm	-	[12]
	Digenea simplex	Ethanol	-	-	-	20 µL	7.3±0.6 mm	-	[5]

		Chloroform	-	-	-	20 µL	7.0±0.0 mm	-	[5]
	Dilsea carnosa	Dry powder	Spring	-	-	Disc thallus	9 mm	-	[2]
		Dry powder	Summer	-	-	Disc thallus	8 mm	-	[2]
		Dry powder	Autumn	-	-	Disc thallus	5 mm	-	[2]
Bacillus subtilis	*Dilsea carnosa*	Dry powder	Winter	-	-	Disc thallus	2,5 mm	-	[2]
	*Enteromorpha antenna**	Methanol	-	-	-	250 µg/mL (extract in DMSO)	10 mm	-	[13]
		Methanol	-	-	-	500 µg/mL (extract in DMSO)	11 mm	-	[13]
		Methanol	-	-	-	750 µg/mL (extract in DMSO)	12 mm	-	[13]
		Methanol	-	-	-	1000 µg/mL (extract in DMSO)	14 mm	-	[13]
	Ericaria selaginoides	Dry powder	Spring	-	-	Disc thallus	3 mm	-	[2]
		Dry powder	Summer	-	-	Disc thallus	3 mm	-	[2]
		Dry powder	Autumn	-	-	Disc thallus	2,5 mm	-	[2]
		Dry powder	Winter	-	-	Disc thallus	0,5 mm	-	[2]
	Eudesme virescens	Dry powder	Summer	-	-	Disc thallus	2 mm	-	[2]
		Dry powder	Autumn	-	-	Disc thallus	2 mm	-	[2]
	Gloiosiphonia capillaris	Dry powder	Summer	-	-	Disc thallus	27 mm	-	[2]
	Gongolaria baccata	Dry powder	Spring	-	-	Disc thallus	3 mm	-	[2]
		Dry powder	Summer	-	-	Disc thallus	2 mm	-	[2]
		Dry powder	Autumn	-	-	Disc thallus	2 mm	-	[2]
		Dry powder	Winter	-	-	Disc thallus	1 mm	-	[2]
	Gracilaria caudata	Ethanol	-	-	-	20 µL	12.3±0.6 mm	-	[5]
		Chloroform	-	-	-	20 µL	10.0±1.0 mm	-	[5]
	Gracilaria cornea	Ethanol	-	-	-	20 µL	8.3±0.6 mm	-	[5]
		Chloroform	-	-	-	20 µL	8.7±0.6 mm	-	[5]
	Gracilaria corticata	Methanol	-	-	-	750 µg/mL (extract in DMSO)	12 mm		[13]

		Methanol	-	-	-	1000 µg/mL (extract in DMSO)	15 mm		[13]
		70% methanol	-	Sulfurous acid, 2-ethylhexyl isohexyl ester, hexatriacontane, octacosane, mono (2-ethylhexyl) phthalate, 1-iodo-2-methylundecane and pentatriacontane	-	100 µg/mL	8 ± 0.01 mm	Antibacterial activity associated with fatty acids and suLfurous acid, 2-ethylhexyl isohexyl ester, eµgenol, benzene and phthalic acid	[14]
		70% methanol	-	Sulfurous acid, 2-ethylhexyl isohexyl ester, hexatriacontane, octacosane, mono (2-ethylhexyl) phthalate, 1-iodo-2-methylundecane and pentatriacontane	-	200 µg/mL	9 ± 0.03 mm	Antibacterial activity associated with fatty acids and suLfurous acid, 2-ethylhexyl isohexyl ester, eµgenol, benzene and phthalic acid	[14]
Bacillus subtilis	*Gracilaria corticata*	70% methanol	-	Sulfurous acid, 2-ethylhexyl isohexyl ester, hexatriacontane, octacosane, mono (2-ethylhexyl) phthalate, 1-iodo-2-methylundecane and pentatriacontane	-	300 µg/mL	11 ± 0.00 mm	Antibacterial activity associated with fatty acids and suLfurous acid, 2-ethylhexyl isohexyl ester, eµgenol, benzene and phthalic acid	[14]
		70% methanol	-	Sulfurous acid, 2-ethylhexyl isohexyl ester, hexatriacontane, octacosane, mono	-	400 µg/mL	14 ± 0.11 mm	Antibacterial activity associated with fatty acids and suLfurous acid,	[14]

				(2-ethylhexyl) phthalate, 1-iodo-2-methylundecane and pentatriacontane				2-ethylhexyl isohexyl ester, eμgenol, benzene and phthalic acid	
		70% methanol	-	Sulfurous acid, 2-ethylhexyl isohexyl ester, hexatriacontane, octacosane, mono (2-ethylhexyl) phthalate, 1-iodo-2-methylundecane and pentatriacontane	-	500 μg/mL	16 ± 0.02 mm	Antibacterial activity associated with fatty acids and suLfurous acid, 2-ethylhexyl isohexyl ester, eμgenol, benzene and phthalic acid	[14]
Bacillus subtilis	*Gracilaria corticata*	DMSO	-	Sulfurous acid, 2-ethylhexyl isohexyl ester, hexatriacontane, octacosane, mono (2-ethylhexyl) phthalate, 1-iodo-2-methylundecane and pentatriacontane	-	100 μg/mL	5 ± 0.12 mm	Antibacterial activity associated with fatty acids and suLfurous acid, 2-ethylhexyl isohexyl ester, eμgenol, benzene and phthalic acid	[14]
		DMSO	-	Sulfurous acid, 2-ethylhexyl isohexyl ester, hexatriacontane, octacosane, mono (2-ethylhexyl) phthalate, 1-iodo-2-methylundecane and pentatriacontane	-	200 μg/mL	6 ± 0.04 mm	Antibacterial activity associated with fatty acids and suLfurous acid, 2-ethylhexyl isohexyl ester, eμgenol, benzene and phthalic acid	[14]
		DMSO	-	Sulfurous acid, 2-ethylhexyl	-	300 μg/mL	7.5 ± 0.07 mm	Antibacterial activity	[14]

				isohexyl ester, hexatriacontane, octacosane, mono (2-ethylhexyl) phthalate, 1-iodo-2-methylundecane and pentatriacontane				associated with fatty acids and suLfurous acid, 2-ethylhexyl isohexyl ester, eµgenol, benzene and phthalic acid	
		DMSO	-	Sulfurous acid, 2-ethylhexyl isohexyl ester, hexatriacontane, octacosane, mono (2-ethylhexyl) phthalate, 1-iodo-2-methylundecane and pentatriacontane	-	400 µg/mL	10 ± 0.01 mm	Antibacterial activity associated with fatty acids and suLfurous acid, 2-ethylhexyl isohexyl ester, eµgenol, benzene and phthalic acid	[14]
Bacillus subtilis	*Gracilaria corticata*	DMSO	-	Sulfurous acid, 2-ethylhexyl isohexyl ester, hexatriacontane, octacosane, mono (2-ethylhexyl) phthalate, 1-iodo-2-methylundecane and pentatriacontane	-	500 µg/mL	12 ± 0.05 mm	Antibacterial activity associated with fatty acids and suLfurous acid, 2-ethylhexyl isohexyl ester, eµgenol, benzene and phthalic acid	[14]
		Acetone	Septembre Station 2 Distal portion	-	-	100 µL (200 mg of dry weight)	0-5 mm	Graphical data	[6]
		Acetone	Septembre Station 2 Proximal portion	-	-	100 µL (200 mg of dry weight)	5-10 mm	Graphical data	[6]
		Acetone	December Station 1 Distal portion	-	-	100 µL (200 mg of dry weight)	0-5 mm	Graphical data	[6]

		Acetone	December Station 1<br>Proximal portion	-	-	100 µL (200 mg of dry weight)	5-10 mm	Graphical data	[6]
		Acetone	December Station 2<br>Distal portion	-	-	100 µL (200 mg of dry weight)	0-5 mm	Graphical data	[6]
		Acetone	April<br>Station 1<br>Distal portion	-	-	100 µL (200 mg of dry weight)	5-10 mm	Graphical data	[6]
		Acetone	April<br>Station 1<br>Proximal portion	-	-	100 µL (200 mg of dry weight)	5-10 mm	Graphical data	[6]
		Acetone	April<br>Station 2<br>Distal portion	-	-	100 µL (200 mg of dry weight)	5-10 mm	Graphical data	[6]
		Acetone	April<br>Station 2<br>Proximal portion	-	-	100 µL (200 mg of dry weight)	5-10 mm	Graphical data	[6]
Bacillus subtilis	*Gracilaria corticata*	Ethanol	Septembre<br>Station 1<br>Distal portion	-	-	100 µL (200 mg of dry weight)	5-10 mm	Graphical data	[6]
		Ethanol	Septembre<br>Station 2<br>Distal portion	-	-	100 µL (200 mg of dry weight)	10-15 mm	Graphical data	[6]
		Ethanol	Septembre<br>Station 2<br>Proximal portion	-	-	100 µL (200 mg of dry weight)	15 mm	Graphical data	[6]
		Ethanol	December Station 1<br>Distal portion	-	-	100 µL (200 mg of dry weight)	5-10 mm	Graphical data	[6]
		Ethanol	December Station 1<br>Proximal portion	-	-	100 µL (200 mg of dry weight)	5-10 mm	Graphical data	[6]
		Ethanol	December Station 2<br>Distal portion	-	-	100 µL (200 mg of dry weight)	0-5 mm	Graphical data	[6]
		Ethanol	April<br>Station 1<br>Distal portion	-	-	100 µL (200 mg of dry weight)	5-10 mm	Graphical data	[6]
		Ethanol	April<br>Station 1	-	-	100 µL (200 mg of dry weight)	10-15 mm	Graphical data	[6]

			Proximal portion						
		Ethanol	April<br>Station 2<br>Distal portion	-	-	100 µL (200 mg of dry weight)	5-10 mm	Graphical data	[6]
		Ethanol	April<br>Station 2<br>Proximal portion	-	-	100 µL (200 mg of dry weight)	10-15 mm	Graphical data	[6]
	Gracilaria gracilis	Ethanol	-	High content in total soluble carbohydrate and total phenolic content (most abundant flavonoids: rutin and hesperidin)	-	50 µg (20 µL)	10 ± 0.00 mm	-	[15]
		Ethanol	-	High content in total soluble carbohydrate and total phenolic content (most abundant flavonoids: rutin and hesperidin)	-	100 µg (20 µL)	14.6 ± 0.5	-	[15]
Bacillus subtilis	*Gracilaria gracilis*	Ethanol	-	High content in total soluble carbohydrate and total phenolic content (most abundant flavonoids: rutin and hesperidin)	-	200 µg (20 µL)	19 ± 1 mm	Best activity registered in this study	[15]
		Methanol	-	High content in total soluble carbohydrate and total phenolic content (most abundant flavonoids: rutin and hesperidin)	-	50 µg (20 µL)	7.6 ± 2 mm	-	[15]
		Methanol	-	High content in total soluble	-	100 µg (20 µL)	9.6 ± 0.5 mm	-	[15]

				carbohydrate and total phenolic content (most abundant flavonoids: rutin and hesperidin)					
		Methanol	-	High content in total soluble carbohydrate and total phenolic content (most abundant flavonoids: rutin and hesperidin)	-	200 µg (20 µL)	12.6 ± 1.1 mm	-	[15]
		Acetone	-	High content in total phenol content (most abundant flavonoids: rutin and hesperidin)	-	50 µg (20 µL)	10 ± 0.00 mm	-	[15]
Bacillus subtilis	*Gracilaria gracilis*	Acetone	-	High content in total phenol content (most abundant flavonoids: rutin and hesperidin)	-	100 µg (20 µL)	11.6 ± 2.8 mm	-	[15]
		Acetone	-	High content in total phenol content (most abundant flavonoids: rutin and hesperidin)	-	200 µg (20 µL)	13.6 ± 3.5 mm	-	[15]
		Chloroform	-	Higher content in monounsaturated fatty acids and polyunsaturated fatty acids	-	50 µg (20 µL)	10.3 ± 0.5 mm	-	[15]
		Chloroform	-	Higher content in monounsaturated fatty acids and	-	100 µg (20 µL)	15.6 ± 3 mm	-	[15]

				polyunsaturated fatty acids					
		Chloroform	-	Higher content in monounsaturated fatty acids and polyunsaturated fatty acids	-	200 µg (20 µL)	17.6 ± 2 mm	-	[15]
		Diethyl ether	-	Higher content in saturated fatty acids	-	50 µg (20 µL)	10 ± 1 mm	-	[15]
		Diethyl ether	-	Higher content in saturated fatty acids	-	100 µg (20 µL)	10.6 ± 2 mm	-	[15]
		Diethyl ether	-	Higher content in saturated fatty acids	-	200 µg (20 µL)	15.6 ± 3 mm	-	[15]
	Gracilariopsis longissima	Dry powder	Winter	-	-	Disc thallus	2 mm	-	[2]
	Halidrys siliquosa	Dry powder	Spring	-	-	Disc thallus	4,5 mm	-	[2]
		Dry powder	Summer	-	-	Disc thallus	3,5 mm	-	[2]
Bacillus subtilis	*Halidrys siliquosa*	Dry powder	Autumn	-	-	Disc thallus	3,5 mm	-	[2]
		Dry powder	Winter	-	-	Disc thallus	1,5 mm	-	[2]
	Halimeda incrassata	Ethanol	-	-	-	20 µL	7.3±0.6 mm	-	[5]
		Chloroform	-	-	-	20 µL	8.7±0.6 mm	-	[5]
	Halimeda tuna	Ethanol	-	-	-	2 mg/disc	7 mm	-	[12]
		Ethanol	-	-	-	4 mg/disc	10 mm	-	[12]
		Ethanol	-	-	-	6 mg/disc	14 mm	-	[12]
		Methanol	-	-	-	0.1 mL	2-3 mm	-	[16]
	Halopithys incurva	Dry powder	Spring	-	-	Disc thallus	3 mm	-	[2]
		Dry powder	Summer	-	-	Disc thallus	1,5 mm	-	[2]
		Dry powder	Autumn	-	-	Disc thallus	1,5 mm	-	[2]
		Dry powder	Winter	-	-	Disc thallus	1 mm	-	[2]
	Halopterois scoparia	Ethyl acetate	Autumn	-	-	5 µL	12 mm	-	[9]
		Methanol	Autumn	-	-	5 µL	15 mm	-	[9]
		Methanol	Winter	-	-	5 µL	22 mm	-	[9]

	Halymenia floresia	Ethyl acetate partition of Methanols	-	-	-	100 µL	0.31	Data of inhibition not specified	[4]
	Hormophysa cuneiformis	Methanol	-	-	-	0.1 mL	2-3 mm	-	[16]
	Hydropuntia edulis	70% methanol	-	Eugenol, nonane, undecane, hept-2-ene, 2,4,4,6-tetramethyl, sulfurous acid, phthalic acid and 1,2-propanediol	-	100 µg/mL	3 ± 0.03 mm	Antibacterial activity associated with fatty acids and suLfurous acid, 2-ethylhexyl isohexyl ester, eµgenol, benzene and phthalic acid	[14]
		70% methanol	-	Eugenol, nonane, undecane, hept-2-ene, 2,4,4,6-tetramethyl, sulfurous acid, phthalic acid and 1,2-propanediol	-	200 µg/mL	3 ± 0.00 mm	Antibacterial activity associated with fatty acids and suLfurous acid, 2-ethylhexyl isohexyl ester, eµgenol, benzene and phthalic acid	[14]
		70% methanol	-	Eugenol, nonane, undecane, hept-2-ene, 2,4,4,6-tetramethyl, sulfurous acid, phthalic acid and 1,2-propanediol	-	300 µg/mL	4 ± 0.10 mm	Antibacterial activity associated with fatty acids and suLfurous acid, 2-ethylhexyl isohexyl ester, eµgenol, benzene and phthalic acid	[14]
Bacillus subtilis	*Hydropuntia edulis*	70% methanol	-	Eugenol, nonane, undecane, hept-2-ene, 2,4,4,6-tetramethyl, sulfurous acid,	-	400 µg/mL	5 ± 0.30 mm	Antibacterial activity associated with fatty acids and suLfurous acid,	[14]

				phthalic acid and 1,2-propanediol				2-ethylhexyl isohexyl ester, eµgenol, benzene and phthalic acid	
		70% methanol	-	Eugenol, nonane, undecane, hept-2-ene, 2,4,4,6-tetramethyl, sulfurous acid, phthalic acid and 1,2-propanediol	-	500 µg/mL	6 ± 0.01 mm	Antibacterial activity associated with fatty acids and suLfurous acid, 2-ethylhexyl isohexyl ester, eµgenol, benzene and phthalic acid	[14]
		DMSO	-	Eugenol, nonane, undecane, hept-2-ene, 2,4,4,6-tetramethyl, sulfurous acid, phthalic acid and 1,2-propanediol	-	100 µg/mL	5 ± 0.07 mm	Antibacterial activity associated with fatty acids and suLfurous acid, 2-ethylhexyl isohexyl ester, eµgenol, benzene and phthalic acid	[14]
		DMSO	-	Eugenol, nonane, undecane, hept-2-ene, 2,4,4,6-tetramethyl, sulfurous acid, phthalic acid and 1,2-propanediol	-	200 µg/mL	7 ± 0.06 mm	Antibacterial activity associated with fatty acids and suLfurous acid, 2-ethylhexyl isohexyl ester, eµgenol, benzene and phthalic acid	[14]
Bacillus subtilis	*Hydropuntia edulis*	DMSO	-	Eugenol, nonane, undecane, hept-2-ene, 2,4,4,6-tetramethyl, sulfurous acid,	-	300 µg/mL	7.5 ± 0.11 mm	Antibacterial activity associated with fatty acids and suLfurous acid, 2-ethylhexyl	[14]

				phthalic acid and 1,2-propanediol				isohexyl ester, eµgenol, benzene and phthalic acid	
		DMSO	-	Eugenol, nonane, undecane, hept-2-ene, 2,4,4,6-tetramethyl, sulfurous acid, phthalic acid and 1,2-propanediol	-	400 µg/mL	9 ± 0.15 mm	Antibacterial activity associated with fatty acids and suLfurous acid, 2-ethylhexyl isohexyl ester, eµgenol, benzene and phthalic acid	[14]
		DMSO	-	Eugenol, nonane, undecane, hept-2-ene, 2,4,4,6-tetramethyl, sulfurous acid, phthalic acid and 1,2-propanediol	-	500 µg/mL	15 ± 0.10 mm	Antibacterial activity associated with fatty acids and suLfurous acid, 2-ethylhexyl isohexyl ester, eµgenol, benzene and phthalic acid	[14]
	Iyengaria stellata	Methanol	-	-	-	2 µg/mL	14 mm	Species analysed as human/animal pathogens	[10]
Bacillus subtilis	*Kappaphycus alvarezii*	Aqueous (gel-like liquid)	-	Tannins, flavonoids, alkaloids, steroids and saponins.	-	40 µg/mL	4 mm	-	[17]
		Aqueous (gel-like liquid)	-	Tannins, flavonoids, alkaloids, steroids and saponins.	-	60 µg/mL	8 mm	-	[17]
		Aqueous (gel-like liquid)	-	Tannins, flavonoids, alkaloids, steroids and saponins.	-	80 µg/mL	8 mm	-	[17]

		Aqueous (gel-like liquid)	-	Tannins, flavonoids, alkaloids, steroids and saponins.	-	100 µg/mL	8 mm	-	[17]
	Laminaria digitata	Dry powder	Spring	-	-	Disc thallus	6,5 mm	-	[2]
		Dry powder	Summer	-	-	Disc thallus	4 mm	-	[2]
		Dry powder	Autumn	-	-	Disc thallus	2,5 mm	-	[2]
		Dry powder	Winter	-	-	Disc thallus	11,5 mm	-	[2]
	Laurencia obtusa	Ethanol	-	-	-	20 µL	7.3±0.6 mm	-	[5]
		Chloroform	-	-	-	20 µL	9.3±0.6 mm	-	[5]
		Dry powder	Spring	-	-	Disc thallus	4,5 mm	-	[2]
		Dry powder	Summer	-	-	Disc thallus	4,5 mm	-	[2]
		Dry powder	Autumn	-	-	Disc thallus	5 mm	-	[2]
		Dry powder	Winter	-	-	Disc thallus	4 mm	-	[2]
		Ethyl acetate	-	-	-	100 µL	0.36	Data of inhibition not specified	[4]
	Liaqura sp.*	Hexane	-	-	-	0.1 mL	2-3 mm	-	[16]
	Lychaete pellucida	Dry powder	Summer	-	-	Disc thallus	0,5 mm	-	[2]
		Dry powder	Autumn	-	-	Disc thallus	1 mm	-	[2]
	Melanothamnus afaqhusainii	Ethanol	-	-	-	2 mg/disc	9 mm	-	[12]
		Ethanol	-	-	-	4 mg/disc	9 mm	-	[12]
		Ethanol	-	-	-	6 mg/disc	10 mm	-	[12]
	Membranoptera alata	Dry powder	Spring	-	-	Disc thallus	2 mm	-	[2]
	Membranoptera alata	Dry powder	Summer	-	-	Disc thallus	1 mm	-	[2]
Bacillus subtilis	*Mesogloia vermiculata*	Dry powder	Summer	-	-	Disc thallus	2,5 mm	-	[2]
	Odonthalia dentata	Dry powder	Spring	-	-	Disc thallus	10 mm	-	[2]
		Dry powder	Summer	-	-	Disc thallus	11 mm	-	[2]
		Dry powder	Autumn	-	-	Disc thallus	10,5 mm	-	[2]
		Dry powder	Winter	-	-	Disc thallus	11 mm	-	[2]
	Osmundea hybrida	Dry powder	Spring	-	-	Disc thallus	5,5 mm	-	[2]
		Dry powder	Summer	-	-	Disc thallus	5 mm	-	[2]
		Dry powder	Autumn	-	-	Disc thallus	5 mm	-	[2]

		Dry powder	Winter	-	-	Disc thallus	5 mm	-	[2]
	Osmundea pinnatifida	Dry powder	Spring	-	-	Disc thallus	7,5 mm	-	[2]
		Dry powder	Summer	-	-	Disc thallus	7 mm	-	[2]
		Dry powder	Autumn	-	-	Disc thallus	7 mm	-	[2]
		Dry powder	Winter	-	-	Disc thallus	8,5 mm	-	[2]
	Padina gymnospora	Hexane	-	-	-	2.5 mg/disc	8.33±0.58 mm	-	[8]
		Hexane	-	-	-	5 mg/disc	8.67±0.58 mm	-	[8]
		Ethyl acetate	-	-	-	2.5 mg/disc	7.33±0.58 mm	-	[8]
		Ethyl acetate	-	-	-	5 mg/disc	8.33±0.58 mm	-	[8]
		Acetone	-	-	-	1.25 mg/disc	8.33±0.58 mm	-	[8]
		Acetone	-	-	-	2.5 mg/disc	10.00±0.00 mm	-	[8]
		Acetone	-	-	-	5 mg/disc	11.67±0.58 mm	-	[8]
	Padina sp.	Hexane	-	-	-	0.1 mL	8-12 mm	-	[16]
	Penicillus capitatus	Ethanol	-	-	-	20 µL	8.3±0.6 mm	-	[5]
		Chloroform	-	-	-	20 µL	8.7±0.6 mm	-	[5]
	Petrospongium berkeleyi	Dry powder	Summer	-	-	Disc thallus	1 mm	-	[2]
		Dry powder	Autumn	-	-	Disc thallus	1,5 mm	-	[2]
	Phyllophora crispa	Dry powder	Spring	-	-	Disc thallus	2 mm	-	[2]
	Phyllophora pseudoceranoïdes	Dry powder	Spring	-	-	Disc thallus	2,5 mm	-	[2]
	Polysiphonia stricta	Dry powder	Spring	-	-	Disc thallus	8 mm	-	[2]
		Dry powder	Summer	-	-	Disc thallus	8,5 mm	-	[2]
		Dry powder	Autumn	-	-	Disc thallus	8 mm	-	[2]
		Dry powder	Winter	-	-	Disc thallus	8,5 mm	-	[2]
	Pterothamnion plumula	Dry powder	Spring	-	-	Disc thallus	2 mm	-	[2]
		Dry powder	Summer	-	-	Disc thallus	1,5 mm	-	[2]
Bacillus subtilis	*Pterothamnion plumula*	Dry powder	Autumn	-	-	Disc thallus	1 mm	-	[2]
	Ptilophora subcostata	Crude extract	-	-	-	60 µl	>10 mm	-	[18]
		Crude extract	-	-	-	60 µl	>10 mm	-	[18]
		Crude extract	-	-	-	60 µl	>10 mm	-	[18]
		Crude extract	-	-	-	60 µl	>10 mm	-	[18]
		Crude extract	-	-	-	60 µl	>10 mm	-	[18]
	Rhodomela confervoides	Dry powder	Summer	-	-	Disc thallus	2 mm	-	[2]
		Dry powder	Autumn	-	-	Disc thallus	1,5 mm	-	[2]

		Dry powder	Winter	-	-	Disc thallus	1,5 mm	-	[2]
	Saccharina latissima	Dry powder	Spring	-	-	Disc thallus	5,5 mm	-	[2]
		Dry powder	Winter	-	-	Disc thallus	8,5 mm	-	[2]
	Sargassum desfontainesii	Ethyl acetate	Autumn	-	-	5 µL	10 mm	-	[9]
		Ethyl acetate	Winter	-	-	5 µL	8 mm	-	[9]
		Methanol	Autumn	-	-	5 µL	13 mm	-	[9]
		Methanol	Winter	-	-	5 µL	11 mm	-	[9]
	Sargassum filipendula	Chloroform	-	-	-	20 µL	7.7±0.6 mm	-	[5]
		Ethyl acetate	-	-	-	100 µL	0.38	Data of inhibition not specified (units)	[4]
	Sargassum hystrix	Ethyl acetate	-	-	-	100 µL	0.36	Data of inhibition not specified (units)	[4]
	Sargassum lanceolatum	Ethanol	-	-	-	2 mg/disc	8 mm	-	[12]
		Ethanol	-	-	-	4 mg/disc	10 mm	-	[12]
		Ethanol	-	-	-	6 mg/disc	12 mm	-	[12]
	Sargassum polycystum	Acetone		Tannins, steroids.	Tannins, flavonoids, terpenoids, cardiac glycosydes, phlobatannins, steroids.	-	10	Graphical data (no units)	[19]
		Ethanol		Steroids.	Flavonoids, terpenoids, cardiac glycosydes, steroids.	-	8	Graphical data (no units)	[19]
	Sargassum polycystum	Water	-	-	Phenols, amino acids, proteins.	-	5	Graphical data (no units)	[19]
	Sargassum muticum	Methanol	-	Phenolic compounds (mainly flavonoids).	-	300 mg/mL	19.66 mm	-	[20]
		Water	-	-	-	300 mg/mL	8.67 mm		[20]
	Sargassum sp.	Methanol	-	-	-	0.1 mL	2-3 mm	-	[16]
Bacillus subtilis	*Sargassum* sp.	Hexane	-	-	-	0.1 mL	2-3 mm	-	[16]

	Sargassum tenerrimum	Aqueous (gel-like liquid)	-	Tannins, flavonoids, alkaloids, steroids, saponins, phlorotannins and terpenoids.	-	40 µg/mL	4 mm	-	[17]
		Aqueous (gel-like liquid)	-	Tannins, flavonoids, alkaloids, steroids, saponins, phlorotannins and terpenoids.	-	60 µg/mL	5 mm	-	[17]
		Aqueous (gel-like liquid)	-	Tannins, flavonoids, alkaloids, steroids, saponins, phlorotannins and terpenoids.	-	80 µg/mL	6 mm	-	[17]
		Aqueous (gel-like liquid)	-	Tannins, flavonoids, alkaloids, steroids, saponins, phlorotannins and terpenoids.	-	100 µg/mL	9 mm	-	[17]
		Methanol	-	Amino acids, alkaloids, carbohydrates, saponins, sterols, terpenoids, proteins, and phenolic compounds.	-	-	12 mm	Graphical data	[21]
		Methanol	-	Cholest-5-en-3-ol, 24-propylidene-, (3á)-; 1,2-Benzenedicarboxylic acid, diisooctyl	-	Not specified.	19.3±1.2 mm	1,2-benzenedicarboxylic acid, diisooctyl ester, 1-docosene, 1,2-	[22]

				ester; Hentriacontane; 1-Docosene; 1-Nonadecene; 1-Hexadecanol; 1,2-Benzenediol; Benzoic acid.				benzenediol and benzoic acid are indicated as the responsible compounds for the antibacterial activity.	
		Petroleum ether	-	Benzoic acid, 3,5-dicyclohexyl-4-hydroxy-, methyl ester; Isomethadone; Cholesterol; Squalene; 9-Hexadecenoic acid, eicosyl ester, (Z)-; 17-Pentatriacontene; Dasycarpidan-1-methanol, acetate (ester); Hexadecanoic acid, methyl ester.	-	Not specified.	16.6±2.5 mm	Hexadecenoic acid, methyl ester, 17-pentatriaconten e, dasycarpian-1-methanol, and acetate are indicated as the responsible compounds for the antibacterial activity.	[22]
	Sargassum wightii	Water	-	-	-	5 µg	6 mm	-	[23]
Bacillus subtilis	*Sargassum wightii*	Water	-	-	-	10 µg	7 mm	-	[23]
		Water	-	-	-	15 µg	9 mm	-	[23]
		Acetone	-	Steroids, terpenoids, glycosides, alkaloids, flavonoids, tannins and saponins	-	-	15±1.4 mm	-	[24][24]
		Diethyl ether	-	Steroids, terpenoids, glycosides, flavonoids and saponins	-	-	14±1.2 mm	-	[24]

		Methanol	-	Steroids, terpenoids, alkaloids, flavonoids, tannins and saponins	-	-	12.3±0.2 mm	-	[24]
		Hexane	-	-	-	1.25 mg/disc	8.33±0.58 mm	-	[8]
		Hexane	-	-	-	2.5 mg/disc	10.33±0.58 mm	-	[8]
		Hexane	-	-	-	5 mg/disc	10.67±0.58 mm	-	[8]
		Ethyl acetate	-	-	-	1.25 mg/disc	8.67±0.58 mm	-	[8]
		Ethyl acetate	-	-	-	2.5 mg/disc	10.33±0.58 mm	-	[8]
		Ethyl acetate	-	-	-	5 mg/disc	11.33±0.58 mm	-	[8]
		Acetone	-	-	-	1.25 mg/disc	9.33±0.58 mm	-	[8]
		Acetone	-	-	-	2.5 mg/disc	10.00±0.00 mm	-	[8]
		Acetone	-	-	-	5 mg/disc	11.33±0.58 mm	-	[8]
	Sphondylothamnion multifidum	Dry powder	Summer	-	-	Disc thallus	16 mm	-	[2]
		Dry powder	Autumn	-	-	Disc thallus	16,5 mm	-	[2]
	Stypopodium zonale	*n*-hexane	Autumn	-	-	5 µL	11 mm	-	[9]
		Ethyl acetate	Autumn	-	-	5 µL	15 mm	-	[9]
		Ethyl acetate	Winter	-	-	5 µL	12 mm	-	[9]
	Stypopodium zonale	Methanol	Autumn	-	-	5 µL	13 mm	-	[9]
	Symphyocladiella parasitica	-	Summer	-	-	Disc thallus	2 mm	-	[2]
Bacillus subtilis	*Turbinaria ornata*	Methanol	-	-	-	0.1 mL	4-7 mm	-	[16]
	Udotea occidentalis	Ethanol	-	-	-	20 µL	12.0±1.0 mm	-	[5]
		Chloroform	-	-	-	20 µL	14.0±0.0 mm	-	[5]
	Ulva lactuca	Dry powder	Winter	-	-	Disc thallus	4 mm	-	[2]
		Acetone	Septembre station 1 Distal portion	-	-	100 µL (200 mg of dry weight)	5-10 mm	Graphical data	[6]
		Acetone	Septembre station 1 Proximal portion	-	-	100 µL (200 mg of dry weight)	5-10 mm	Graphical data	[6]
		Acetone	April station 1 Distal portion	-	-	100 µL (200 mg of dry weight)	5-10 mm	Graphical data	[6]

		Acetone	April<br>station 1<br>Proximal portion	-	-	100 µL (200 mg of dry weight)	0-5 mm	Graphical data	[6]
		Acetone	April<br>station 2<br>Distal portion	-	-	100 µL (200 mg of dry weight)	15 mm	Graphical data	[6]
		Acetone	April<br>station 2<br>Proximal portion	-	-	100 µL (200 mg of dry weight)	5 mm	Graphical data	[6]
		Ethanol	Septembre<br>station 1<br>Distal portion	-	-	100 µL (200 mg of dry weight)	15-20 mm	Graphical data	[6]
		Ethanol	Septembre<br>station 1<br>Proximal portion	-	-	100 µL (200 mg of dry weight)	5-10 mm	Graphical data	[6]
		Ethanol	April<br>station 1<br>Distal portion	-	-	100 µL (200 mg of dry weight)	0-5 mm	Graphical data	[6]
		Ethanol	April<br>station 1<br>Proximal portion	-	-	100 µL (200 mg of dry weight)	0-5 mm	Graphical data	[6]
		Ethanol	April<br>station 2<br>Distal portion	-	-	100 µL (200 mg of dry weight)	5 mm	Graphical data	[6]
	Ulva lactuca	Ethanol	April<br>station 2<br>Proximal portion	-	-	100 µL (200 mg of dry weight)	5 mm	Graphical data	[6]
Bacillus subtilis	*Ulva linza*	Methanol	-	-	-	750 µg/mL (extract in DMSO)	11 mm	The antibacterial activity in algae have been reported in bromophenols, carbonyls, halogenated aliphatic compounds, terpenes, isoprenylated and brominated hydroquinones,	[13]

								as well as phlorotannins	
		Methanol	-	-	-	1000 µg/mL (extract in DMSO)	14 mm	The antibacterial activity in algae have been reported in bromophenols, carbonyls, halogenated aliphatic compounds, terpenes, isoprenylated and brominated hydroquinones, as well as phlorotannins	[13]
	Ulva rigida	*n*-hexane	Winter	-	-	5 µL	10 mm	-	[9]
		Methanol	Autumn	-	-	5 µL	13 mm	-	[9]
		Methanol	Winter	-	-	5 µL	18 mm	-	[9]
	Vertebrata byssoides	Dry powder	Spring	-	-	Disc thallus	9,5 mm	-	[2]
		Dry powder	Summer	-	-	Disc thallus	6 mm	-	[2]
	Vertebrata byssoides	Dry powder	Autumn	-	-	Disc thallus	6 mm	-	[2]
	Vertebrata fucoides	Dry powder	Spring	-	-	Disc thallus	8 mm	-	[2]
Bacillus subtilis	*Vertebrata fucoides*	Dry powder	Summer	-	-	Disc thallus	9 mm	-	[2]
		Dry powder	Autumn	-	-	Disc thallus	8,5 mm	-	[2]
		Dry powder	Winter	-	-	Disc thallus	9 mm	-	[2]
	Vertebrata lanosa	Dry powder	Spring	-	-	Disc thallus	15 mm	-	[2]
		Dry powder	Summer	-	-	Disc thallus	15,5 mm	-	[2]
		Dry powder	Autumn	-	-	Disc thallus	15,5 mm	-	[2]
		Dry powder	Winter	-	-	Disc thallus	15,5 mm	-	[2]
	Vertebrata nigra	Dry powder	Spring	-	-	Disc thallus	9 mm	-	[2]
		Dry powder	Summer	-	-	Disc thallus	8,5 mm	-	[2]
		Dry powder	Autumn	-	-	Disc thallus	9 mm	-	[2]
		Dry powder	Winter	-	-	Disc thallus	9 mm	-	[2]
		Dry powder	Spring	-	-	Disc thallus	2,5 mm	-	[2]

	Vertebrata thuyoides	Dry powder	Summer	-	-	Disc thallus	3,5 mm	-	[2]
		Dry powder	Autumn	-	-	Disc thallus	4 mm	-	[2]
		Dry powder	Winter	-	-	Disc thallus	3 mm	-	[2]
	Yuzurua poiteaui	Ethanol	-	-	-	20 µL	8.0±0.0 mm	-	[5]
		Chloroform	-	-	-	20 µL	14.0±2.0 mm	-	[5]
Corynebacterium diptheriae	*Codium shameelii*	Methanol	-	-	-	2 µg/mL	13	Species analysed as human/animal pathogens	[10]
	Colpomenia sinuosa	Methanol	-	-	-	2 µg/mL	14	Species analysed as human/animal pathogens	[10]
	Dictyota hauckiana	Methanol	-	-	-	2 µg/mL	11	Species analysed as human/animal pathogens	[10]
	Iyengaria stellata	Methanol	-	-	-	2 µg/mL	15	Species analysed as human/animal pathogens	[10]
	Polycladia indica	Methanol	-	-	-	2 µg/mL	13	Species analysed as human/animal pathogens	[10]
Corynebacterium diptheriae	*Stoechospermum polypodioides*	Methanol	-	-	-	2 µg/mL	12	Species analysed as human/animal pathogens	[10]
Erwinia amylovora	*Chaetomorpha linum*	Ethyl acetate	-	-	-	1.25 mg/disc	10.33±0.58 mm	-	[8]
		Ethyl acetate	-	-	-	2.5 mg/disc	11.33±0.58 mm	-	[8]
		Ethyl acetate	-	-	-	5 mg/disc	12.00±1.00 mm	-	[8]
		Acetone	-	-	-	1.25 mg/disc	12.67±0.58 mm	-	[8]
		Acetone	-	-	-	2.5 mg/disc	14.67±0.58 mm	-	[8]
		Acetone	-	-	-	5 mg/disc	16.67±0.58 mm	-	[8]
	Padina gymnospora	Hexane	-	-	-	2.5 mg/disc	6.33±0.58 mm	-	[8]
		Hexane	-	-	-	5 mg/disc	7.33±0.58 mm	-	[8]
		Ethyl acetate	-	-	-	2.5 mg/disc	7.33±0.58 mm	-	[8]

		Ethyl acetate	-	-	-	5 mg/disc	8.33±0.58 mm	-	[8]
		Acetone	-	-	-	1.25 mg/disc	9.00±0.00 mm	-	[8]
		Acetone	-	-	-	2.5 mg/disc	10.67±0.58 mm	-	[8]
		Acetone	-	-	-	5 mg/disc	15.33±0.58 mm	-	[8]
	Sargassum wightii	Hexane	-	-	-	1.25 mg/disc	8.33±0.58 mm	-	[8]
		Hexane	-	-	-	2.5 mg/disc	11.00±0.00 mm	-	[8]
		Hexane	-	-	-	5 mg/disc	12.33±0.58 mm	-	[8]
		Ethyl acetate	-	-	-	2.5 mg/disc	8.67±0.58 mm	-	[8]
		Ethyl acetate	-	-	-	5 mg/disc	10.33±0.58 mm	-	[8]
		Acetone	-	-	-	1.25 mg/disc	11.33±0.58 mm	-	[8]
		Acetone	-	-	-	2.5 mg/disc	12.33±0.58 mm	-	[8]
		Acetone	-	-	-	5 mg/disc	13.33±0.58 mm	-	[8]
		Methanol	-	-	-	5 mg/disc	10.00±0.00 mm	-	[8]
Erwinia chrysanthemi	*Halopithys incurva*	Methanol	-	-	-	-	16±1.41 mm	-	[25]
		Dichloromethane	-	-	-	-	8±2.12 mm	-	[25]
		Dichloromethane :methanol (50:50)	-	-	-	-	12±1.87 mm	-	[25]
	Bifurcaria bifurcata	Methanol	-	-	-	-	12±1.63 mm	-	[25]
		Dichloromethane	-	-	-	-	18±2.12 mm	-	[25]
		Dichloromethane :methanol (50:50)	-	-	-	-	15±3.67 mm	-	[25]
Erwinia chrysanthemi	*Codium decorticatum*	Methanol	-	-	-	-	8±2.94 mm	-	[25]
		Dichloromethane	-	-	-	-	10±0.71 mm	-	[25]
		Dichloromethane :methanol (50:50)	-	-	-	-	8±4.47 mm	-	[25]
	Cystoseira humilis var. *myriophylloides*	Methanol	-	-	-	-	9±2.00 mm	-	[25]
		Dichloromethane	-	-	-	-	10±1.87 mm	-	[25]
		Dichloromethane :methanol (50:50)	-	-	-	-	7±2.12 mm	-	[25]
	Ellisolandia elongata	Methanol	-	-	-	-	18±1.41 mm	-	[25]
		Dichloromethane	-	-	-	-	12±1.73 mm	-	[25]

		Dichloromethane :methanol (50:50)	-	-	-	-	16±1.22 mm	-	[25]
	Ericaria selaginoides	Methanol	-	-	-	-	8±2.31 mm	-	[25]
		Dichloromethane	-	-	-	-	8±3.54 mm	-	[25]
		Dichloromethane :methanol (50:50)	-	-	-	-	8±2.55 mm	-	[25]
	Fucus spiralis	Methanol	-	-	-	-	10±0.82 mm	-	[25]
		Dichloromethane	-	-	-	-	8±3.61 mm	-	[25]
		Dichloromethane :methanol (50:50)	-	-	-	-	12±1.87 mm	-	[25]
	Gelidium corneum	Methanol	-	-	-	-	9±3.56 mm	-	[25]
		Dichloromethane	-	-	-	-	9±3.08 mm	-	[25]
		Dichloromethane :methanol (50:50)	-	-	-	-	12±1.87 mm	-	[25]
	Gelidium sp	Methanol	-	-	-	-	11±1.15 mm	-	[25]
		Dichloromethane	-	-	-	-	10±1.22 mm	-	[25]
		Dichloromethane :methanol (50:50)	-	-	-	-	10±1.22 mm	-	[25]
	Gracilaria cervicornis	Methanol	-	-	-	-	8±2.94 mm	-	[25]
		Dichloromethane	-	-	-	-	11±1.00 mm	-	[25]
Erwinia chrysanthemi	*Gracilaria cervicornis*	Dichloromethane :methanol (50:50)	-	-	-	-	17±1.58 mm	-	[25]
	Gymnogongrus crenulatus	Methanol	-	-	-	-	10±0.82 mm	-	[25]
		Dichloromethane	-	-	-	-	12±1.87 mm	-	[25]
		Dichloromethane :methanol (50:50)	-	-	-	-	8±4.47 mm	-	[25]
	Laminaria digitata	Methanol	-	-	-	-	8±2.94 mm	-	[25]
		Dichloromethane	-	-	-	-	13±1.58 mm	-	[25]
		Dichloromethane :methanol (50:50)	-	-	-	-	15±3.08 mm	-	[25]

	Osmundea pinnatifida	Methanol	-	-	-	-	8±2.94 mm	-	[25]
		Dichloromethane	-	-	-	-	8±4.74 mm	-	[25]
		Dichloromethane :methanol (50:50)	-	-	-	-	8±4.06 mm	-	[25]
	Plocamium cartilagineum	Methanol	-	-	-	-	14±1.15 mm	-	[25]
		Dichloromethane	-	-	-	-	11±2.24 mm	-	[25]
		Dichloromethane :methanol (50:50)	-	-	-	-	11±2.24 mm	-	[25]
	Sargassum vulgare	Methanol	-	-	-	-	8±2.83 mm	-	[25]
		Dichloromethane	-	-	-	-	10±1.22 mm	-	[25]
		Dichloromethane :methanol (50:50)	-	-	-	-	12±1.41 mm	-	[25]
	Ulva intestinalis	Methanol	-	-	-	-	9.3±0.58 mm	-	[25]
		Dichloromethane	-	-	-	-	9±1.73 mm	-	[25]
		Dichloromethane :methanol (50:50)	-	-	-	-	5.33±4.73 mm	-	[25]
	Ulva sp.	Methanol	-	-	-	-	10±0.82 mm	-	[25]
		Dichloromethane	-	-	-	-	10±1.58 mm	-	[25]
		Dichloromethane :methanol (50:50)	-	-	-	-	10±1.22 mm	-	[25]
Escherichia coli	*Kappaphycus alvarezii*	Aqueous (gel-like liquid)	-	Tannins, flavonoids, alkaloids, steroids and saponins.	-	40 µg/mL	-	-	[17]
		Aqueous (gel-like liquid)	-	Tannins, flavonoids, alkaloids, steroids and saponins.	-	60 µg/mL	4 mm	-	[17]
		Aqueous (gel-like liquid)	-	Tannins, flavonoids, alkaloids, steroids and saponins.	-	80 µg/mL	2 mm	-	[17]
		Aqueous (gel-like liquid)	-	Tannins, flavonoids,	-	100 µg/mL	6 mm	-	[17]

				alkaloids, steroids and saponins.					
	Sargassum muticum	Methanol	-	Phenolic compounds (mainly flavonoids).	-	300 mg/mL	24.33 mm	-	[20]
		Water	-	-	-	300 mg/mL	11.33 mm	-	[20]
	Sargassum tenerrimum	Aqueous (gel-like liquid)	-	Tannins, flavonoids, alkaloids, steroids, saponins, phlorotannins and terpenoids.	-	40 µg/mL	2 mm	-	[17]
		Aqueous (gel-like liquid)	-	Tannins, flavonoids, alkaloids, steroids, saponins, phlorotannins and terpenoids.	-	60 µg/mL	7 mm	-	[17]
		Aqueous (gel-like liquid)	-	Tannins, flavonoids, alkaloids, steroids, saponins, phlorotannins and terpenoids.	-	80 µg/mL	8 mm	-	[17]
		Aqueous (gel-like liquid)	-	Tannins, flavonoids, alkaloids, steroids, saponins, phlorotannins and terpenoids.	-	100 µg/mL	10 mm	-	[17]
	Sargassum tenerrimum	Methanol	-	Cholest-5-en-3-ol, 24-propylidene-, (3á)-; 1,2-Benzenedicarboxylic acid, diisooctyl	-	Not specified.	20.6±1.6 mm	1,2-benzenedicarboxylic acid, diisooctyl ester, 1-docosene, 1,2-	[22]

				ester; Hentriacontane; 1-Docosene; 1-Nonadecene; 1-Hexadecanol; 1,2-Benzenediol; Benzoic acid.				benzenediol and benzoic acid are indicated as the responsible compounds for the antibacterial activity.	
		Petroleum ether	-	Benzoic acid, 3,5-dicyclohexyl-4-hydroxy-, methyl ester; Isomethadone; Cholesterol; Squalene; 9-Hexadecenoic acid, eicosyl ester, (Z)-; 17-Pentatriacontene; Dasycarpidan-1-methanol, acetate (ester); Hexadecanoic acid, methyl ester.	-	Not specified.	12.5±1.2 mm	Hexadecenoic acid, methyl ester, 17-pentatriaconten e, dasycarpian-1-methanol, and acetate are indicated as the responsible compounds for the antibacterial activity.	[22]
	Sargassum cristaefolium	Hexane	-	Phenol, 1-nonadecene, myristic acid, 9-tricosene (Z)-, palmitic acid, 1-hexacosene and oleic acid.	-	2 mg/mL	3.71±0.07 mm	-	[26]
	Sargassum cristaefolium	Serial extraction with hexane, and then ethyl acetate	-	Phenol, 1-hexadecene, myristic acid, 9-tricosene (Z)-, neophytadiene, phytol, cyclotetracosane, palmitic acid, and oleic acid.	-	2 mg/mL	3.54±0.07 mm	-	[26]

	Sargassum cristaefolium	Serial extraction with hexane, ethyl acetate and methanol, respectively.	-	Phenol, methyl dihydrojasmonate , 3-azepan-1-yl-benzo[d]isothiazo le1,1-dioxide, hexyl cinnamic aldehyde, 9-tricosene(Z)-, palmitic acid, 1-hexacosene, hexatriacontane, and 1-docosene.	-	2 mg/mL	5.42±0.14 mm	-	[26]
Lactobacillus acidophilius	*Kappaphycus alvarezii*	Aqueous (gel-like liquid)	-	Tannins, flavonoids, alkaloids, steroids and saponins.	-	40 µg/mL	-	-	[17]
		Aqueous (gel-like liquid)	-	Tannins, flavonoids, alkaloids, steroids and saponins.	-	60 µg/mL	1 mm	-	[17]
		Aqueous (gel-like liquid)	-	Tannins, flavonoids, alkaloids, steroids and saponins.	-	80 µg/mL	2 mm	-	[17]
		Aqueous (gel-like liquid)	-	Tannins, flavonoids, alkaloids, steroids and saponins.	-	100 µg/mL	4 mm	-	[17]
	Sargassum tenerrimum	Aqueous (gel-like liquid)	-	Tannins, flavonoids, alkaloids, steroids, saponins, phlorotannins and terpenoids.	-	40 µg/mL	2 mm	-	[17]
		Aqueous (gel-like liquid)	-	Tannins, flavonoids, alkaloids, steroids,	-	60 µg/mL	3 mm	-	[17]

				saponins, phlorotannins and terpenoids.					
		Aqueous (gel-like liquid)	-	Tannins, flavonoids, alkaloids, steroids, saponins, phlorotannins and terpenoids.	-	80 µg/mL	5 mm	-	[17]
Lactobacillus acidophilius	*Sargassum tenerrimum*	Aqueous (gel-like liquid)	-	Tannins, flavonoids, alkaloids, steroids, saponins, phlorotannins and terpenoids.	-	100 µg/mL	7 mm	-	[17]
Proteus mirabilis	*Kappaphycus alvarezii*	Aqueous (gel-like liquid)	-	Tannins, flavonoids, alkaloids, steroids and saponins.	-	40 µg/mL	2 mm	-	[17]
		Aqueous (gel-like liquid)	-	Tannins, flavonoids, alkaloids, steroids and saponins.	-	60 µg/mL	6 mm	-	[17]
		Aqueous (gel-like liquid)	-	Tannins, flavonoids, alkaloids, steroids and saponins.	-	80 µg/mL	8 mm	-	[17]
		Aqueous (gel-like liquid)	-	Tannins, flavonoids, alkaloids, steroids and saponins.	-	100 µg/mL	7 mm	-	[17]
	Sargassum tenerrimum	Aqueous (gel-like liquid)	-	Tannins, flavonoids, alkaloids, steroids, saponins, phlorotannins and terpenoids.	-	40 µg/mL	7 mm	-	[17]

		Aqueous (gel-like liquid)	-	Tannins, flavonoids, alkaloids, steroids, saponins, phlorotannins and terpenoids.	-	60 µg/mL	8 mm	-	[17]
		Aqueous (gel-like liquid)	-	Tannins, flavonoids, alkaloids, steroids, saponins, phlorotannins and terpenoids.	-	80 µg/mL	12 mm	-	[17]
Proteus mirabilis	*Sargassum tenerrimum*	Aqueous (gel-like liquid)	-	Tannins, flavonoids, alkaloids, steroids, saponins, phlorotannins and terpenoids.	-	100 µg/mL	9 mm	-	[17]
Pseudomonas aeruginosa	*Sargassum lanceolatum*	Ethanol	-	-	-	4 mg/disc	8 mm	Produced weak zone and later bacterial growth	[12]
		Ethanol	-	-	-	6 mg/disc	8 mm	Produced weak zone and later bacterial growth	[12]
	Chaetomorpha antennina	Petroleum ether	-	-	-	50 µg/mL (dissolved in DMSO)	7.3 ± 1 mm	-	[7]
		Petroleum ether	-	-	-	100 µg/mL (dissolved in DMSO)	11.16 ± 1.4 mm	-	[7]
		Petroleum ether	-	-	-	200 µg/mL (dissolved in DMSO)	12.16 ± 1.3 mm	-	[7]
		Petroleum ether	-	-	-	300 µg/mL (dissolved in DMSO)	15.16 ± 1.3 mm	-	[7]

		Petroleum ether	-	-	-	400 µg/mL (dissolved in DMSO)	15.83 ± 0.9 mm	-	[7]
		Petroleum ether	-	-	-	500 µg/mL (dissolved in DMSO)	16.83 ± 0.9 mm	-	[7]
	Chaetomorpha linum	Hexane	-	-	-	2.5 mg/disc (extract)	7.3±0.58 mm	-	[8]
		Hexane	-	-	-	5 mg/disc	9.67±0.58 mm	-	[8]
	Chaetomorpha sp.	Methanol	-	-	-	50 µl, 75 µl, 100 µl	5 mm	-	[27]
	Codium intertextum	Methanol	(Winter)	-	-	5 µL	10 mm	-	[9]
Pseudomonas aeruginosa	*Codium shameelii*	Methanol	-	-	-	2 µg/mL	12	Species analysed as human/animal pathogens	[10]
	Colpomenia sinuosa	Methanol	-	-	-	2 µg/mL	13	Species analysed as human/animal pathogens	[10]
	Dictyopteris polypodioides	Methanol	-	-	Phlorotannins present in brown algae	-	15 mm	-	[28]
	Dictyota cervicornis	Ethanol	-	-	-	4 mg/disc	7 mm	Produced weak zone and later bacteria grown.	[12]
		Ethanol	-	-	-	6 mg/disc	8 mm	Produced weak zone and later bacteria grown.	[12]
	Dictyota dichotoma var *intricata*	Ethanol	-	-	-	2 mg/disc	9 mm	Produced weak zone and later bacteria grown.	[12]
		Ethanol	-	-	-	4 mg/disc	11 mm	Produced weak zone and later bacteria grown.	[12]
		Ethanol	-	-	-	6 mg/disc	12 mm	Produced weak zone and later bacteria grown.	[12]
	Gracilaria corticata	Methanol (Soxhlet/hot)	-	Phenolic compounds,	Saponins, alkaloids,	125 µg/ml	7 mm	The presence of tannins at low	[29]

				terpenoids, steroids, sugars	phenols, steroids and triterpenoids and free hydroxyl groups			concentration and terpenoids have antimicrobial activity.	
		Isopropanol (Soxhlet/hot)	-	Phenols, steroids, glycosides	Saponins, alkaloids, phenols, steroids and triterpenoids and free hydroxyl groups	125 µg/ml	13 mm	The presence of tannins at low concentration have antimicrobial activity	[29]
Pseudomonas aeruginosa	*Gracilaria corticata*	Benzene (Cold)	-	Alkaloids, phenols, saponins, steroids.	Saponins, alkaloids, phenols, steroids and triterpenoids and free hydroxyl groups	125 µg/ml	9 mm	The presence of tannins at low concentration and saponins have antimicrobial activity	[29]
	Halimeda tuna	Ethanol	-	-	-	2 mg/disc	7 mm	Produced weak zone and later bacteria grown.	[12]
		Ethanol	-	-	-	4 mg/disc	8 mm	Produced weak zone and later bacteria grown.	[12]
		Ethanol	-	-	-	6 mg/disc	10 mm	Produced weak zone and later bacteria grown.	[12]
	Hormophysa cuneiformis	Methanol	-	-	-	0.1 mL	2-3 mm	-	[16]
	Iyengaria stellata	Methanol	-	-	-	2 µg/mL	12 mm	Species analysed as human/animal pathogens	[10]
	Kappaphycus alvarezii	Aqueous (gel-like liquid)	-	Tannins, flavonoids, alkaloids, steroids and saponins.	-	40 µg/mL	2 mm	-	[17]
		Aqueous (gel-like liquid)	-	Tannins, flavonoids, alkaloids, steroids and saponins.	-	60 µg/mL	4 mm	-	[17]

Pseudomonas aeruginosa	*Kappaphycus alvarezii*	Aqueous (gel-like liquid)	-	Tannins, flavonoids, alkaloids, steroids and saponins.	-	80 µg/mL	7 mm	-	[17]
		Aqueous (gel-like liquid)	-	Tannins, flavonoids, alkaloids, steroids and saponins.	-	100 µg/mL	10 mm	-	[17]
	Melanothamnus afaqhusainii	Ethanol	-	-	-	4 mg/disc	8 mm	Produced weak zone and later bacteria grown.	[12]
		Ethanol	-	-	-	6 mg/disc	8 mm	Produced weak zone and later bacteria grown.	[12]
	Padina gymnospora	Ethyl acetate	-	-	-	1.25 mg/disc	7.33±0.58 mm	-	[8]
		Ethyl acetate	-	-	-	2.5 mg/disc	9.00±0.00 mm	-	[8]
		Ethyl acetate	-	-	-	5 mg/disc	10.33±0.58 mm	-	[8]
		Acetone	-	-	-	1.25 mg/disc	10.67±0.58 mm	-	[8]
		Acetone	-	-	-	2.5 mg/disc	12.67±0.58 mm	-	[8]
		Acetone	-	-	-	5 mg/disc	15.67±0.58 mm	-	[8]
	Padina sp.	Hexane	-	-	-	0.1 mL	2-3 mm	-	[16]
	Ptilophora subcostata	Crude extract	-	-	-	60 µl	>10 mm	-	[18]
	Sargassum desfontainesii	*n*-hexane	Autumn	-	-	5 µL	8 mm	-	[9]
	Sargassum polycystum	Methanol	-	-	-	50 µL	15 mm	-	[30]
	Sargassum sp.	Hexane	-	-	-	0.1 mL	2-3 mm	-	[16]
	Sargassum tenerrimum	Water	-	-	-	-	1 mm	Graphical values	[21]
		Methanol	-	Amino acids, alkaloids, carbohydrates, saponins, sterols, terpenoids, proteins, and phenolic compounds	-	-	13mm	Graphical values	[21]

				(flavonoids and tannins)					
Pseudomonas aeruginosa	*Sargassum tenerrimum*	Aqueous (gel-like liquid)	-	Tannins, flavonoids, alkaloids, steroids, saponins, phlorotannins and terpenoids.	-	40 µg/mL	4 mm	-	[17]
		Aqueous (gel-like liquid)	-	Tannins, flavonoids, alkaloids, steroids, saponins, phlorotannins and terpenoids.	-	60 µg/mL	5 mm	-	[17]
		Aqueous (gel-like liquid)	-	Tannins, flavonoids, alkaloids, steroids, saponins, phlorotannins and terpenoids.	-	80 µg/mL	6 mm	-	[17]
		Aqueous (gel-like liquid)	-	Tannins, flavonoids, alkaloids, steroids, saponins, phlorotannins and terpenoids.	-	100 µg/mL	12 mm	-	[17]
	Sargassum tenerrimum	Methanol	-	Cholest-5-en-3-ol, 24-propylidene-, (3á)-; 1,2-Benzenedicarboxylic acid, diisooctyl ester; Hentriacontane; 1-Docosene; 1-Nonadecene; 1-Hexadecanol; 1,2-	-	Not specified.	17.3±2.3 mm	1,2-benzenedicarboxylic acid, diisooctyl ester, 1-docosene, 1,2-benzenediol and benzoic acid are indicated as the responsible compounds for	[22]

				Benzenediol; Benzoic acid.				the antibacterial activity.	
	Sargassum tenerrimum	Petroleum ether	-	Benzoic acid, 3,5-dicyclohexyl-4-hydroxy-, methyl ester; Isomethadone; Cholesterol; Squalene; 9-Hexadecenoic acid, eicosyl ester, (Z)-; 17-Pentatriacontene; Dasycarpidan-1-methanol, acetate (ester); Hexadecanoic acid, methyl ester.	-	Not specified.	14.3±1.6 mm	Hexadecenoic acid, methyl ester, 17-pentatriacontene, dasycarpian-1-methanol, and acetate are indicated as the responsible compounds for the antibacterial activity.	[22]
	Sargassum wightii	Acetone	-	Steroids, terpenoids, glycosides, alkaloids, flavonoids, tannins and saponins	-	-	12±0.6	-	[24]
		Diethyl ether	-	Steroids, terpenoids, glycosides, flavonoids and saponins	-	-	10±0.2	-	[24]
Pseudomonas aeruginosa	*Sargassum wightii*	Methanol	-	Steroids, terpenoids, alkaloids, flavonoids, tannins and saponins	-	-	14±0.5	-	[24]
		Acetone	-	-	-	1.25	9.67±0.58	-	[8]
		Acetone	-	-	-	2.5	10.67±0.58	-	[8]
		Acetone	-	-	-	5	11.67±0.58	-	[8]

	Stoechospermum sp	Ethanol	-	-	-	50 µl, 75 µl, 100 µl	4 mm		[31]
	Stypopodium zonale	*n*-hexane	Autumn	-	-	5 µL	8 mm	-	[9]
	Turbinaria ornata	Methanol	-	-	-	0.1 mL	2-3 mm	-	[16]
Pseudomonas fluorescens	*Centroceiod* sp.*	Ethanol	-	-	-	51 µl, 75 µl, 100 µl	7 mm	-	[27]
		Ethanol+Chloroform	-	-	-	52 µl, 75 µl, 100 µl	7 mm	-	[27]
	Chaetomorpha sp.	Ethanol	-	-	-	53 µl, 75 µl, 100 µl	5 mm	-	[27]
		Ethanol+Chloroform	-	-	-	54 µl, 75 µl, 100 µl	4 mm	-	[27]
		Methanol	-	-	-	55 µl, 75 µl, 100 µl	6 mm	-	[27]
	Gracilaria corticata	70% methanol	-	Sulfurous acid, 2-ethylhexyl isohexyl ester, hexatriacontane, octacosane, mono (2-ethylhexyl) phthalate, 1-iodo-2-methylundecane and pentatriacontane	-	100 µg/mL	8 ± 0.11 mm	Antibacterial activity associated with fatty acids and suLfurous acid, 2-ethylhexyl isohexyl ester, eµgenol, benzene and phthalic acid	[14]
		70% methanol	-	Sulfurous acid, 2-ethylhexyl isohexyl ester, hexatriacontane, octacosane, mono (2-ethylhexyl) phthalate, 1-iodo-2-methylundecane and pentatriacontane	-	200 µg/mL	10 ± 0.05 mm	Antibacterial activity associated with fatty acids and suLfurous acid, 2-ethylhexyl isohexyl ester, eµgenol, benzene and phthalic acid	[14]
Pseudomonas fluorescens	*Gracilaria corticata*	70% methanol	-	Sulfurous acid, 2-ethylhexyl isohexyl ester, hexatriacontane,	-	300 µg/mL	12 ± 0.07 mm	Antibacterial activity associated with fatty acids and	[14]

				octacosane, mono (2-ethylhexyl) phthalate, 1-iodo-2-methylundecane and pentatriacontane				suLfurous acid, 2-ethylhexyl isohexyl ester, eµgenol, benzene and phthalic acid	
		70% methanol	-	Sulfurous acid, 2-ethylhexyl isohexyl ester, hexatriacontane, octacosane, mono (2-ethylhexyl) phthalate, 1-iodo-2-methylundecane and pentatriacontane	-	400 µg/mL	13 ± 0.03 mm	Antibacterial activity associated with fatty acids and suLfurous acid, 2-ethylhexyl isohexyl ester, eµgenol, benzene and phthalic acid	[14]
		70% methanol	-	Sulfurous acid, 2-ethylhexyl isohexyl ester, hexatriacontane, octacosane, mono (2-ethylhexyl) phthalate, 1-iodo-2-methylundecane and pentatriacontane	-	500 µg/mL	16 ± 0.10 mm	Antibacterial activity associated with fatty acids and suLfurous acid, 2-ethylhexyl isohexyl ester, eµgenol, benzene and phthalic acid	[14]
Pseudomonas fluorescens	*Gracilaria corticata*	DMSO	-	Sulfurous acid, 2-ethylhexyl isohexyl ester, hexatriacontane, octacosane, mono (2-ethylhexyl) phthalate, 1-iodo-2-methylundecane and pentatriacontane	-	100 µg/mL	4 ± 0.05 mm	Antibacterial activity associated with fatty acids and suLfurous acid, 2-ethylhexyl isohexyl ester, eµgenol, benzene and phthalic acid	[14]

		DMSO	-	Sulfurous acid, 2-ethylhexyl isohexyl ester, hexatriacontane, octacosane, mono (2-ethylhexyl) phthalate, 1-iodo-2-methylundecane and pentatriacontane	-	200 µg/mL	7 ± 0.20 mm	Antibacterial activity associated with fatty acids and suLfurous acid, 2-ethylhexyl isohexyl ester, eμgenol, benzene and phthalic acid	[14]
		DMSO	-	Sulfurous acid, 2-ethylhexyl isohexyl ester, hexatriacontane, octacosane, mono (2-ethylhexyl) phthalate, 1-iodo-2-methylundecane and pentatriacontane	-	300 µg/mL	9 ± 0.01 mm	Antibacterial activity associated with fatty acids and suLfurous acid, 2-ethylhexyl isohexyl ester, eμgenol, benzene and phthalic acid	[14]
		DMSO	-	Sulfurous acid, 2-ethylhexyl isohexyl ester, hexatriacontane, octacosane, mono (2-ethylhexyl) phthalate, 1-iodo-2-methylundecane and pentatriacontane	-	400 µg/mL	11 ± 0.16 mm	Antibacterial activity associated with fatty acids and suLfurous acid, 2-ethylhexyl isohexyl ester, eμgenol, benzene and phthalic acid	[14]
Pseudomonas fluorescens	*Gracilaria corticata*	DMSO	-	Sulfurous acid, 2-ethylhexyl isohexyl ester, hexatriacontane, octacosane, mono (2-ethylhexyl) phthalate, 1-iodo-2-	-	500 µg/mL	13 ± 0.01 mm	Antibacterial activity associated with fatty acids and suLfurous acid, 2-ethylhexyl isohexyl ester, eμgenol,	[14]

				methylundecane and pentatriacontane				benzene and phthalic acid	
	Hydropuntia edulis	70% methanol	-	Eugenol, nonane, undecane, hept-2-ene, 2,4,4,6-tetramethyl, sulfurous acid, phthalic acid and 1,2-propanediol	-	100 µg/mL	3 ± 0.05 mm	Antibacterial activity associated with fatty acids and suLfurous acid, 2-ethylhexyl isohexyl ester, eµgenol, benzene and phthalic acid	[14]
		70% methanol	-	Eugenol, nonane, undecane, hept-2-ene, 2,4,4,6-tetramethyl, sulfurous acid, phthalic acid and 1,2-propanediol	-	200 µg/mL	3 ± 0.01 mm	Antibacterial activity associated with fatty acids and suLfurous acid, 2-ethylhexyl isohexyl ester, eµgenol, benzene and phthalic acid	[14]
		70% methanol	-	Eugenol, nonane, undecane, hept-2-ene, 2,4,4,6-tetramethyl, sulfurous acid, phthalic acid and 1,2-propanediol	-	300 µg/mL	4 ± 0.11 mm	Antibacterial activity associated with fatty acids and suLfurous acid, 2-ethylhexyl isohexyl ester, eµgenol, benzene and phthalic acid	[14]
Pseudomonas fluorescens	*Hydropuntia edulis*	70% methanol	-	Eugenol, nonane, undecane, hept-2-ene, 2,4,4,6-tetramethyl, sulfurous acid, phthalic acid and 1,2-propanediol	-	400 µg/mL	5 ± 0.20 mm	Antibacterial activity associated with fatty acids and suLfurous acid, 2-ethylhexyl isohexyl ester, eµgenol,	[14]

								benzene and phthalic acid	
		70% methanol	-	Eugenol, nonane, undecane, hept-2-ene, 2,4,4,6-tetramethyl, sulfurous acid, phthalic acid and 1,2-propanediol	-	500 µg/mL	6 ± 0.06 mm	Antibacterial activity associated with fatty acids and suLfurous acid, 2-ethylhexyl isohexyl ester, eµgenol, benzene and phthalic acid	[14]
		DMSO	-	Eugenol, nonane, undecane, hept-2-ene, 2,4,4,6-tetramethyl, sulfurous acid, phthalic acid and 1,2-propanediol	-	100 µg/mL	4 ± 0.10 mm	Antibacterial activity associated with fatty acids and suLfurous acid, 2-ethylhexyl isohexyl ester, eµgenol, benzene and phthalic acid	[14]
		DMSO	-	Eugenol, nonane, undecane, hept-2-ene, 2,4,4,6-tetramethyl, sulfurous acid, phthalic acid and 1,2-propanediol	-	200 µg/mL	5 ± 0.02 mm	Antibacterial activity associated with fatty acids and suLfurous acid, 2-ethylhexyl isohexyl ester, eµgenol, benzene and phthalic acid	[14]
Pseudomonas fluorescens	*Hydropuntia edulis*	DMSO	-	Eugenol, nonane, undecane, hept-2-ene, 2,4,4,6-tetramethyl, sulfurous acid, phthalic acid and 1,2-propanediol	-	300 µg/mL	6 ± 0.05 mm	Antibacterial activity associated with fatty acids and suLfurous acid, 2-ethylhexyl isohexyl ester, eµgenol,	[14]

								benzene and phthalic acid	
		DMSO	-	Eugenol, nonane, undecane, hept-2-ene, 2,4,4,6-tetramethyl, sulfurous acid, phthalic acid and 1,2-propanediol	-	400 µg/mL	8 ± 0.20 mm	Antibacterial activity associated with fatty acids and suLfurous acid, 2-ethylhexyl isohexyl ester, eµgenol, benzene and phthalic acid	[14]
		DMSO	-	Eugenol, nonane, undecane, hept-2-ene, 2,4,4,6-tetramethyl, sulfurous acid, phthalic acid and 1,2-propanediol	-	500 µg/mL	9 ± 0.11 mm	Antibacterial activity associated with fatty acids and suLfurous acid, 2-ethylhexyl isohexyl ester, eµgenol, benzene and phthalic acid	[14]
	Stoechospermum sp	Ethanol	-	-	-	50 µl, 75 µl, 100 µl	5 mm	-	[31]
		Ethanol:chloroform (1:1)	-	-	-	50 µl, 75 µl, 100 µl	4 mm	-	[31]
	Ulva sp.	Ethanol	-	-	-	56 µl, 75 µl, 100 µl	8 mm	-	[27]
Pseudomonas sp.	*Gracilaria corticata*	Ethanol	-	Abundance of alcohols and phenols	-	25 µL (holding capacity)	9±0 mm	-	[32]
Pseudomonas sp.	*Gracilaria corticata*	Fraction 1 (Chloroform:methanol)	-	-	-	25 µL (holding capacity)	4 ± 0.42 mm	-	[32]
		Fraction 2 (Chloroform:methanol)	-	-	-	25 µL (holding capacity)	6 ± 0.17 mm	-	[32]
		Fraction 3 (Chloroform:methanol)	-	-	-	25 µL (holding capacity)	5 ± 1.63 mm	-	[32]

	Ulva lactuca	Ethanol (crude)	-	Abundance of alcohols and phenols	-	25 µL (holding capacity)	6 ± 2.44 mm	-	[32]
		Fraction 1 (Chloroform:methanol)	-	-	-	25 µL (holding capacity)	5 ± 0.81 mm	-	[32]
		Fraction 2 (Chloroform:methanol)	-	-	-	25 µL (holding capacity)	5 ± 0.81 mm	-	[32]
		Fraction 3 (Chloroform:methanol)	-	-	-	25 µL (holding capacity)	6 ± 1.63 mm	-	[32]
Staphylococcus aureus	*Acanthophora spicifera*	Ethanol	-	Flavonoids and tannins	-	-	6.3 ± 0.5 mm	-	[33]
	Aglaothamnion sepositum	-	Spring	-	-	Disc thallus	3 mm	-	[2]
		-	Summer	-	-	Disc thallus	2,5 mm	-	[2]
		-	Autumn	-	-	Disc thallus	2,5 mm	-	[2]
		-	Winter	-	-	Disc thallus	2 mm	-	[2]
	Alaria esculenta	-	Summer	-	-	Disc thallus	4 mm	-	[2]
		-	Autumn	-	-	Disc thallus	6 mm	-	[2]
		-	Summer (Sterile)	-	-	Disc thallus	4 mm	-	[2]
	Antithamnion cruciatum	-	Spring	-	-	Disc thallus	4 mm	-	[2]
		-	Summer	-	-	Disc thallus	2,5 mm	-	[2]
		-	Autumn	-	-	Disc thallus	1 mm	-	[2]
		-	Winter	-	-	Disc thallus	1 mm	-	[2]
	Asparagopsis armata	-	Summer	-	-	Disc thallus	12 mm (width of inhibition zone)	-	[2]
	Avrainvillea nigricans	Ethyl acetate partition of Methanols	-	-	-	100 µL	0.38	Data of inhibition not specified (units)	[4]
Staphylococcus aureus	*Bangia fuscopurpurea*	Chloroform	-	-	Fatty acids (free or as acylglycerols)	-	-	-	[34]
	Bifurcaria bifurcata	-	Spring	-	-	Disc thallus	3,5 mm	-	[2]
		-	Summer	-	-	Disc thallus	4 mm	-	[2]
		-	Autumn	-	-	Disc thallus	4 mm	-	[2]

	Bonnemaisonia asparagoides	-	-	E,Z-1-bromo-1,2-dichloro-1-octene-3-one	-	-	-	It presents antibacterial activity	[35]
		-	-	E,Z-1-bromo-1,2,2-trichloro-1-octene-3-one	-	-	-	It presents antibacterial activity	[35]
		-	-	2,4-dibromo-1,1-dichloro-1-octene-3-one	-	-	-	It presents antibacterial activity	[35]
Staphylococcus aureus	*Bonnemaisonia asparagoides*	-	-	E-1,2-dibromo-1,4-dichloro-1-octene-3-one	-	-	-	It presents antibacterial activity	[35]
		-	-	E-1-chloro-1,2,4-tribromo-1-octene-3-one	-	-	-	It presents antibacterial activity	[35]
		-	Summer	-	-	Disc thallus	16 mm	-	[2]
	Bonnemaisonia hamifera	-	Summer	-	-	Disc thallus	17 mm	-	[2]
		-	Summer (Sterile)	-	-	Disc thallus	17 mm	-	[2]
		-	Spring (Tetrasporic)	-	-	Disc thallus	29 mm	-	[2]
	Bostrychia scorpioides	-	Spring	-	-	Disc thallus	3 mm	-	[2]
		-	Summer	-	-	Disc thallus	1 mm	-	[2]
		-	Autumn	-	-	Disc thallus	1 mm	-	[2]
		-	Winter	-	-	Disc thallus	2 mm	-	[2]
	Bryopsis plumosa	-	Spring	-	-	Disc thallus	4 mm	-	[2]
		-	Summer	-	-	Disc thallus	1,5 mm	-	[2]
	Callithamnion tetragonum	-	Spring	-	-	Disc thallus	5,5 mm	-	[2]
		-	Summer	-	-	Disc thallus	5 mm	-	[2]
		-	Autumn	-	-	Disc thallus	5 mm	-	[2]
		-	Winter	-	-	Disc thallus	4 mm	-	[2]
	Callithamnion tetricum	-	Spring	-	-	Disc thallus	5 mm	-	[2]
		-	Summer	-	-	Disc thallus	7 mm	-	[2]
		-	Autumn	-	-	Disc thallus	6 mm	-	[2]
		-	Winter	-	-	Disc thallus	4 mm	-	[2]
	Callocolax neglectus	-	Summer	-	-	Disc thallus	2 mm	-	[2]
		-	Autumn	-	-	Disc thallus	1 mm	-	[2]
		-	Spring	-	-	Disc thallus	15,5 mm	-	[2]

	Carradoriella elongata	-	Summer	-	-	Disc thallus	16 mm	-	[2]
		-	Autumn	-	-	Disc thallus	16 mm	-	[2]
		-	Winter	-	-	Disc thallus	12 mm	-	[2]
		-	Spring (Sterile)	-	-	Disc thallus	16 mm	-	[2]
		-	Spring (Tetrasporic)	-	-	Disc thallus	15 mm	-	[2]
	Caulerpa peltata	Ethanol	-	Abundance of alcohols and phenols	-	-	6.6 ± 1.5 mm	-	[33]
Staphylococcus aureus	*Caulerpa scalpelliformis*	Ethanol	-	Abundance of alcohols and phenols	-	-	6.0 ± 1.0 mm	-	[33]
	Chaetomorpha antennina	Petroleum ether	-	-	-	50 µg/mL (dissolved in DMSO)	7.3 ± 0.8 mm	-	[7]
		Petroleum ether	-	-	-	100 µg/mL (dissolved in DMSO)	7.83 ± 1.8 mm	-	[7]
		Petroleum ether	-	-	-	200 µg/mL (dissolved in DMSO)	11 ± 0.6 mm	-	[7]
		Petroleum ether	-	-	-	300 µg/mL (dissolved in DMSO)	12.8 ± 0.7 mm	-	[7]
		Petroleum ether	-	-	-	400 µg/mL (dissolved in DMSO)	14.8 ± 0.7 mm	-	[7]
		Petroleum ether	-	-	-	500 µg/mL (dissolved in DMSO)	18 ± 2.4 mm	-	[7]
	Chondria dasyphylla	-	Spring	-	-	Disc thallus	4 mm	-	[2]
		-	Summer	-	-	Disc thallus	1,5 mm	-	[2]
		-	Autumn	-	-	Disc thallus	2,5 mm	-	[2]
		-	Winter	-	-	Disc thallus	2,5 mm	-	[2]
	Chondrus crispus	-	Spring	-	-	Disc thallus	14 mm	-	[2]
		-	Summer	-	-	Disc thallus	15,5 mm	-	[2]
		-	Autumn	-	-	Disc thallus	15 mm	-	[2]
		-	Winter	-	-	Disc thallus	22 mm	-	[2]

		-	Spring (Sterile)	-	-	Disc thallus	14 mm	-	[2]
		-	Spring (Tetrasporic)	-	-	Disc thallus	15 mm	-	[2]
	Chordaria flagelliformis	-	Summer	-	-	Disc thallus	4 mm	-	[2]
		-	Autumn	-	-	Disc thallus	3,5 mm	-	[2]
	Codium decorticatum	Ethyl acetate	-	-	-	100 µL	0.50	Data of inhibition not specified (units)	[4]
	Codium fragile	-	Spring	-	-	Disc thallus	7 mm	-	[2]
		-	Summer	-	-	Disc thallus	6 mm	-	[2]
Staphylococcus aureus	*Codium fragile*	-	Autumn	-	-	Disc thallus	4 mm	-	[2]
		-	Winter	-	-	Disc thallus	4 mm	-	[2]
	Codium intertextum	Methanol	Autumn	-	-	5 µL	10 mm	-	[9]
		Methanol	Winter	-	-	5 µL	10 mm	-	[9]
	Codium tomentosum	-	Spring	-	-	Disc thallus	6 mm	-	[2]
		-	Summer	-	-	Disc thallus	5,5 mm	-	[2]
		-	Autumn	-	-	Disc thallus	4,5 mm	-	[2]
		-	Winter	-	-	Disc thallus	4 mm	-	[2]
	Crassiphycus changii	Diethyl ether	Cholesteryl myristate	-	-	0.2 mg/disc	12.6 ±0.6 mm	-	[11]
	Delesseria sanguinea	-	Spring	-	-	Disc thallus	3,5 mm	-	[2]
		-	Summer	-	-	Disc thallus	1 mm	-	[2]
		-	Autumn	-	-	Disc thallus	1 mm	-	[2]
	Desmarestia aculeata	-	Spring	-	-	Disc thallus	12 mm	-	[2]
		-	Summer	-	-	Disc thallus	10 mm	-	[2]
		-	Autumn	-	-	Disc thallus	9 mm	-	[2]
		-	Winter	-	-	Disc thallus	4 mm	-	[2]
	Desmarestia ligulata	-	Summer	-	-	Disc thallus	27 mm	-	[2]
		-	Autumn	-	-	Disc thallus	26 mm	-	[2]
	Dictyopteris polypodioides	-	Spring	-	-	Disc thallus	6,5 mm	-	[2]
		-	Summer	-	-	Disc thallus	7 mm	-	[2]
		-	Autumn	-	-	Disc thallus	7 mm	-	[2]
		-	Winter	-	-	Disc thallus	3 mm	-	[2]

		Methanol	-	-	Phlorotannins present in brown algae	-	28 mm	-	[28]
	Dictyota cervicornis	Ethanol	-	-	-	6 mg/disc	9 mm	-	[12]
	Dictyota dichotoma	-	Spring	-	-	Disc thallus	2 mm	-	[2]
		-	Summer	-	-	Disc thallus	8 mm	-	[2]
		-	Autumn	-	-	Disc thallus	3 mm	-	[2]
		-	Summer (Sterile)	-	-	Disc thallus	8 mm	-	[36]
		-	Summer (Male)	-	-	Disc thallus	8,5 mm	-	[36]
		-	Summer (Female)	-	-	Disc thallus	9 mm	-	[36]
		Methanol	-	-	-	25 µL	0-5 mm	-	[37]
	Dictyota dichotoma	Diethyl ether	-	-	-	25 µL	0-5 mm	-	[37]
		Chloroform	-	-	-	25 µL	5-10 mm	-	[37]
Staphylococcus aureus	*Dictyota dichotoma* var *intricata*	Ethanol	-	-	-	2 mg/disc	9 mm	-	[12]
		Ethanol	-	-	-	4 mg/disc	10 mm	-	[12]
		Ethanol	-	-	-	6 mg/disc	13 mm	-	[12]
	Dilsea carnosa	-	Spring	-	-	Disc thallus	12,5 mm	-	[2]
		-	Summer	-	-	Disc thallus	12 mm	-	[2]
		-	Autumn	-	-	Disc thallus	10 mm	-	[2]
		-	Winter	-	-	Disc thallus	8,5 mm	-	[2]
	Ellisolandia elongata	Methanol	-	-	-	25 µL	5-10 mm	-	[37]
		Diethyl ether	-	-	-	25 µL	0-5 mm	-	[37]
		Chloroform	-	-	-	25 µL	5-10 mm	-	[37]
	*Enteromorpha antenna**	Methanol	-	-	-	250 µg/mL (extract in DMSO)	10 mm	Antibacterial activity in algae reported in bromophenols, carbonyls, halogenated aliphatic compounds, terpenes, isoprenylated and brominated hydroquinones,	[13]

								as well as phlorotannins	
		Methanol	-	-	-	500 µg/mL (extract in DMSO)	13 mm	Antibacterial activity in algae reported in bromophenols, carbonyls, halogenated aliphatic compounds, terpenes, isoprenylated and brominated hydroquinones, as well as phlorotannins	[13]
		Methanol	-	-	-	750 µg/mL (extract in DMSO)	15 mm	Antibacterial activity in algae reported in bromophenols, carbonyls, halogenated aliphatic compounds, terpenes, isoprenylated and brominated hydroquinones, as well as phlorotannins	[13]
		Methanol	-	-	-	1000 µg/mL (extract in DMSO)	17 mm	Antibacterial activity in algae reported in bromophenols, carbonyls, halogenated aliphatic compounds, terpenes, isoprenylated and brominated	[13]

								hydroquinones, as well as phlorotannins	
	Ericaria selaginoides	-	Spring	-	-	Disc thallus	5 mm	-	[2]
		-	Summer	-	-	Disc thallus	5 mm	-	[2]
		-	Autumn	-	-	Disc thallus	4 mm	-	[2]
		-	Winter	-	-	Disc thallus	2,5 mm	-	[2]
	Eudesme virescens	-	Summer	-	-	Disc thallus	3 mm	-	[2]
		-	Autumn	-	-	Disc thallus	3,5 mm	-	[2]
	Gloiosiphonia capillaris	-	Summer	-	-	Disc thallus	36 mm	-	[2]
	Gongolaria baccata	-	Spring	-	-	Disc thallus	4 mm	-	[2]
		-	Summer	-	-	Disc thallus	4,5 mm	-	[2]
		-	Autumn	-	-	Disc thallus	4,5 mm	-	[2]
		-	Winter	-	-	Disc thallus	2 mm	-	[2]
	Gracilaria corticata	Ethanol		Abundance of alcohols and phenols	-	25 µL (holding capacity)	6 ± 3.48 mm	-	[32]
		Fraction 1 (Chloroform:met hanol)	-	-	-	25 µL (holding capacity)	4 ± 0.50 mm	-	[32]
		Fraction 2 (Chloroform:met hanol)	-	-	-	25 µL (holding capacity)	4 ± 0.35 mm	-	[32]
		Fraction 3 (Chloroform:met hanol)	-	-	-	25 µL (holding capacity)	5 ± 0.82 mm	-	[32]
	Gracilaria corticata	Methanol	-	-	-	500 µg/mL (extract in DMSO)	10 mm	Antibacterial activity reported in bromophenols, carbonyls, halogenated aliphatic compounds, terpenes, isoprenylated and brominated hydroquinones,	[13]

								as well as phlorotannins	
Staphylococcus aureus		Methanol	-	-	-	750 µg/mL (extract in DMSO)	12 mm	Antibacterial activity reported in bromophenols, carbonyls, halogenated aliphatic compounds, terpenes, isoprenylated and brominated hydroquinones, as well as phlorotannins	[13]
		Methanol	-	-	-	1000 µg/mL (extract in DMSO)	14 mm	Antibacterial activity reported in bromophenols, carbonyls, halogenated aliphatic compounds, terpenes, isoprenylated and brominated hydroquinones, as well as phlorotannins	[13]
	Gracilaria corticata	70% methanol	-	Sulfurous acid, 2-ethylhexyl isohexyl ester, hexatriacontane, octacosane, mono (2-ethylhexyl) phthalate, 1-iodo-2-methylundecane and pentatriacontane	-	100 µg/mL	4 ± 0.10 mm	Antibacterial activity associated with fatty acids and suLfurous acid, 2-ethylhexyl isohexyl ester, eµgenol, benzene and phthalic acid	[14]

Staphylococcus aureus		70% methanol	-	Sulfurous acid, 2-ethylhexyl isohexyl ester, hexatriacontane, octacosane, mono (2-ethylhexyl) phthalate, 1-iodo-2-methylundecane and pentatriacontane	-	200 µg/mL	8 ± 0.04 mm	Antibacterial activity associated with fatty acids and suLfurous acid, 2-ethylhexyl isohexyl ester, eµgenol, benzene and phthalic acid	[14]
		70% methanol	-	Sulfurous acid, 2-ethylhexyl isohexyl ester, hexatriacontane, octacosane, mono (2-ethylhexyl) phthalate, 1-iodo-2-methylundecane and pentatriacontane	-	300 µg/mL	12 ± 0.00 mm	Antibacterial activity associated with fatty acids and suLfurous acid, 2-ethylhexyl isohexyl ester, eµgenol, benzene and phthalic acid	[14]
		70% methanol	-	Sulfurous acid, 2-ethylhexyl isohexyl ester, hexatriacontane, octacosane, mono (2-ethylhexyl) phthalate, 1-iodo-2-methylundecane and pentatriacontane	-	400 µg/mL	14 ± 0.20 mm	Antibacterial activity associated with fatty acids and suLfurous acid, 2-ethylhexyl isohexyl ester, eµgenol, benzene and phthalic acid	[14]
Staphylococcus aureus	*Gracilaria corticata*	70% methanol	-	Sulfurous acid, 2-ethylhexyl isohexyl ester, hexatriacontane, octacosane, mono (2-ethylhexyl) phthalate, 1-iodo-2-	-	500 µg/mL	15 ± 0.16 mm	Antibacterial activity associated with fatty acids and suLfurous acid, 2-ethylhexyl isohexyl ester, eµgenol,	[14]

				methylundecane and pentatriacontane				benzene and phthalic acid	
		DMSO	-	Sulfurous acid, 2-ethylhexyl isohexyl ester, hexatriacontane, octacosane, mono (2-ethylhexyl) phthalate, 1-iodo-2-methylundecane and pentatriacontane	-	100 µg/mL	6 ± 0.05 mm	Antibacterial activity associated with fatty acids and suLfurous acid, 2-ethylhexyl isohexyl ester, eµgenol, benzene and phthalic acid	[14]
		DMSO	-	Sulfurous acid, 2-ethylhexyl isohexyl ester, hexatriacontane, octacosane, mono (2-ethylhexyl) phthalate, 1-iodo-2-methylundecane and pentatriacontane	-	200 µg/mL	8 ± 0.02 mm	Antibacterial activity associated with fatty acids and suLfurous acid, 2-ethylhexyl isohexyl ester, eµgenol, benzene and phthalic acid	[14]
		DMSO	-	Sulfurous acid, 2-ethylhexyl isohexyl ester, hexatriacontane, octacosane, mono (2-ethylhexyl) phthalate, 1-iodo-2-methylundecane and pentatriacontane	-	300 µg/mL	10 ± 0.08 mm	Antibacterial activity associated with fatty acids and suLfurous acid, 2-ethylhexyl isohexyl ester, eµgenol, benzene and phthalic acid	[14]
Staphylococcus aureus	*Gracilaria corticata*	DMSO	-	Sulfurous acid, 2-ethylhexyl isohexyl ester, hexatriacontane, octacosane, mono	-	400 µg/mL	12 ± 0.01 mm	Antibacterial activity associated with fatty acids and suLfurous acid,	[14]

				(2-ethylhexyl) phthalate, 1-iodo-2-methylundecane and pentatriacontane				2-ethylhexyl isohexyl ester, eμgenol, benzene and phthalic acid	
		DMSO	-	Sulfurous acid, 2-ethylhexyl isohexyl ester, hexatriacontane, octacosane, mono (2-ethylhexyl) phthalate, 1-iodo-2-methylundecane and pentatriacontane	-	500 μg/mL	14 ± 0.04 mm	Antibacterial activity associated with fatty acids and suLfurous acid, 2-ethylhexyl isohexyl ester, eμgenol, benzene and phthalic acid	[14]
		Chloroform (Soxhlet/hot)	-	Alkaloids, steroids, sugars	Saponins, alkaloids, phenols, steroids and triterpenoids and free hydroxyl groups	125 μg/ml	9 mm	Tannins at low concentration and saponins have antimicrobial activity	[29]
	Gracilaria corticata	Isopropanol (Soxhlet/hot)	-	Phenols, steroids, glycosides	Saponins, alkaloids, phenols, steroids and triterpenoids and free hydroxyl groups	125 μg/ml	9 mm	Tannins at low concentration have antimicrobial activity.	[29]
Staphylococcus aureus		Chloroform (Cold)	-	Alkaloids, saponins, steroids, sugars	Saponins, alkaloids, phenols, steroids and triterpenoids and free hydroxyl groups	125 μg/ml	8 mm	Tannins at low concentration and saponins have antimicrobial activity	[29]
		Isopropanol (Cold)	-	Phenols, glycosides, sugars	Saponins, alkaloids, phenols, steroids and triterpenoids and free hydroxyl groups	125 μg/ml	6 mm	Tannins at low concentration have antimicrobial activity.	[29]

		Petroleum ether (Cold)	-	Alkaloids, saponins, steroids, tannins, sugars	Saponins, alkaloids, phenols, steroids and triterpenoids and free hydroxyl groups	125 µg/ml	7 mm	Tannins at low concentration and saponins have antimicrobial activity	[29]
Staphylococcus aureus	*Gracilaria corticata*	Ethanol	-	Flavonoids and tannins	-	-	7.3 ± 1.1 mm	-	[33]
	Gracilariopsis longissima	-	Autumn	-	-	Disc thallus	1 mm	-	[2]
		-	Winter	-	-	Disc thallus	4 mm	-	[2]
		-	Winter (Sterile)	-	-	Disc thallus	4 mm	-	[2]
		-	Winter (Cystocarpic)	-	-	Disc thallus	6 mm	-	[2]
	Halidrys siliquosa	-	Spring	-	-	Disc thallus	6,5 mm	-	[2]
		-	Summer	-	-	Disc thallus	5 mm	-	[2]
		-	Autumn	-	-	Disc thallus	4 mm	-	[2]
		-	Winter	-	-	Disc thallus	2,5 mm	-	[2]
		-	Winter (Sterile)	-	-	Disc thallus	3 mm	-	[36]
		-	Winter (Fertile)	-	-	Disc thallus	2,5 mm	-	[36]
	Haligra sps.*	Methanol	-	High phenolic content	-	200 µg (1 mg/ml)	16 ± 0.9 mm	-	[38]
	Halimeda tuna	Ethanol	-	-	-	4 mg/disc	7 mm	Produced weak zone and later bacteria grown.	[12]
		Ethanol	-	-	-	6 mg/disc	9 mm	-	[12]
	Halopithys incurva	-	Spring	-	-	Disc thallus	4 mm	-	[2]
		-	Summer	-	-	Disc thallus	2,5 mm	-	[2]
		-	Autumn	-	-	Disc thallus	2 mm	-	[2]
		-	Winter	-	-	Disc thallus	2 mm	-	[2]
	Halopterois scoparia	Methanol	Autumn	-	-	5 µL	10 mm	-	[9]
		Methanol	Winter	-	-	5 µL	15 mm	-	[9]
	Hydropuntia edulis	70% methanol	-	Eugenol, nonane, undecane, hept-2-ene, 2,4,4,6-tetramethyl, sulfurous acid,	-	100 µg/mL	3 ± 0.05 mm	Antibacterial activity associated with fatty acids and suLfurous acid, 2-ethylhexyl	[14]

				phthalic acid and 1,2-propanediol				isohexyl ester, eugenol, benzene and phthalic acid	
		70% methanol	-	Eugenol, nonane, undecane, hept-2-ene, 2,4,4,6-tetramethyl, sulfurous acid, phthalic acid and 1,2-propanediol	-	200 µg/mL	3 ± 0.11 mm	Antibacterial activity associated with fatty acids and suLfurous acid, 2-ethylhexyl isohexyl ester, eugenol, benzene and phthalic acid	[14]
	Hydropuntia edulis	70% methanol	-	Eugenol, nonane, undecane, hept-2-ene, 2,4,4,6-tetramethyl, sulfurous acid, phthalic acid and 1,2-propanediol	-	300 µg/mL	4 ± 0.03 mm	Antibacterial activity associated with fatty acids and suLfurous acid, 2-ethylhexyl isohexyl ester, eugenol, benzene and phthalic acid	[14]
Staphylococcus aureus	*Hydropuntia edulis*	70% methanol	-	Eugenol, nonane, undecane, hept-2-ene, 2,4,4,6-tetramethyl, sulfurous acid, phthalic acid and 1,2-propanediol	-	400 µg/mL	5 ± 0.06 mm	Antibacterial activity associated with fatty acids and suLfurous acid, 2-ethylhexyl isohexyl ester, eugenol, benzene and phthalic acid	[14]
		70% methanol	-	Eugenol, nonane, undecane, hept-2-ene, 2,4,4,6-tetramethyl, sulfurous acid, phthalic acid and 1,2-propanediol	-	500 µg/mL	6 ± 0.01 mm	Antibacterial activity associated with fatty acids and suLfurous acid, 2-ethylhexyl isohexyl ester,	[14]

								eugenol, benzene and phthalic acid	
		DMSO	-	Eugenol, nonane, undecane, hept-2-ene, 2,4,4,6-tetramethyl, sulfurous acid, phthalic acid and 1,2-propanediol	-	100 µg/mL	3 ± 0.00 mm	Antibacterial activity associated with fatty acids and suLfurous acid, 2-ethylhexyl isohexyl ester, eugenol, benzene and phthalic acid	[14]
		DMSO	-	Eugenol, nonane, undecane, hept-2-ene, 2,4,4,6-tetramethyl, sulfurous acid, phthalic acid and 1,2-propanediol	-	200 µg/mL	4.5 ± 0.11 mm	Antibacterial activity associated with fatty acids and suLfurous acid, 2-ethylhexyl isohexyl ester, eugenol, benzene and phthalic acid	[14]
Staphylococcus aureus	*Hydropuntia edulis*	DMSO	-	Eugenol, nonane, undecane, hept-2-ene, 2,4,4,6-tetramethyl, sulfurous acid, phthalic acid and 1,2-propanediol	-	300 µg/mL	5 ± 0.01	Antibacterial activity associated with fatty acids and suLfurous acid, 2-ethylhexyl isohexyl ester, eugenol, benzene and phthalic acid	[14]
		DMSO	-	Eugenol, nonane, undecane, hept-2-ene, 2,4,4,6-tetramethyl, sulfurous acid, phthalic acid and 1,2-propanediol	-	400 µg/mL	5 ± 0.00	Antibacterial activity associated with fatty acids and suLfurous acid, 2-ethylhexyl isohexyl ester, eugenol,	[14]

								benzene and phthalic acid	
		DMSO	-	Eugenol, nonane, undecane, hept-2-ene, 2,4,4,6-tetramethyl, sulfurous acid, phthalic acid and 1,2-propanediol	-	500 µg/mL	6.5 ± 0.06	Antibacterial activity associated with fatty acids and suLfurous acid, 2-ethylhexyl isohexyl ester, eugenol, benzene and phthalic acid	[14]
	Kappaphycus alvarezii	Aqueous (gel-like liquid)	-	Tannins, flavonoids, alkaloids, steroids and saponins.	-	40 µg/mL	4 mm	-	[17]
		Aqueous (gel-like liquid)	-	Tannins, flavonoids, alkaloids, steroids and saponins.	-	60 µg/mL	2 mm	-	[17]
Staphylococcus aureus	*Kappaphycus alvarezii*	Aqueous (gel-like liquid)	-	Tannins, flavonoids, alkaloids, steroids and saponins.	-	80 µg/mL	4 mm	-	[17]
		Aqueous (gel-like liquid)	-	Tannins, flavonoids, alkaloids, steroids and saponins.	-	100 µg/mL	5 mm	-	[17]
	Laminaria digitata	-	Spring	-	-	Disc thallus	9 mm	-	[2]
		-	Summer	-	-	Disc thallus	7,5 mm	-	[2]
		-	Autumn	-	-	Disc thallus	6,5 mm	-	[2]
		-	Winter	-	-	Disc thallus	16 mm	-	[2]
		-	Spring (Sterile)	-	-	Disc thallus	9 mm	-	[36]
		-	Spring (Fertile)	-	-	Disc thallus	10 mm	-	[36]
	Laurencia obtusa	-	Spring	-	-	Disc thallus	6,5 mm	-	[2]
		-	Summer	-	-	Disc thallus	7 mm	-	[2]
		-	Autumn	-	-	Disc thallus	7 mm	-	[2]
		-	Winter	-	-	Disc thallus	7 mm	-	[2]

		Ethyl acetate	-	-	-	100 µL	0.48	Data of inhibition not specified	[4]
	Lychaete pellucida	-	Spring	-	-	Disc thallus	0,5 mm	-	[2]
		-	Summer	-	-	Disc thallus	2 mm	-	[2]
		-	Autumn	-	-	Disc thallus	3 mm	-	[2]
	Melanothamnus afaqhusainii	Ethanol	-	-	-	2 mg/disc	8 mm	-	[12]
		Ethanol	-	-	-	4 mg/disc	9 mm	-	[12]
		Ethanol	-	-	-	6 mg/disc	10 mm	-	[12]
	Membranoptera alata	-	Spring	-	-	Disc thallus	3 mm	-	[2]
		-	Summer	-	-	Disc thallus	2 mm	-	[2]
	Mesogloia vermiculata	-	Summer	-	-	Disc thallus	3 mm	-	[2]
	Odonthalia dentata	-	Spring	-	-	Disc thallus	15 mm	-	[2]
		-	Summer	-	-	Disc thallus	14,5 mm	-	[2]
		-	Autumn	-	-	Disc thallus	16 mm	-	[2]
		-	Winter	-	-	Disc thallus	16 mm	-	[2]
Staphylococcus aureus	*Osmundea hybrida*	-	Spring	-	-	Disc thallus	7 mm	-	[2]
		-	Summer	-	-	Disc thallus	6,5 mm	-	[2]
		-	Autumn	-	-	Disc thallus	6,5 mm	-	[2]
		-	Winter	-	-	Disc thallus	7 mm	-	[2]
	Osmundea pinnatifida	-	Spring	-	-	Disc thallus	10 mm	-	[2]
		-	Summer	-	-	Disc thallus	9 mm	-	[2]
		-	Autumn	-	-	Disc thallus	10 mm	-	[2]
		-	Winter	-	-	Disc thallus	11 mm	-	[2]
	Padina gymnospora	Methanol	-	Hydroxyl groups (O-H), alkyl group (C-H), (C=O), amine groups	C-H groups can be from mannuronic groups and gluronic	-	18.4 mm	The presence of reactive functional groups of N-H, N=O, O-H and C-O-S (protein, alkaloids and carbohydrates) are associated with microbial cell disrupting	[39]

	Padina sp.	Hexane	-	-	-	0.1 mL	4-7 mm	-	[16]
	Padina tetrastromatica	Ethanol	-	Flavonoids and tannins	-	-	6.33 ± 0.5	-	[33]
	Petrospongium berkeleyi	-	Summer	-	-	Disc thallus	2 mm	-	[2]
		-	Autumn	-	-	Disc thallus	3 mm	-	[2]
	Phyllophora crispa	-	Spring	-	-	Disc thallus	2,5 mm	-	[2]
	Phyllophora pseudoceranoïdes	-	Spring	-	-	Disc thallus	4 mm	-	[2]
		-	Summer	-	-	Disc thallus	1,5 mm	-	[2]
	Plocamium cartilagineum	-	Summer (Cystocarpic)	-	-	Disc thallus	4 mm	-	[36]
	Polysiphonia stricta	-	Spring	-	-	Disc thallus	10,5 mm	-	[2]
		-	Summer	-	-	Disc thallus	10 mm	-	[2]
		-	Autumn	-	-	Disc thallus	10,5 mm	-	[2]
		-	Winter	-	-	Disc thallus	10,5 mm	-	[2]
	Pterothamnion plumula	-	Spring	-	-	Disc thallus	5 mm	-	[2]
		-	Summer	-	-	Disc thallus	3 mm	-	[2]
		-	Autumn	-	-	Disc thallus	3 mm	-	[2]
		-	Winter	-	-	Disc thallus	1 mm	-	[2]
Staphylococcus aureus	*Ptilophora subcostata*	Dry powder	-	-	-	60 µl	>10 mm	-	[18]
	Rhodomela confervoides	-	Summer	-	-	Disc thallus	4,5 mm	-	[2]
		-	Autumn	-	-	Disc thallus	3 mm	-	[2]
		-	Winter	-	-	Disc thallus	3,5 mm	-	[2]
		-	Spring	-	-	Disc thallus	1 mm	-	[2]
	Saccharina japonica	SC-CO2 + Ethanol	-	High total fatty acids content, Elaidic acid, eicosapentaenoic acid and fucoxanthin	-	10 µL (100 µg/mL)	18 ± 0.50 mm	-	[40]
		Acetone: Methanol	-	High total fatty acids content + Elaidic acid, eicosapentaenoic acid and fucoxanthin	-	10 µL (100 µg/mL)	20 ± 0.50 mm	-	[40]

		Hexane	-	Elaidic acid, eicosapentaenoic acid and fucoxanthin	-	10 µL (100 µg/mL)	8 ± 0.40 mm	-	[40]
		Ethanol	-	Elaidic acid, eicosapentaenoic acid and fucoxanthin	-	10 µL (100 µg/mL)	6 ± 0.05 mm	-	[40]
	Saccharina latissima	-	Spring	-	-	Disc thallus	8,5 mm	-	[2]
		-	Winter	-	-	Disc thallus	11 mm	-	[2]
	Sargassum aquifolium	Fresh material (Ethanol_Liquid fraction)	-	Alkaloids, triterpenoid glycosides, phenols, saponins and volatile oils	-	-	39.40 ± 1.13 %	-	[41]
		Fresh material (Ethanol_Solid fraction)	-	Alkaloids, triterpenoid glycosides, phenols, saponins and volatile oils	-	-	38.72 ± 1.78 %	-	[41]
Staphylococcus aureus	*Sargassum aquifolium*	Dry material (Ethanol_Liquid fraction)	-	Alkaloids, triterpenoid glycosides, phenols, saponins and volatile oils	-	-	30.42 ± 4.89 %	-	[41]
		Dry material (Ethanol_Solid fraction)	-	Alkaloids, triterpenoid glycosides, phenols, saponins and volatile oils	-	-	29.51 ± 4.47 %	-	[41]
	Sargassum cristaefolium	Hexane	-	Phenol, 1-nonadecene, myristic acid, 9-tricosene (Z)-, palmitic acid, 1-hexacosene and oleic acid.	-	2 mg/mL	3.88±0.33 mm	-	[26]
	Sargassum cristaefolium	Serial extraction with hexane, and	-	Phenol, 1-hexadecene, myristic acid, 9-	-	2 mg/mL	3.42±0.47 mm	-	[26]

		then ethyl acetate		tricosene (Z)-, neophytadiene, phytol, cyclotetracosane, palmitic acid, and oleic acid.					
	Sargassum cristaefolium	Serial extraction with hexane, ethyl acetate and methanol, respectively.	-	Phenol, methyl dihydrojasmonate, 3-azepan-1-yl-benzo[d]isothiazole1,1-dioxide, hexyl cinnamic aldehyde, 9-tricosene(Z)-, palmitic acid, 1-hexacosene, hexatriacontane, and 1-docosene.	-	2 mg/mL	3.08±0.14 mm	-	[26]
	Sargassum desfontainesii	*n*-hexane	Autumn	-	-	5 µL	8 mm	-	[9]
		Methanol	Autumn	-	-	5 µL	10 mm	-	[9]
	Sargassum filipendula	Ethyl acetate	-	-	-	100 µL	0.50	Data of inhibition not specified	[4]
	Sargassum horneri	SC-CO2 + Ethanol	-	High total fatty acids content, palmitic acid, eicosapentaenoic acid and fucoxanthin	-	10 µL (100 µg/mL)	20 ± 0.45 mm	-	[40]
		Acetone + methanol	-	High total fatty acids content, palmitic acid, eicosapentaenoic acid and fucoxanthin	-	10 µL (100 µg/mL)	24 ± 0.10 mm	-	[40]
	Sargassum horneri	Hexane	-	Palmitic acid, eicosapentaenoic acid and fucoxanthin	-	10 µL (100 µg/mL)	10 ± 0.15 mm	-	[40]
		Ethanol	-	Palmitic acid, eicosapentaenoic	-	10 µL (100 µg/mL)	7 ± 0.04 mm	-	[40]

				acid and fucoxanthin					
	Sargassum horridum	Ethanol	-	-	Steroid compounds (such as fucosterol and its derivative saringosterol) present in all brown algae	20 mg/ml (100 µL)	10.6 %	-	[41]
Staphylococcus aureus	*Sargassum hystrix*	Ethyl acetate partition of Methanols	-	-	-	100 µL	0.52	Data of inhibition not specified	[4]
	Sargassum lanceolatum	Ethanol	-	-	-	2 mg/disc	8 mm	-	[12]
		Ethanol	-	-	-	4 mg/disc	8 mm	-	[12]
		Ethanol	-	-	-	6 mg/disc	9 mm	-	[12]
	Sargassum muticum	Methanol	-	Phenolic compounds (mainly flavonoids).	-	300 mg/mL	22.33 mm	-	[20]
		Water	-	-	-	300 mg/mL	9.67 mm	-	[20]
	Sargassum polycystum	Methanol	-	-	-	50 µL	20 mm	-	[30]
		Ethanol	-	Flavonoids and tannins	-	-	7.67 ± 1.15 mm	-	[33]
		Acetone	-	Tannins, steroids.	Tannins, flavonoids, terpenoids, cardiac glycosydes, phlobatannins and steroids	-	11	Graphical data (no units)	[19]
		Ethanol	-	Steroids	Flavonoids, terpenoids, cardiac glycosydes and steroids	-	9	Graphical data (no units)	[19]
		water extract	-	-	Phenols, amino acids and proteins	-	4	Graphical data (no units)	[19]
	Sargassum sp.	Hexane	-	-	-	0.1 mL	4-7 mm	-	[16]

	Sargassum tenerrimum	Water	-	-	-	-	1 mm	Graphical data	[21]
	Sargassum tenerrimum	Methanol	-	Amino acids, alkaloids, carbohydrates, saponins, sterols, terpenoids, proteins, and phenolic compounds (flavonoids, tannins)	-	-	13 mm	Graphical data	[21]
		Aqueous (gel-like liquid)	-	Tannins, flavonoids, alkaloids, steroids, saponins, phlorotannins and terpenoids.	-	40 µg/mL	1 mm	-	[17]
Staphylococcus aureus	*Sargassum tenerrimum*	Aqueous (gel-like liquid)	-	Tannins, flavonoids, alkaloids, steroids, saponins, phlorotannins and terpenoids.	-	60 µg/mL	3 mm	-	[17]
		Aqueous (gel-like liquid)	-	Tannins, flavonoids, alkaloids, steroids, saponins, phlorotannins and terpenoids.	-	80 µg/mL	4 mm	-	[17]
		Aqueous (gel-like liquid)	-	Tannins, flavonoids, alkaloids, steroids, saponins, phlorotannins and terpenoids.	-	100 µg/mL	5 mm	-	[17]

	Sargassum tenerrimum	Methanol	-	Cholest-5-en-3-ol, 24-propylidene-, (3á)-; 1,2-Benzenedicarboxylic acid, diisooctyl ester; Hentriacontane; 1-Docosene; 1-Nonadecene; 1-Hexadecanol; 1,2-Benzenediol; Benzoic acid.	-	Not specified.	20.3±1.5 mm	1,2-benzenedicarboxylic acid, diisooctyl ester, 1-docosene, 1,2-benzenediol and benzoic acid are indicated as the responsible compounds for the antibacterial activity.	[22]
	Sargassum tenerrimum	Petroleum ether	-	Benzoic acid, 3,5-dicyclohexyl-4-hydroxy-, methyl ester; Isomethadone; Cholesterol; Squalene; 9-Hexadecenoic acid, eicosyl ester, (Z)-; 17-Pentatriacontene; Dasycarpidan-1-methanol, acetate (ester); Hexadecanoic acid, methyl ester.	-	Not specified.	17.8±0.98 mm	Hexadecenoic acid, methyl ester, 17-pentatriacontene, dasycarpian-1-methanol, and acetate are indicated as the responsible compounds for the antibacterial activity.	[22]
	Sargassum wightii	Acetone	-	Steroids, terpenoids, glycosides, alkaloids, flavonoids, tannins and saponins	-	-	13±1.6 mm	-	[24]
		Diethyl ether	-	Steroids, terpenoids, glycosides, flavonoids and saponins	-	-	16.3±1.2 mm	-	[24]

		Methanol	-	Steroids, terpenoids, alkaloids, flavonoids, tannins and saponins	-	-	16±1.3 mm	-	[24]
Staphylococcus aureus	*Sphondylothamnion multifidum*	-	Summer	-	-	Disc thallus	21 mm	-	[2]
		-	Autumn	-	-	Disc thallus	22,5 mm	-	[2]
	Stoechospermum sp.	Ethanol	-	-	-	50 µl, 75 µl, 100 µl	8 mm	-	[31]
		Ethanol:chloroform (1:1)	-	-	-	50 µl, 75 µl, 100 µl	3 mm	-	[31]
	Stypopodium zonale	*n*-hexane	Autumn	-	-	5 µL	12 mm	-	[9]
		n-hexane	Winter	-	-	5 µL	10 mm	-	[9]
		Ethyl acetate	Autumn	-	-	5 µL	9 mm	-	[9]
		Methanol	Autumn	-	-	5 µL	11 mm	-	[9]
	Symphyocladiella parasitica	-	Summer	-	-	Disc thallus	3,5 mm	-	[2]
	Turbinaria ornata	Methanol	-	-	-	0.1 mL	2-3 mm	-	[16]
		Hexane	-	-	-	0.1 mL	2-3 mm	-	[16]
		Ethanol	-	High content in flavonoids and tannins	-	-	9.3 ± 0.7 mm	-	[33]
	Ulva lactuca	Ethanol (crude)	-	Abundance of alcohols and phenols	-	25 µL (holding capacity)	5 ± 4.08 mm	-	[32]
		Fraction 1 (Chloroform:methanol)	-	-	-	25 µL (holding capacity)	4 ± 0.07 mm	-	[32]
		Fraction 2 (Chloroform:methanol)	-	-	-	25 µL (holding capacity)	5 ± 0.06 mm	-	[32]
		Fraction 3 (Chloroform:methanol)	-	-	-	25 µL (holding capacity)	4 ± 0.88 mm	-	[32]
		-	Autumn	-	-	Disc thallus	2 mm	-	[2]
		-	Winter	-	-	Disc thallus	6,5 mm	-	[2]

		Methanol	-	-	-	25 µL	0-5 mm	-	[37]
		Diethyl ether	-	-	-	25 µL	0-5 mm	-	[37]
		Chloroform	-	-	-	25 µL	15 mm	-	[37]
Staphylococcus aureus	*Ulva linza*	Methanol	-	-	-	250 µg/mL (extract in DMSO)	10 mm	Antibacterial activity reported in bromophenols, carbonyls, halogenated aliphatic compounds, terpenes, isoprenylated and brominated hydroquinones, and phlorotannins	[13]
		Methanol	-	-	-	500 µg/mL (extract in DMSO)	11 mm	Antibacterial activity reported in bromophenols, carbonyls, halogenated aliphatic compounds, terpenes, isoprenylated and brominated hydroquinones, and phlorotannins	[13]
		Methanol	-	-	-	750 µg/mL (extract in DMSO)	14 mm	Antibacterial activity reported in bromophenols, carbonyls, halogenated aliphatic compounds, terpenes,	[13]

								isoprenylated and brominated hydroquinones, and phlorotannins	
Staphylococcus aureus	*Ulva linza*	Methanol	-	-	-	1000 µg/mL (extract in DMSO)	18 mm	Antibacterial activity reported in bromophenols, carbonyls, halogenated aliphatic compounds, terpenes, isoprenylated and brominated hydroquinones, and phlorotannins	[13]
	Ulva rigida	*n*-hexane	Winter	-	-	5 µL	8 mm	-	[9]
		Methanol	Autumn	-	-	5 µL	9 mm	-	[9]
		Methanol	Winter	-	-	5 µL	10 mm	-	[9]
	Ulva sp.	Methanol	-	-	-	58 µl, 75 µl, 100 µl	2 mm	-	-
	Vertebrata byssoides	-	Spring	-	-	Disc thallus	11 mm	-	[2]
	Vertebrata byssoides	-	Summer	-	-	Disc thallus	8,5 mm	-	[2]
		-	Autumn	-	-	Disc thallus	9 mm	-	[2]
	Vertebrata fucoides	-	Spring	-	-	Disc thallus	11 mm	-	[2]
		-	Summer	-	-	Disc thallus	11,5 mm	-	[2]
		-	Autumn	-	-	Disc thallus	11 mm	-	[2]
		-	Winter	-	-	Disc thallus	11 mm	-	[2]
	Vertebrata lanosa	-	Spring	-	-	Disc thallus	18 mm	-	[2]
		-	Summer	-	-	Disc thallus	18 mm	-	[2]
		-	Autumn	-	-	Disc thallus	19 mm	-	[2]
		-	Winter	-	-	Disc thallus	18,5 mm	-	[2]
		-	Spring (Sterile)	-	-	Disc thallus	18 mm	-	[36]
		-	Spring (Male)	-	-	Disc thallus	17 mm	-	[36]

		-	Summer (Sterile)	-	-	Disc thallus	18 mm	-	[36]
		-	Summer (Cystocarpic)	-	-	Disc thallus	18 mm	-	[36]
Staphylococcus aureus	*Vertebrata lanosa*	-	Summer (Tetrasporic)	-	-	Disc thallus	18,5 mm	-	[36]
	Vertebrata nigra	-	Spring	-	-	Disc thallus	10 mm	-	[2]
		-	Summer	-	-	Disc thallus	11 mm	-	[2]
		-	Autumn	-	-	Disc thallus	11 mm	-	[2]
		-	Winter	-	-	Disc thallus	11 mm	-	[2]
	Vertebrata thuyoides	-	Spring	-	-	Disc thallus	4,5 mm	-	[2]
		-	Summer	-	-	Disc thallus	6,5 mm	-	[2]
		-	Autumn	-	-	Disc thallus	6 mm	-	[2]
		-	Winter	-	-	Disc thallus	5 mm	-	[2]
Xanthomonas oryzae pv. *oryzae*	*Chnoospora minima*	Methanol	-	-	Polar substances	50 µL (250 µg)	3 mm	Graphical values	[42]
		Saponified	-	-	Polar substances	50 µL (250 µg)	2 mm	Graphical values	[42]
		Unsaponified	-	-	Polar substances	50 µL (250 µg)	2 mm	Graphical values	[42]
		Lipophilic	-	-	Polar substances	50 µL (250 µg)	2 mm	Graphical values	[42]
		Hexane:ethyl acetate (4:6 v/v)	Unsaponified fraction Fraction 0.40 (UV)	-	-	85 µL (250 µg)	3.3 ± 0.53 mm	-	[43]
		Hexane:diethyl ether:1% acetic acid (5:4:1 v/v/v)	Methanol Fraction 0.42 (iodine)	-	-	86 µL (250 µg)	7 mm	Graphical values	[43]
	Chnoospora minima	Hexane:diethyl ether:1% acetic acid (5:4:1 v/v/v)	Methanol Fraction 0.78 (UV)	-	-	87 µL (250 µg)	9 mm	Graphical values	[43]
		Hexane:diethyl ether:1% acetic acid (5:4:1 v/v/v)	Lipophilic fraction Fraction 0.37 (iodine)	-	-	88 µL (250 µg)	2 mm	Graphical values	[43]
		Hexane:diethyl ether:1% acetic acid (5:4:1 v/v/v)	Lipophilic fraction Fraction 0.55 (UV)	-	-	89 µL (250 µg)	4 mm	Graphical values	[43]
	Gracilaria blodgetti	Hexane:ethyl acetate (4:6 v/v)	Petroleum ether extracts Fraction 0.21	-	-	50 µL (250 µg)	6.6 ± 0.58 mm	-	[43]
		Petroleum ether	-	-	Non-polar substances	50 µL (250 µg)	5 mm	Graphical values	[42]

		Diethyl ether	-	-	Non-polar substances	50 µL (250 µg)	3 mm	Graphical values	[42]
		Chloroform	-	-	Non-polar substances	50 µL (250 µg)	3 mm	Graphical values	[42]
Xanthomonas oryzae pv. *oryzae*	*Gracilaria blodgetti*	Chloroform: Methanol	-	-	Non-polar substances	50 µL (250 µg)	3 mm	Graphical values	[42]
		Saponified	-	-	Non-polar substances	50 µL (250 µg)	4 mm	Graphical values	[42]
		Unsaponified	-	-	Non-polar substances	50 µL (250 µg)	6 mm	Graphical values	[42]
		Lipophilic	-	-	Non-polar substances	50 µL (250 µg)	5 mm	Graphical values	[42]
		Hexane:ethyl acetate (4:6 v/v)	Unsaponified fraction Fraction 0.20	-	-	51 µL (250 µg)	3.6 ± 0.58 mm	-	[43]
		Hexane:ethyl acetate (4:6 v/v)	Unsaponified fraction Fraction 0.42	-	-	52 µL (250 µg)	8.3 ± 0.56 mm	-	[43]
	Hydropuntia edulis	Petroleum ether	-	-	Non-polar substances	50 µL (250 µg)	6 mm	Graphical values	[42]
		Diethyl ether	-	-	Non-polar substances	50 µL (250 µg)	5 mm	Graphical values	[42]
		Chloroform	-	-	Non-polar substances	50 µL (250 µg)	4 mm	Graphical values	[42]
		Chloroform:methanol	-	-	Non-polar substances	50 µL (250 µg)	3 mm	Graphical values	[42]
	Hydropuntia edulis	Saponified	-	-	Non-polar substances	50 µL (250 µg)	5 mm	Graphical values	[42]
		Unsaponified	-	-	Non-polar substances	50 µL (250 µg)	7 mm	Graphical values	[42]
		Lipophilic	-	-	Non-polar substances	50 µL (250 µg)	6 mm	Graphical values	[42]
		Hexane:ethyl acetate (4:6 v/v)	Petroleum ether extracts Fraction 0.48	-	-	53 µL (250 µg)	8 ± 1.0 mm	-	[43]
		Hexane:ethyl acetate (4:6 v/v)	Unsaponified fraction Fraction 0.38	-	-	54 µL (250 µg)	10 mm	Graphical values	[43]
		Hexane:ethyl acetate (4:6 v/v)	Unsaponified fraction	-	-	55 µL (250 µg)	2 mm	Graphical values	[43]

			Fraction 0.46						
	Hypnea musciformis	Petroleum ether	-	-	Non-polar substances	50 µL (250 µg)	4 mm	Graphical values	[42]
Xanthomonas oryzae pv. *oryzae*	*Hypnea musciformis*	Diethyl ether	-	-	Non-polar substances	50 µL (250 µg)	3 mm	Graphical values	[42]
		Chloroform	-	-	Non-polar substances	50 µL (250 µg)	2 mm	Graphical values	[42]
		Chloroform:methanol	-	-	Non-polar substances	50 µL (250 µg)	2 mm	Graphical values	[42]
		Saponified	-	-	Non-polar substances	50 µL (250 µg)	3 mm	Graphical values	[42]
		Unsaponified	-	-	Non-polar substances	50 µL (250 µg)	4 mm	Graphical values	[42]
		Lipophilic	-	-	Non-polar substances	50 µL (250 µg)	4 mm	Graphical values	[42]
		Hexane:ethyl acetate (4:6 v/v)	Petroleum ether extracts Fraction 0.20	-	-	60 µL (250 µg)	4 mm	Graphical values	[43]
		Hexane:ethyl acetate (4:6 v/v)	Petroleum ether extracts Fraction 0.32	-	-	61 µL (250 µg)	2 mm	Graphical values	[43]
		Hexane:ethyl acetate (4:6 v/v)	Unsaponified fraction Fraction 0.34	-	-	62 µL (250 µg)	5 mm	Graphical values	[43]
		Hexane:ethyl acetate (4:6 v/v)	Unsaponified fraction Fraction 0.53	-	-	63 µL (250 µg)	4 mm	Graphical values	[43]
	Hypnea valentiae	Petroleum ether	-	-	Non-polar substances	50 µL (250 µg)	5 mm	Graphical values	[42]
		Diethyl ether	-	-	Non-polar substances	50 µL (250 µg)	3 mm	Graphical values	[42]
		Chloroform	-	-	Non-polar substances	50 µL (250 µg)	3 mm	Graphical values	[42]
		Chloroform: Methanol	-	-	Non-polar substances	50 µL (250 µg)	3 mm	Graphical values	[42]
		Saponified	-	-	Non-polar substances	50 µL (250 µg)	3 mm	Graphical values	[42]
		Unsaponified	-	-	Non-polar substances	50 µL (250 µg)	5 mm	Graphical values	[42]

		Lipophilic	-	-	Non-polar substances	50 µL (250 µg)	4 mm	Graphical values	[42]
		Hexane:ethyl acetate (4:6 v/v)	Petroleum ether extracts Fraction 0.23	-	-	64 µL (250 µg)	4 mm	Graphical values	[43]
Xanthomonas oryzae pv. *oryzae*	*Hypnea valentiae*	Hexane:ethyl acetate (4:6 v/v)	Petroleum ether extracts Fraction 0.35	-	-	65 µL (250 µg)	6 mm	Graphical values	[43]
		Hexane:ethyl acetate (4:6 v/v)	Unsaponified fraction Fraction 0.31	-	-	66 µL (250 µg)	6 mm	Graphical values	[43]
		Hexane:ethyl acetate (4:6 v/v)	Unsaponified fraction Fraction 0.44	-	-	67 µL (250 µg)	3 mm	Graphical values	[43]
	Padina boergesenii	Chloroform	-	-	Polar substances	50 µL (250 µg)	3 mm	Graphical values	[42]
		Methanol	-	-	Polar substances	50 µL (250 µg)	9 mm	Graphical values	[42]
		Ethanol	-	-	Polar substances	50 µL (250 µg)	4 mm	Graphical values	[42]
		Chloroform:methanol	-	-	Polar substances	50 µL (250 µg)	3 mm	Graphical values	[42]
		Saponified	-	-	Polar substances	50 µL (250 µg)	4 mm	Graphical values	[42]
		Unsaponified	-	-	Polar substances	50 µL (250 µg)	5 mm	Graphical values	[42]
		Lipophilic	-	-	Polar substances	50 µL (250 µg)	6 mm	Graphical values	[42]
		Hexane:diethyl ether:1% acetic acid (5:4:1 v/v/v)	Methanol Fraction 0.41 (UV)	-	-	68 µL (250 µg)	3.0 ± 0.0 mm	-	[43]
		Hexane:diethyl ether:1% acetic acid (5:4:1 v/v/v)	Methanol Fraction 0.44 (iodine)	-	-	69 µL (250 µg)	8.3 ± 0.58 mm	-	[43]
		Hexane:ethyl acetate (4:6 v/v)	Unsaponified fraction Fraction 0.17 (UV)	-	-	70 µL (250 µg)	1 mm	Graphical values	[43]
		Hexane:ethyl acetate (4:6 v/v)	Unsaponified fraction Fraction 0.39 (UV)	-	-	71 µL (250 µg)	8 mm	Graphical values	[43]
		Hexane:diethyl ether:1% acetic acid (5:4:1 v/v/v)	Lipophilic fraction Fraction 0.12 (UV)	-	-	72 µL (250 µg)	6.0 ± 0.0 mm	-	[43]
		Hexane:diethyl ether:1% acetic acid (5:4:1 v/v/v)	Lipophilic fraction Fraction 0.20 (iodine)	-	-	73 µL (250 µg)	1 mm	Graphical values	[43]

		Hexane:diethyl ether:1% acetic acid (5:4:1 v/v/v)	Lipophilic fraction Fraction 0.31 (UV)	-	-	74 µL (250 µg)	1 mm	Graphical values	[43]
Xanthomonas oryzae pv. *oryzae*	*Padina boergesenii*	Hexane:diethyl ether:1% acetic acid (5:4:1 v/v/v)	Lipophilic fraction Fraction 0.34 (UV)	-	-	75 µL (250 µg)	4 mm	Graphical values	[43]
		Hexane:diethyl ether:1% acetic acid (5:4:1 v/v/v)	Lipophilic fraction Fraction 0.40 (iodine)	-	-	76 µL (250 µg)	3 mm	Graphical values	[43]
	Sargassum wightii	Chloroform	-	-	Polar substances	50 µL (250 µg)	4 mm	Graphical values	[42]
		Methanol	-	-	Polar substances	50 µL (250 µg)	11 mm	Graphical values	[42]
		Ethanol	-	-	Polar substances	50 µL (250 µg)	6 mm	Graphical values	[42]
		Chloroform:methanol	-	-	Polar substances	50 µL (250 µg)	5 mm	Graphical values	[42]
		Saponified	-	-	Polar substances	50 µL (250 µg)	5 mm	Graphical values	[42]
		Unsaponified	-	-	Polar substances	50 µL (250 µg)	6 mm	Graphical values	[42]
		Lipophilic	-	-	Polar substances	50 µL (250 µg)	8 mm	Graphical values	[42]
		Hexane:diethyl ether:1% acetic acid (5:4:1 v/v/v)	Methanol Fraction 0.29 (iodine)	-	-	77 µL (250 µg)	11.8 ± 0.53 mm	-	[43]
		Hexane:diethyl ether:1% acetic acid (5:4:1 v/v/v)	Methanol Fraction 0.70 (UV)	-	-	78 µL (250 µg)	4 mm	Graphical values	[43]
		Hexane:diethyl ether:1% acetic acid (5:4:1 v/v/v)	Methanol Fraction 0.80 (UV)	-	-	79 µL (250 µg)	3 mm	Graphical values	[43]
		Hexane:diethyl ether:1% acetic acid (5:4:1 v/v/v)	Methanol Fraction 0.87 (iodine)	-	-	80 µL (250 µg)	4 mm	Graphical values	[43]
		Hexane:diethyl ether:1% acetic acid (5:4:1 v/v/v)	Methanol Fraction 0.94 (UV)	-	-	81 µL (250 µg)	4 mm	Graphical values	[43]
		Hexane:ethyl acetate (4:6 v/v)	Unsaponified fraction Fraction 0.27 (UV)	-	-	82 µL (250 µg)	7.0 ± 0.0 mm	-	[43]
		Hexane:diethyl ether:1% acetic acid (5:4:1 v/v/v)	Lipophilic fraction Fraction 0.26 (iodine)	-	-	83 µL (250 µg)	4 mm	Graphical values	[43]

		Hexane:diethyl ether:1% acetic acid (5:4:1 v/v/v)	Lipophilic fraction Fraction 0.61 (UV)	-	-	84 µL (250 µg)	8.8 ± 0.57 mm	-	[43]
Xanthomonas oryzae pv. *oryzae*	*Sargassum wightii*	Methanol	Fraction Hexane:Ethyl acetate (80:20)	-	-	50 µL (100 µg of substances)	5.0±1.0 mm	-	[44]
		Methanol	Fraction Hexane:Ethyl acetate (60:40)	-	-	50 µL (100 µg of substances)	6.0±1.0 mm	-	[44]
		Methanol	Fraction Hexane:Ethyl acetate (20:80)	-	-	50 µL (100 µg of substances)	3.0±0.56 mm	-	[44]
		Methanol	Fraction Ethyl acetate:methanol (100:0)	-	-	50 µL (100 µg of substances)	5.3±0.56 mm	-	[44]
		Methanol	Fraction Ethyl acetate:methanol (40:60)	-	-	50 µL (100 µg of substances)	13.5±1.2 mm	-	[44]
	Spyridia hypnoides	Petroleum ether	-	-	Non-polar substances	50 µL (250 µg)	3 mm	Graphical values	[42]
		Diethyl ether	-	-	Non-polar substances	50 µL (250 µg)	2 mm	Graphical values	[42]
		Chloroform	-	-	Non-polar substances	50 µL (250 µg)	2 mm	Graphical values	[42]
		Chloroform:Methanol	-	-	Non-polar substances	50 µL (250 µg)	2 mm	Graphical values	[42]
		Saponified	-	-	Non-polar substances	50 µL (250 µg)	2 mm	Graphical values	[42]
		Unsaponified	-	-	Non-polar substances	50 µL (250 µg)	4 mm	Graphical values	[42]
		Lipophilic	-	-	Non-polar substances	50 µL (250 µg)	2 mm	Graphical values	[42]
		Hexane:ethyl acetate (4:6 v/v)	Unsaponified fraction Fraction 0.26	-	-	56 µL (250 µg)	4.3 ± 0.58 mm	-	[43]
		Hexane:ethyl acetate (4:6 v/v)	Unsaponified fraction Fraction 0.33	-	-	57 µL (250 µg)	3 ± 1.0 mm	-	[43]
		Hexane:ethyl acetate (4:6 v/v)	Petroleum ether extracts Fraction 0.24	-	-	58 µL (250 µg)	4,5 mm	Graphical values	[43]

		Hexane:ethyl acetate (4:6 v/v)	Petroleum ether extracts Fraction 0.46	-	-	59 µL (250 µg)	4 mm	Graphical values	[43]
Xanthomonas oryzae pv. *oryzae*	*Turbinaria conoides*	Hexane:ethyl acetate (4:6 v/v)	Unsaponified fraction Fraction 0.18 (UV)	-	-	90 µL (250 µg)	3.6 ± 0.58 mm	-	[43]
		Hexane:diethyl ether:1% acetic acid (5:4:1 v/v/v)	Methanol Fraction 0.22 (iodine)	-	-	91 µL (250 µg)	8 mm	Graphical values	[43]
		Hexane:diethyl ether:1% acetic acid (5:4:1 v/v/v)	Methanol Fraction 0.44 (iodine)	-	-	92 µL (250 µg)	4 mm	Graphical values	[43]
		Hexane:diethyl ether:1% acetic acid (5:4:1 v/v/v)	Methanol Fraction 0.57 (UV)	-	-	93 µL (250 µg)	5 mm	Graphical values	[43]
		Hexane:diethyl ether:1% acetic acid (5:4:1 v/v/v)	Lipophilic fraction Fraction 0.09 (UV)	-	-	94 µL (250 µg)	3 mm	Graphical values	[43]
		Hexane:diethyl ether:1% acetic acid (5:4:1 v/v/v)	Lipophilic fraction Fraction 0.22 (iodine)	-	-	95 µL (250 µg)	3 mm	Graphical values	[43]
		Chloroform	-	-	Polar substances	50 µL (250 µg)	4 mm	Graphical values	[42]
		Methanol	-	-	Polar substances	50 µL (250 µg)	9 mm	Graphical values	[42]
		Ethanol	-	-	Polar substances	50 µL (250 µg)	5 mm	Graphical values	[42]
	Turbinarina conoides	Chloroform:methanol	-	-	Polar substances	50 µL (250 µg)	4 mm	Graphical values	[42]
		Saponified	-	-	Polar substances	50 µL (250 µg)	5 mm	Graphical values	[42]
		Unsaponified	-	-	Polar substances	50 µL (250 µg)	5 mm	Graphical values	[42]
		Lipophilic	-	-	Polar substances	50 µL (250 µg)	6 mm	Graphical values	[42]
	Ulva flexuosa	Petroleum ether	-	-	Non-polar substances	50 µL (250 µg)	11 mm	Graphical values	[42]
		Diethyl ether	-	-	Non-polar substances	50 µL (250 µg)	10 mm	Graphical values	[42]
		Chloroform	-	-	Non-polar substances	50 µL (250 µg)	4 mm	Graphical values	[42]
		Chloroform:methanol	-	-	Non-polar substances	50 µL (250 µg)	5 mm	Graphical values	[42]
		Saponified	-	-	Non-polar substances	50 µL (250 µg)	6 mm	Graphical values	[42]

		Unsaponified	-	-	Non-polar substances	50 µL (250 µg)	12 mm	Graphical values	[42]
Xanthomonas oryzae pv. *oryzae*	*Ulva flexuosa*	Lipophilic	-	-	Non-polar substances	50 µL (250 µg)	8 mm	Graphical values	[42]
		Hexane:ethyl acetate (4:6 v/v)	Petroleum ether extracts Fraction 0.27	-	-	96 µL (250 µg)	12.3 ± 0.56 mm	-	[43]
		Hexane:ethyl acetate (4:6 v/v)	Petroleum ether extracts Fraction 0.49	-	-	97 µL (250 µg)	10 mm	Graphical values	[43]
		Hexane:ethyl acetate (4:6 v/v)	Petroleum ether extracts Fraction 0.66	-	-	98 µL (250 µg)	4 mm	Graphical values	[43]
		Hexane:ethyl acetate (4:6 v/v)	Unsaponified fraction Fraction 0.30	-	-	99 µL (250 µg)	13.7 ± 1.4 mm	-	[43]
		Hexane:ethyl acetate (4:6 v/v)	Unsaponified fraction Fraction 0.39	-	-	100 µL (250 µg)	2 mm	Graphical values	[43]
	Ulva lactuca	Petroleum ether	-	-	Non-polar substances	50 µL (250 µg)	2 mm	Graphical values	[42]
		Diethyl ether	-	-	Non-polar substances	50 µL (250 µg)	2 mm	Graphical values	[42]
		Saponified	-	-	Non-polar substances	50 µL (250 µg)	2 mm	Graphical values	[42]
		Unsaponified	-	-	Non-polar substances	50 µL (250 µg)	3 mm	Graphical values	[42]
		Lipophilic	-	-	Non-polar substances	50 µL (250 µg)	2 mm	Graphical values	[42]
		Hexane:ethyl acetate (4:6 v/v)	Petroleum ether extracts Fraction 0.34	-	-	101 µL (250 µg)	6.3 ± 0.56 mm	-	[43]
		Hexane:ethyl acetate (4:6 v/v)	Petroleum ether extracts Fraction 0.20	-	-	102 µL (250 µg)	2 mm	Graphical values	[43]
		Hexane:ethyl acetate (4:6 v/v)	Unsaponified fraction Fraction 0.29	-	-	50 µL (250 µg)	2.0 ± 1.0 mm	-	[43]
		Hexane:ethyl acetate (4:6 v/v)	Unsaponified fraction Fraction 0.34	-	-	50 µL (250 µg)	7.5 ± 1.41 mm	-	[43]

Xanthomonas oryzae pv. *oryzae*	*Ulva lactuca*	Hexane:ethyl acetate (4:6 v/v)	Unsaponified Fraction 0.66	-	-	50 µL (250 µg)	3.3 ± 0.56 mm	-	[43]

*- Species not found in AlgaeBase database (https://www.algaebase.org/).

Table S2. Detailed information of antibacterial activity reported from disc diffusion method modified (bacterial-agar medium).

Antibacterial activity | Disc diffusion method modified (Bacterial-agar medium)*[2]							
**Phytopathogenic bacteria**	**Macroalgae source**	**Solvent used**	**Extract concentration**	**Halo inhibition (mm)**	**Medium**	**Incubation**	**Reference**
Pseudomonas aeruginosa	*Sargassum fusiforme*	Ethanolic extract	10 mg/mL	7.75 ± 0.5 mm	Nutrient agar medium (agar basal layer)	37ºC, 24h	[45]
Staphylococcus aureus	*Ulva australis*	Ethanolic extract		10.00 ± 0.00 mm			
	Ulva prolifera			7.67 ± 0.29 mm			
	Gloiopeltis furcata			10.83 ± 0.58 mm			
	Gracilariopsis lemaneiformis			12.50 ± 0.87 mm			
	Ishige okamurae			7.33 ± 0.58 mm			
	Sargassum fusiforme			10.83 ± 0.29 mm			

*[2]- Methodology described in Li et al., 2018 [45].

Table S3. Detailed information of antibacterial activity reported from liquid-dilution method.

Liquid-dilution method*[2]									
**Phytopathogenic bacteria**	**Macroalgae source**	**Extraction solvent**	**Collection conditions**	**Extract concentration**	**Bacterial growth (% of the control)**	**Initial bacterial suspension**	**Culture medium**	**Incubation conditions**	**Reference**
Erwinia carotovora	*Lessonia trabeculata*	50% Ethanolic extracts	Summer	10,000 ppm	50%	1 µL (10^5–10^6 UFC/mL)	Müeller-Hinton medium	27ºC, 6h	[46]
		50% Ethanolic extracts	Autumn	10,000 ppm	50-60%	1 µL (10^5–10^6 UFC/mL)	Müeller-Hinton medium	27ºC, 6h	[46]
Pseudomonas syringae	*Lessonia trabeculata*	50% Ethanolic extracts	Summer	10,000 ppm	40%	1 µL (10^5–10^6 UFC/mL)	Müeller-Hinton medium	27ºC, 6h	[46]
		50% Ethanolic extracts	Autumn	10,000 ppm	40%	1 µL (10^5–10^6 UFC/mL)	Müeller-Hinton medium	27ºC, 6h	[46]
	Macrocystis pyrifera	50% Ethanolic extracts	Springtime	10,000 ppm	50%	1 µL (10^5–10^6 UFC/mL)	Müeller-Hinton medium	27ºC, 6h	[46]

*[2]- Methodology described in Jiménez et al., 2011 [46].

Table S4. Detailed information of antibacterial activity from microdilution method.

Microdilution method								
**Phytopathogenic bacteria**	**Macroalgae source**	**Solvent used**	**Composition of the extract**	**Possible composition (comparative works)**	**MIC**	**Incubation conditions**	**Notes**	**Reference**
Bacillus cereus	*Sargassum vulgare*	Methanol	Alga with high content in carbohydrate	-	500 µg/mL	12h, 35ºC	-	[47]
				-	1000 µg/mL	24h, 35ºC	MBC (tryptic soy agar plates)	[47]
Bacillus subtilis	*Chaetomorpha linum*	Ethyl acetate	-	-	5 mg/mL	24h, 37ºC	-	[8]
		Acetone	-	-	1.25 mg/mL	24h, 37ºC	-	[8]
	Osmundaria serrata	Lanosol ethyl ether (LEE)	-	-	0.15±0.06 mg/mL	37±2°C, 24h	-	[48]
		Lanosol ethyl ether (LEE)	-	-	1.00±0.00 mg/mL	37±2°C, 24h	MBC	[48]
	Padina gymnospora	Hexane	-	-	5 mg/mL	24h, 37ºC	-	[8]
		Ethyl acetate	-	-	5 mg/mL	24h, 37ºC	-	[8]
		Acetone	-	-	2.5 mg/mL	24h, 37ºC	-	[8]
	Sargassum wightii	Hexane	-	-	5 mg/mL	24h, 37ºC	-	[8]
		Ethyl acetate	-	-	2.5 mg/mL	24h, 37ºC	-	[8]
		Acetone	-	-	2.5 mg/mL	24h, 37ºC	-	[8]
Erwinia amylovora	*Chaetomorpha linum*	Ethyl acetate	-	-	2.5 mg/mL	24h, 37ºC	-	[8]
		Acetone	-	-	1.25 mg/mL	24h, 37ºC	-	[8]
	Padina gymnospora	Hexane	-	-	5 mg/mL	24h, 37ºC	-	[8]
		Ethyl acetate	-	-	5 mg/mL	24h, 37ºC	-	[8]
		Acetone	-	-	2.5 mg/mL	24h, 37ºC	-	[8]
	Sargassum wightii	Hexane	-	-	5 mg/mL	24h, 37ºC	-	[8]
		Ethyl acetate	-	-	5 mg/mL	24h, 37ºC	-	[8]
		Acetone	-	-	2.5 mg/mL	24h, 37ºC	-	[8]
Pseudomonas aeruginosa	*Chaetomorpha linum*	Hexane	-	-	5 mg/mL	24h, 37ºC	-	[8]
	Fucus spiralis	Phlorotannins purified	-	-	31.3 mg/mL	37ºC, 18-24h	-	[49]
	Gongolaria nodicaulis	Phlorotannins purified	-	-	31.3 mg/mL	37ºC, 18-24h	-	[49]
	Grateloupia livida	Petroleum ether	Organic acid ester, fatty acids, sterol, amide compounds	-	4 mg/ml	24h, 37ºC	-	[50]

	Osmundaria serrata	Lanosol ethyl ether (LEE)	-	-	0.42±0.08 mg/mL	37±2°C, 24h	-	[48]
		Lanosol ethyl ether (LEE)	-	-	0.42±0.08 mg/mL	37±2°C, 24h	MBC	[48]
Pseudomonas aeruginosa	*Padina gymnospora*	Ethyl acetate	-	-	2.5 mg/mL	24h, 37ºC	-	[8]
		Acetone	-	-	1.25 mg/mL	24h, 37ºC	-	[8]
		Methanol	Fatty acids and their derivatives	-	10 µg/mL	48h, 30ºC	-	[51]
	Sargassum wightii	Ethyl acetate	-	-	2.5 mg/mL	24h, 37ºC	-	[8]
		Acetone	-	-	2.5 mg/mL	24h, 37ºC	-	[8]
Pseudomonas sp.	*Sarconema filiforme*	Acetone	Alkaloids, phenolic compounds (flavonoids), proteins, sugars	-	12.5 µg/mL	24h, 37ºC	MBC	[52]
		Acetone	Alkaloids, phenolic compounds (flavonoids), proteins, sugars	-	25 µg/mL	24h, 37ºC	MBC	[52]
		Diethyl ether	-	-	-	24h, 37ºC	-	[52]
		Diethyl ether	-	-	-	24h, 37ºC	MBC	[52]
	Sargassum wightii	Acetone	Phenolic compounds (flavonoids, coumarins, tannins), steroids	-	25 µg/mL	24h, 37ºC	-	[52]
		Acetone	Phenolic compounds (flavonoids, coumarins, tannins) steroids	-	50 µg/mL	24h, 37ºC	MBC	[52]
Staphylococcus aureus	*Ascophyllum nodossum*	Acetone extract	-	Polyphenolic compounds	0.20 mg/mL	24h, 37ºC	-	[53]
	Bangia fuscopurpurea	*n*-butanol	-	-	500 µg/mL	Overnight, 37ºC	-	[34]
		Aqueous	-	-	500 µg/mL	Overnight, 37ºC	-	[34]
	Bifurcaria bifurcata	Dichloromethane extract	Lipophilic fraction (trimethylsilyl derivatives)	-	2048 µg/mL	24h, 37ºC	Synergistic effect with gentamicin and tetracycline	[54]
		Dichloromethane extract	Lipophilic fraction (trimethylsilyl derivatives)	-	1024 µg/mL	24h, 37ºC	-	[54]
	Callithamnion granulatum	Chloroform	-	Fatty acids (free or as acylglycerols)	250 µg/mL	Overnight, 37ºC	-	[34]
		n-butanol	-	-	500 µg/mL	Overnight, 37ºC	-	[34]

Staphylococcus aureus	*Callithamnion granulatum*	Aqueous	-	-	500 µg/mL	Overnight, 37ºC	-	[34]
		Chloroform	-	Fatty acids (free or as acylglycerols)	500 µg/mL	Overnight, 37ºC	-	[34]
		n-butanol	-	Monobrominated phenols	125 µg/mL	Overnight, 37ºC	-	[34]
		"Volatile compounds"	-	Aldehydes, free fatty acids, and phenol	250 µg/mL	Overnight, 37ºC	-	[34]
	Carradoriella elongata	Chloroform	-	Fatty acids (free or as acylglycerols)	250 µg/mL	Overnight, 37ºC	-	[34]
		n-butanol	-	Dibrominated phenols	250 µg/mL	Overnight, 37ºC	-	[34]
		Aqueous	-	-	250 µg/mL	Overnight, 37ºC	-	[34]
		"Volatile compounds"	-	Aldehydes, free fatty acids, and phenolic compounds	250 µg/mL	Overnight, 37ºC	Defensive compounds concentrated in the volatiles	[34]
		n-butanol	α-O-methyllanosol	-	125 µg/mL	Overnight, 37ºC	-	[34]
	Ceramium siliquosum var. *elegans*	Chloroform	-	Fatty acids (free or as acylglycerols)	250 µg/mL	Overnight, 37ºC	-	[34]
		n-butanol	1,2-dihydroxy ethane sulfonate,	-	250 µg/mL	Overnight, 37ºC	-	[34]
		Aqueous	-	-	250 µg/mL	Overnight, 37ºC	-	[34]
	Colpomenia peregrina	*n*-butanol	Mono-, di-, and tricarboxylic acids	Phenolic acids and their derivatives, an ester of phosphoric acid and significant amounts of free fatty acids	500 µg/mL	Overnight, 37ºC	-	[34]
	Colpomenia peregrina	"Volatile compounds"	Alkylated phenols, free lower fatty acids, benzoic acid, esters of acetic, formic acids, carvacrol, chlorinated hydrocarbons, sulfur containing compounds (methyl benzene sulfonamide)	-	500 µg/mL	Overnight, 37ºC	-	[34]
	Cystoseira compressa	Ethanol (50%)	High content of Palmitoleic acid isomer a; Hexadecenoic	-	5 mg/mL	24h	Collected in June (Minimal	[55]

			acid; Oleic acid; Heptadecanoic acid; Octadecanoic acid; Myristoleic acid methyl ester; 2-(1,2,2,2-Tetrahydroxyethoxy)ethane-1,1,1,2-tetrol; 1a,9b-Dihydrophenanthro[9,10-b]oxirene-2,3,4,7,8,9-hexacarbonitrile; D-Sorbitol; 9-Heptadecanoic acid; among others.				bactericidal concentration is equal to MIC)	
Staphylococcus aureus		Ethanol (50%)	High content of Palmitoleic acid isomer a; Hexadecenoic acid; Oleic acid; Heptadecanoic acid; Octadecanoic acid; Myristoleic acid methyl ester; 2-(1,2,2,2-Tetrahydroxyethoxy)ethane-1,1,1,2-tetrol; 1a,9b-Dihydrophenanthro[9,10-b]oxirene-2,3,4,7,8,9-hexacarbonitrile; 9-Heptadecanoic acid; among others.	-	2.5 mg/mL	24h	Collected in July (Minimal bactericidal concentration is equal to MIC)	[55]
		Ethanol (50%)	High content of Palmitoleic acid isomer a; Hexadecenoic acid; Oleic acid; Heptadecanoic acid; Octadecanoic acid; Myristoleic acid methyl ester; 2-(1,2,2,2-Tetrahydroxyethoxy)ethane-1,1,1,2-tetrol; 1a,9b-Dihydrophenanthro[9,10-b]oxirene-2,3,4,7,8,9-hexacarbonitrile; 9-Heptadecanoic acid; among others.	-	2.5 mg/mL	24h	Collected in August (Minimal bactericidal concentration is equal to MIC)	[55]

Staphylococcus aureus	*Ellisolandia elongata*	Chloroform	-	Fatty acids (free or as acylglycerols)	500 µg/mL	Overnight, 37ºC	-	[34]
		"Volatile compounds"	Phenol, 3,4-dihydroxy benzaldehyde, benzoic acid, monoterpene eucalyptol	-	500 µg/mL	Overnight, 37ºC	-	[34]
	Ericaria crinita	Chloroform	-	-	250 µg/mL	Overnight, 37ºC	-	[34]
		n-butanol	Mono-, di-, and tricarboxylic acids	Phenolic acids and their derivatives, an ester of phosphoric acid and significant amounts of free fatty acids	250 µg/mL	Overnight, 37ºC	-	[34]
		Aqueous	-	-	500 µg/mL	Overnight, 37ºC	-	[34]
	Ericaria selaginoides	Phlorotannins purified	-	-	15,6 mg/mL	37ºC, 18-24h	-	[49]
	Fucus spiralis	Phlorotannins purified	-	-	7,8 mg/mL	37ºC, 18-24h	-	[49]
	Gelidium spinosum	*n*-butanol	1,2-dihydroxy ethane sulfonate	Esters of phosphoric acid (1,2-dihydroxy ethane sulfonate)	250 µg/mL	Overnight, 37ºC	-	[34]
		Aqueous	-	-	250 µg/mL	Overnight, 37ºC	-	[34]
		"Volatile compounds"	Aldehydes	-	125 µg/mL	Overnight, 37ºC	-	[34]
	Gongolaria nodicaulis	Phlorotannins purified	-	-	7,8 mg/mL	37ºC, 18-24h	-	[49]
	Gongolaria usneoides	Phlorotannins purified	-	-	15,6 mg/mL	37ºC, 18-24h	-	[49]
	Grateloupia livida	Petroleum ether	Organic acid ester, fatty acids, sterol, amide compounds	-	2 mg/ml	24h, 37ºC	-	[50]
	Jania virgata	Chloroform	-	Fatty acids (free or as acylglycerols)	250 µg/mL	Overnight, 37ºC	-	[34]
		n-butanol	-	Monocarboxylic acids	125 µg/mL	Overnight, 37ºC	-	[34]
		"Volatile compounds"	Fatty acid methyl esters	-	125 µg/mL	Overnight, 37ºC	Defensive compounds concentrated in the volatiles	[34]

Staphylococcus aureus	*Laurencia coronopus*	Chloroform	-	Fatty acids (free or as acylglycerols)	500 µg/mL	Overnight, 37ºC	-	[34]
		n-butanol	-	N-containing compounds	250 µg/mL	Overnight, 37ºC	-	[34]
		Aqueous	-	-	500 µg/mL	Overnight, 37ºC	-	[34]
		"Volatile compounds"	-	-	500 µg/mL	Overnight, 37ºC	-	[34]
	Osmundaria serrata	Lanosol ethyl ether (LEE)	-	-	0.19±0.03 mg/mL	37±2°C, 24h	-	[48]
		Lanosol ethyl ether (LEE)	-	-	0.67±0.33 mg/mL	37±2°C, 24h	MBC	[48]
	Palisada perforata	Aqueous	-	-	250 µg/mL	Overnight, 37ºC	-	[34]
		"Volatile compounds"	-	-	500 µg/mL	Overnight, 37ºC	-	[34]
	Punctaria latifolia	Chloroform	-	-	250 µg/mL	Overnight, 37ºC	-	[34]
		n-butanol	Mono-, di-, and tricarboxylic acids	Phenolic acids and their derivatives, an ester of phosphoric acid and significant amounts of free fatty acids	250 µg/mL	Overnight, 37ºC	-	[34]
		Aqueous	-	-	500 µg/mL	Overnight, 37ºC	-	[34]
		"Volatile compounds"	Alkylated phenols, free lower fatty acids, benzoic acid, esters of acetic, formic acids, carvacrol, chlorinated hydrocarbons, sulfur containing compounds (methyl benzene sulfonamide)	-	500 µg/mL	Overnight, 37ºC	-	[34]
	Punctaria plantaginea	Chloroform	-	-	250 µg/mL	Overnight, 37ºC	-	[34]
		"Volatile compounds"	Alkylated phenols, free lower fatty acids, benzoic acid, esters of acetic, formic acids, carvacrol, chlorinated hydrocarbons, sulfur containing compounds	-	500 µg/mL	Overnight, 37ºC	-	[34]

			(methyl benzene sulfonamide)					
Staphylococcus aureus	*Sargassum vulgare*	Methanol	Seaweed with high content in carbohydrate	-	250 µg/mL	12h, 35ºC	-	[47]
		Methanol	Seaweed with high content in carbohydrate	-	500 µg/mL (tryptic soy agar plates)	24h, 35ºC	MBC (minimum bactericidal activity	[47]
	Scytosiphon lomentaria	Chloroform	-	-	250 µg/mL	Overnight, 37ºC	-	[34]
		n-butanol	Mono-, di-, and tricarboxylic acids	Phenolic acids and their derivatives, an ester of phosphoric acid and significant amounts of free fatty acids	250 µg/mL	Overnight, 37ºC	-	[34]
		Aqueous	-	-	500 µg/mL	Overnight, 37ºC	-	[34]
		"Volatile compounds"	Alkylated phenols, free lower fatty acids, benzoic acid, esters of acetic, formic acids, carvacrol, chlorinated hydrocarbons, sulfur containing compounds (methyl benzene sulfonamide)	-	250 µg/mL	Overnight, 37ºC	-	[34]
	Stilophora tenella	Chloroform	-	-	500 µg/mL	Overnight, 37ºC	-	[34]
		n-butanol	Mono-, di-, and tricarboxylic acids	Phenolic acids and their derivatives, an ester of phosphoric acid and significant amounts of free fatty acids	500 µg/mL	Overnight, 37ºC	-	[34]
		Aqueous	-	-	500 µg/mL	Overnight, 37ºC	-	[34]
	Zanardinia typus	Chloroform	-	-	125 µg/mL	Overnight, 37ºC	-	[34]
		n-butanol	Mono-, di-, and tricarboxylic acids	Phenolic acids and their derivatives, an ester of phosphoric acid and significant amounts of free fatty acids	500 µg/mL	Overnight, 37ºC	-	[34]

Staphylococcus aureus	*Zanardinia typus*	Aqueous	-	-	500 µg/mL	Overnight, 37ºC	-	[34]
		"Volatile compounds"	Alkylated phenols, free lower fatty acids, benzoic acid, esters of acetic, formic acids, carvacrol, chlorinated hydrocarbons, sulfur containing compounds (methyl benzene sulfonamide)	-	500 µg/mL	Overnight, 37ºC	-	[34]

MIC defined as the lowest concentration inhibiting the visual growth of the test culture on the microplate.
MBC – minimum bactericidal activity.

Table S5. Detailed information of antibacterial activity reported by a spectrophotometric method.

Spectrophotometric method*[3]						
**Phytopathogenic bacteria**	**Macroalgae source**	**Extraction solvent**	**Decrease of growth (%)**	**Initial bacterial concentration (determined by OD in the nutrient media TSB12 *[4])**	**Incubation conditions**	**Reference**
Bacillus subtilis	*Bonnemaisonia hamifera*	Dichloromethane extract (dissolved in methanol)	20-49 %	0.03 (nutrient media: TSB12)	5h, 36ºC (shaking)	[56]
	Callithamnin corymbosum	Dichloromethane extract (dissolved in methanol)	50-79 %	0.03 (nutrient media: TSB12)	5h, 36ºC (shaking)	[56]
	Carradoriella elongata	Dichloromethane extract (dissolved in methanol)	20-49 %	0.03 (nutrient media: TSB12)	5h, 36ºC (shaking)	[56]
	Ceramium tenuicorne	Dichloromethane extract (dissolved in methanol)	20-49 %	0.03 (nutrient media: TSB12)	5h, 36ºC (shaking)	[56]
	Ceramium virgatum	Dichloromethane extract (dissolved in methanol)	50-79 %	0.03 (nutrient media: TSB12)	5h, 36ºC (shaking)	[56]
	Cladophora rupestris	Dichloromethane extract (dissolved in methanol)	20-49 %	0.03 (nutrient media: TSB12)	5h, 36ºC (shaking)	[56]
	Dasya baillouviana	Dichloromethane extract (dissolved in methanol)	50-79 %	0.03 (nutrient media: TSB12)	5h, 36ºC (shaking)	[56]
	Dumontia contorta	Dichloromethane extract (dissolved in methanol)	20-49 %	0.03 (nutrient media: TSB12)	5h, 36ºC (shaking)	[56]
	Fucus serratus	Dichloromethane extract (dissolved in methanol)	50-79 %	0.03 (nutrient media: TSB12)	5h, 36ºC (shaking)	[56]
	Fucus vesiculosus	Dichloromethane extract (dissolved in methanol)	50-79 %	0.03 (nutrient media: TSB12)	5h, 36ºC (shaking)	[56]
	Halosiphon tomentosus	Dichloromethane extract (dissolved in methanol)	50-79 %	0.03 (nutrient media: TSB12)	5h, 36ºC (shaking)	[56]
	Rhodomela confervoides	Dichloromethane extract (dissolved in methanol)	50-79 %	0.03 (nutrient media: TSB12)	5h, 36ºC (shaking)	[56]
	Saccharina latissima	Dichloromethane extract (dissolved in methanol)	> 80 %	0.03 (nutrient media: TSB12)	5h, 36ºC (shaking)	[56]
	Vertebrata nigra	Dichloromethane extract (dissolved in methanol)	> 80%	0.03 (nutrient media: TSB12)	5h, 36ºC (shaking)	[56]
Pseudomonas aeruginosa	*Ceramium tenuicorne*	Dichloromethane extract (dissolved in methanol)	50-79 %	0.03 (nutrient media: TSB12)	5h, 36ºC (shaking)	[56]

***[3]**-Methodology described in Goecke et al., 2012 [56].

***[4]**-Nutrient media TSB12 prepared with 12 g/L Difco tryptic soy broth, 10 g/L NaCl, pH 7.2 in distilled water.

Table S6. Detailed information about antibacterial activity from field studies.

Field Studies								
**Phytopathogenic Bacteria**	**Macroalgae source**	**Specifications of the extract**	**Extract concentration**	**Extract application**	**Host**	**Disease incidence (%)**	**Positive control**	**Reference**
Ralstonia solaneacearum	Brown seaweed extracts (Shanghai Redbrillian chemical, Batch no. 20140920)	Commercial product	1 g/L	Presoaking	Potato	3.33 ± 7.9 %	51.7 ± 8.3 %	[57]
		Commercial product	1 g/L	Spraying	Potato	0 %	51.7 ± 8.3 %	[57]
		Commercial product	1 g/L	Presoaking+ spraying	Potato	0 %	51.7 ± 8.3 %	[57]
Xanthomonas campestris pv. *vesicatoria*	*Ascophyllum nodosum* (Acadian SeaPlants Ltd., Dartmouth, NS, Canada)	Alkaline extract (Commercial product)	0,50%	Spraying of plants (field grown)	Tomato	45-50%	85-90%	[58]
		Alkaline extract (Commercial product)	0,50%	Spraying of plants (field grown)	Tomato	50-55%	75-80%	[58]
		Alkaline extract (Commercial product)	0,50%	Spraying of plants (greenhouse)	Tomato	25-30%	50-55%	[58]

References

[1] Esserti, S.; Smaili, A.; Rifai, L. A.; Koussa, T.; Makroum, K.; Belfaiza, M.; Kabil, E. M.; Faize, L.; Burgos, L.; Alburquerque, N.; et al. Protective effect of three brown seaweed extracts against fungal and bacterial diseases of tomato. *J. Appl. Phycol.*, **2017**, *29*, 1081–1093. https://doi.org/10.1007/s10811-016-0996-z.

[2] Hornsey, I. S.; Hide, D. The production of antimicrobial compounds by British marine algae I. Antibiotic-producing marine algae. *Br. Phycol. J.*, **1974**, *9*, 353–361. https://doi.org/10.1080/00071617400650421.

[3] Malini, M.; Ponnanikajamideen, M.; Malarkodi, C.; Rajeshkumar, S. Explore the Antimicrobial Potential from Organic Solvents extract of Brown Seaweed (*Sargassum longifolium*) alleviating to pharmaceuticals. *Int. J. Pharm. Res.*, **2014**, *6* (1), 28–35.

[4] Morales, J. L.; Cantillo-Ciau, Z. O.; Sánchez-Molina, I.; Mena-Rejón, G. J. Screening of Antibacterial and Antifungal Activities of Six Marine Macroalgae from Coasts of Yucatán Peninsula. *Pharm. Biol.*, **2006**, *44* (8), 632–635. https://doi.org/10.1080/13880200600897569.

[5] Freile-Pelegrín, Y.; Morales, J. L. Antibacterial activity in marine algae from the coast of Yucatan, Mexico. *Bot. Mar.*, **2004**, *47*, 140–146.

[6] Crasta, P. J.; Raviraja, N. S.; Sridhar, K. R. Antimicrobial activity of some marine algae of southwest coast of India. *Indian J. Mar. Sci.*, **1997**, *26*, 201–205.

[7] Sivakumar, S. M.; Safhi, M. M. Isolation and screening of bioactive principle from *Chaetomorpha antennina* against certain bacterial strains. *Saudi Pharm. J.*, **2013**, *21* (1), 119–121. https://doi.org/10.1016/j.jsps.2012.02.003.

[8] Rosaline, X. D.; Sakthivelkumar, S.; Rajendran, K.; Janarthanan, S. Screening of selected marine algae from the coastal Tamil Nadu, South India for antibacterial activity. *Asian Pac. J. Trop. Biomed.*, **2012**, *2* (1), S140–S146. https://doi.org/10.1016/S2221-1691(12)60145-2.

[9] Febles, C. I.; Arias, A.; Hardisson, A.; López, A. S.; Gil-Rodríguez, M. C. Antimicrobial Activity of Extracts from Some Canary Species of *Phaeophyta* and *Chlorophyta*. *Phyther. Res.*, **1995**, *9*, 385–387.

[10] Rizvi, M. A.; Shameel, M. Pharmaceutical Biology of Seaweeds from the Karachi Coast of Pakistan. *Pharm. Biol.*, **2005**, *43* (2), 97–107. https://doi.org/10.1080/13880200590919366.

[11] Andriani, Y.; Syamsumir, D. F.; Yee, T. C.; Harisson, F. S.; Herng, G. M.; Abdullah, S. A.; Orosco, C. A.; Ali, A. M.; Latip, J.; Kikuzaki, H.; et al. Biological Activities of Isolated Compounds from Three Edible Malaysian Red Seaweeds, *Gracilaria Changii*, *G. Manilaensis* and *Gracilaria* sp. *Nat. Prod. Commun.*, **2016**, *11* (8), 1117–1120. https://doi.org/10.1177/1934578X1601100822.

[12] Ambreen, A.; Khan, H.; Tariq, A.; Ruqqia, A.; Sultana, V.; Ara, J. Evaluation of Biochemical Component and Antimicrobial Activity of Some Seaweeeds Occurring at Karachi Coast. *Pakistan J. Bot.*, **2012**, *44* (5), 1799–1803.

[13] Narasimhan, M. K.; Pavithra, S. K.; Krishnan, V.; Chandrasekaran, M. In vitro Analysis of Antioxidant, Antimicrobial and Antiproliferative Activity of *Enteromorpha antenna*, *Enteromorpha linza* and *Gracilaria corticata* Extracts. *Jundishapur J. Nat. Pharm. Prod.*, **2013**, *8* (4), 151–159. https://doi.org/10.17795/jjnpp-11277.

[14] Arulkumar, A.; Rosemary, T.; Paramasivam, S.; Rajendran, R. B. Phytochemical composition, in vitro antioxidant, antibacterial potential and GC-MS analysis of red seaweeds (*Gracilaria corticata* and *Gracilaria edulis*) from Palk Bay, India. *Biocatal. Agric. Biotechnol.*, **2018**, *15*, 63–71. https://doi.org/10.1016/j.bcab.2018.05.008.

[15] Capillo, G.; Savoca, S.; Costa, R.; Sanfilippo, M.; Rizzo, C.; Giudice, A. Lo; Albergamo, A.; Rando, R.; Bartolomeu, G.; Spanò, N.; et al. New Insights into the Culture Method and Antibacterial Potential of *Gracilaria gracilis*. *Mar. Drugs*, **2018**, *16* (492), 1–21. https://doi.org/10.3390/md16120492.

[16] Alam, K.; Agua, T.; Maven, H.; Taie, R.; Rao, K. S.; Burrows, I.; Huber, M. E.; Rali, T. Preliminary Screening of Seaweeds, Seagrass and Lemongrass Oil from Papua New Guinea for Antimicrobial and Antifungal Activity. *Int. J. Pharmacogn.*, **1994**, *32* (4), 396–399.

[17] Banu, A. T.; Ramani, P. S.; M., A. Effect of seaweed coating on quality characteristics and shelf life of tomato (*Lycopersicon esculentum mill*). *Food Sci. Hum. Wellness*, **2020**. https://doi.org/10.1016/j.fshw.2020.03.002

[18] Ogasawara, K.; Yamada, K.; Hatsugai, N.; Imada, C.; Nishimura, M. Hexose Oxidase-Mediated Hydrogen Peroxide as a Mechanism for the Antibacterial Activity in the Red Seaweed *Ptilophora subcostata*. *PLoS One*, **2016**, *11* (2), 1–11. https://doi.org/10.1371/journal.pone.0149084.

[19] Perumal, B.; Chitra, R.; Maruthupandian, A.; Viji, M. Nutritional assessment and bioactive potential of *Sargassum polycystum* C. Agardh (Brown Seaweed). *Indian J. Geo-Marine Sci.*, **2019**, *48* (4), 492–498.

[20] Nofal, A.; Azzazy, M.; Ayyad, S.; Abdelsalm, E.; Abousekken, M. S.; Tammam, O. Evaluation of the brown alga, *Sargassum muticum* extract as an antimicrobial and feeding additives. *Brazilian J. Biol.*, **2022**, *84*, 1–9.

[21] Kumar, P.; Selvi, S. S.; Prabha, A. L.; Kumar, K. P.; Ganeshkumar, R. S.; Govindaraju, M. Synthesis of silver nanoparticles from *Sargassum tenerrimum* and screening phytochemicals for its antibacterial activity. *Nano Biomed. Eng.*, **2012**, *4* (1), 12–16. https://doi.org/10.5101/nbe.v4i1.p12-16.

[22] Albratty, M.; Alhazmi, H. A.; Meraya, A. M.; Najmi, A.; Alam, M. S.; Rehman, Z.; Moni, S. S. Spectral analysis and Antibacterial activity of the bioactive principles of *Sargassum tenerrimum J. Agardh* collected from the Red sea, Jazan, Kingdom of Saudi Arabia. *Brazilian J. Biol.*, **2023**, *83*, 1–10.

[23] Kumaresan, M.; Anand, K. V.; Govindaraju, K.; Tamilselvan, S.; Kumar, V. G. Seaweed *Sargassum wightii* mediated preparation of zirconia (ZrO2) nanoparticles and their antibacterial activity against gram positive and gram negative bacteria. *Microb. Pathog.*, **2018**, *124*, 311–315. https://doi.org/10.1016/j.micpath.2018.08.060.

[24] Achary, A.; Muthalagu, K.; Guru, M. S. Identification of Phytochemicals from *Sargassum wightii* against *Aedes aegypti*. *Int. J. Pharm. Sci. Rev. Res.*, **2014**, *29* (1), 314–319.

[25] Lakhdar, F.; Boujaber, N.; Oumaskour, K.; Assobhei, O.; Etahiri, S. Inhibitive Activity of 17 marine algae from the coast of El Jadida-Morocco against *Erwinia chrysanthemi*. *Int. J. Pharm. Pharm. Sci.*, **2015**, *7* (10), 376–380.

[26] Susilo, B.; Rohim, A.; Wahyu, M. L. Serial Extraction Technique of Rich Antibacterial Compounds in *Sargassum cristaefolium* Using Different Solvents and Testing Their Activity. *Curr. Bioact. Compd.*, **2022**, *18* (3), 18–25. https://doi.org/10.2174/1573407217666210910095732.

[27] Rebecca, L. J.; Dhanalakshmi, V.; Thomas, T. A comparison between the effects of three algal extracts against pathogenic bacteria. *J. Chem. Pharm. Res.*, **2012**, *4* (11), 4859–4863.

[28] Belattmania, Z.; Reani, A.; Barakate, M.; Zrid, R.; Elatouani, S.; Hassouani, M.; Eddaoui, A.; Bentiss, F.; Sabour, B. Antimicrobial, antioxidant and alginate potentials of *Dictyopteris polypodioides* (Dictyotales, Phaeophyceae) from the Moroccan Atlantic coast. *Der Pharma Chem.*, **2016**, *8* (2), 216–226.

[29] Eahamban, K.; Antonisamy, J. M. Preliminary Phytochemical , UV-VIS, HPLC and Anti-bacterial Studies on *Gracilaria corticata J. Ag*. *Asian Pac. J. Trop. Biomed.*, **2012**, S568–S574. https://doi.org/10.1016/S2221-1691(12)60275-5.

[30] Thangaraju, N.; Venkatalakshmi, R. P.; Chinnasamy, A.; Kannaiyan, P. Synthesis of silver nanoparticles and the antibacterial and anticancer activities of the crude extract of *Sargassum polycystum* C . Agardh. *Nano Biomed. Eng.*, **2012**, *4* (2), 89–94. https://doi.org/10.5101/nbe.v3i1.p89-94.Introduction.

[31] Rebecca, L. J.; Dhanalakshmi, V.; Sharmila, S.; Das, M. P. Bactericidal Activity of *Stoechospermum* sp. *Res. J. Pharm. Biol. Chem. Sci. Bactericidal*, **2013**, *4* (2), 1749–1754.

[32] Radhika, D.; Mohaideen, A. Fourier transform infrared analysis of *Ulva Lactuca* and *Gracilaria Corticata* and their effect on antibacterial activity. *Asian J. Pharm. Clin. Res.*, **2015**, *8* (2).

[33] Bharath, B.; Pavithra, A. N.; Divya, A.; Perinbam, K. Chemical Composition of Ethanolic Extracts from some Seaweed Species of the South Indian Coastal Zone, Their Antibacterial and Membrane-Stabilizing Activity. *Russ. J. Mar. Biol.*, **2020**, *46* (5), 370–378. https://doi.org/10.1134/S1063074020050041.

[34] Kamenarska, Z.; Serkedjieva, J.; Najdenski, H.; Stefanov, K.; Tsvetkova, I.; Dimitrova-Konaklieva, S.; Popov, S. Antibacterial, antiviral, and cytotoxic activities of some red and brown seaweeds from the Black Sea. *Bot. Mar.*, **2009**, *52*, 80–86. https://doi.org/10.1515/BOT.2009.030.

[35] McConnell, O. J.; Fenical, W. Polyhalogenated 1-Octene-3-Ones, Antibacterial Metabolites from the Red Seaweed *Bonnemaisonia Asparagoides*. *Tetrahedron Lett.*, **1977**, *18* (22), 1851–1854.

[36] Hornsey, I. S.; Hide, D. The Production of Antimicrobial Compounds by British Marine Algae. IV. Variation of Antimicrobial Activity with Algal Generation. *Br. Phycol. J.*, **1985**, *20*, 21–25. https://doi.org/10.1080/00071618500650041.

[37] Zouaoui, B.; Ghalem, B. R. The Phenolic Contents and Antimicrobial Activities of Some Marine Algae from the Mediterranean Sea (Algeria). *Russ. J. Mar. Biol.*, **2017**, *43* (6), 491–495. https://doi.org/10.1134/S1063074017060128.

[38] Devi, K. P.; Suganthy, N.; Kesika, P.; Pandian, S. K. Bioprotective properties of seaweeds: *In vitro* evaluation of antioxidant activity and antimicrobial activity against food borne bacteria in relation to polyphenolic content. *BMC Complement. Altern. Med.*, **2008**, 1–11. https://doi.org/10.1186/1472-6882-8-38.

[39] Rajaboopathi, S.; Thambidurai, S. Evaluation of UPF and antibacterial activity of cotton fabric coated with colloidal seaweed extract functionalized silver nanoparticles. *J. Photochem. Photobiol. B Biol.*, **2018**, *183*, 75–87. https://doi.org/10.1016/j.jphotobiol.2018.04.028.

[40] Sivagnanam, S. P.; Yin, S.; Choi, J. H.; Park, Y. B.; Woo, H. C.; Chun, B. S. Biological Properties of Fucoxanthin in Oil Recovered from Two Brown Seaweeds Using Supercritical CO2 Extraction. *Mar. Drugs*, **2015**, *13*, 3422–3442. https://doi.org/10.3390/md13063422.

[41] Coronado, A. S.; Dionisio-Sese, M. L. Antimicrobial property of crude ethanolic extract from *Sargassum crassifolium*. *Asian J. Microbiol. Biotechnol. Environ. Sci.*, **2014**, *16* (3), 471–474.

[42] Kumar, K. A.; Rengasamy, R. Evaluation of Antibacterial Potential of Seaweeds Occurring along the Coast of Tamil Nadu, India against the Plant Pathogenic Bacterium *Xanthomonas oryzae* pv. *oryzae* (Ishiyama) Dye. *Bot. Mar.*, **2000**, *43*, 409–415.

[43] Kumar, K. A.; Rengasamy, R. Antibacterial Activities of Seaweed Extracts/Fractions Obtained through a TLC Profile against the Phytopathogenic Bacterium *Xanthomonas oryzae* pv. *oryzae*. *Bot. Mar.*, **2000**, *43*, 417–421.

[44] Arunkumar, K.; Selvapalam, N.; Rengasamy, R. The antibacterial compound sulphoglycerolipid 1-0 palmitoyl-3-0(6'-sulpho-alpha-quinovopyranosyl)-glycerol from *Sargassum wightii* Greville (Phaeophyceae). *Bot. Mar.*, **2005**, *48*, 441–445. https://doi.org/10.1515/BM.2005.058.

[45] Li, Y.; Sun, S.; Pu, X.; Yang, Y.; Zhu, F.; Zhang, S.; Xu, N. Evaluation of Antimicrobial Activities of Seaweed Resources from Zhejiang Coast, China. *Sustainability*, **2018**, *10* (2158), 1–12. https://doi.org/10.3390/su10072158.

[46] Jiménez, E.; Dorta, F.; Medina, C.; Ramírez, A.; Ramírez, I.; Peña-Cortés, H. Anti-Phytopathogenic Activities of Macro-Algae Extracts. *Mar. Drugs*, **2011**, *9*, 739–756. https://doi.org/10.3390/md9050739.

[47] Arguelles, E. D.; Monsalud, R. G.; Sapin, A. B. Chemical Composition and *In Vitro* Antioxidant and Antibacterial Activities of *Sargassum vulgare* C. Agardh from Lobo, Batangas, Philippines. *Int. Soc. Southeast Asian Agric. Sci.*, **2019**, *25* (1), 112–122.

[48] Barreto, M.; Meyer, J. J. M. Isolation and antimicrobial activity of a lanosol derivative from *Osmundaria serrata* (Rhodophyta) and a visual exploration of its biofilm covering. *South African J. Bot.*, **2006**, *72*, 521–528. https://doi.org/10.1016/j.sajb.2006.01.006.

[49] Lopes, G.; Sousa, C.; Silva, L. R.; Pinto, E.; Andrade, P. B.; Bernando, J.; Mouga, T.; Valentão, P. Can Phlorotannins Purified Extracts Constitute a Novel Pharmacological Alternative for

Microbial Infections with Associated Inflammatory Conditions? *PLoS One*, **2012**, *7* (2), 1–9. https://doi.org/10.1371/journal.pone.0031145.

[50] Jiang, Z.; Chen, Y.; Yao, F.; Chen, W.; Zhong, S.; Zheng, F.; Shi, G. Antioxidant, Antibacterial and Antischistosomal Activities of Extracts from *Grateloupia livida* (Harv). Yamada. *PLoS One*, **2013**, *8* (11), 1–10. https://doi.org/10.1371/journal.pone.0080413.

[51] Suresh, M.; Iyapparaj, P.; Anantharaman, P. Antifouling Activity of Lipidic Metabolites Derived from *Padina tetrastromatica*. *Appl. Biochem. Biotechnol.*, **2016**, *179*, 805–818. https://doi.org/10.1007/s12010-016-2032-9.

[52] Prakash, S.; Ahila, N. K.; Ramkumar, V. S.; Ravindran, J.; Kannapiran, E. Antimicrofouling properties of chosen marine plants: An eco-friendly approach to restrain marine microfoulers. *Biocatal. Agric. Biotechnol.*, **2015**, *4*, 114–121. https://doi.org/10.1016/j.bcab.2014.11.002.

[53] Jiménez, J. T.; O´Connell, S.; Lyons, H.; Bradley, B.; Hall, M. Antioxidant, antimicrobial, and tyrosinase inhibition activities of acetone extract of *Ascophyllum nodosum*. *Chem. Pap.*, **2010**, *64* (4), 434–442. https://doi.org/10.2478/s11696-010-0024-8.

[54] Santos, S. A. O.; Trindade, S. S.; Oliveira, C. S. D.; Parreira, P.; Rosa, D.; Duarte, M. F.; Ferreira, I.; Cruz, M. T.; Rego, A. M.; Abreu, M. H.; et al. Lipophilic Fraction of Cultivated *Bifurcaria bifurcata* R . Ross: Detailed Composition and In Vitro Prospection of Current Challenging Bioactive Properties. *Mar. Drugs*, **2017**, *15* (340), 1–17. https://doi.org/10.3390/md15110340.

[55] Čagalj, M.; Skroza, D.; Razola-Díaz, M. del C.; Verardo, V.; Bassi, D.; Frleta, R.; Mekinić, I. G.; Tabanelli, G.; Šimat, V. Variations in the Composition, Antioxidant and Antimicrobial Activities of *Cystoseira compressa* during Seasonal Growth. *Mar. Drugs*, **2022**, *20* (64), 1–14.

[56] Goecke, F.; Labes, A.; Wiese, J.; Imhoff, J. F. Dual effect of macroalgal extracts on growth of bacteria in Western Baltic Sea. *Rev. Biol. Mar. Oceanogr.*, **2012**, *47* (1), 75–86.

[57] Farag, S. M. A.; Elhalag, K. M. A.; Hagag, M. H.; Khairy, A. S. M.; Ibrahim, H. M.; Saker, M. T.; Messiha, N. A. S. Potato bacterial wilt suppression and plant health improvement after application of different antioxidants. *J. Phytopathol.*, **2017**, *165*, 522–537. https://doi.org/10.1111/jph.12589.

[58] Ali, N.; Ramkissoon, A.; Ramsubhag, A.; Jayaraj, J. *Ascophyllum* extract application causes reduction of disease levels in field tomatoes grown in a tropical environment. *Crop Prot.*, **2016**, *83*, 67–75. https://doi.org/10.1016/j.cropro.2016.01.016.
